# HCV screening in a cohort of HIV infected and uninfected homeless and marginally housed women in San Francisco, California

**DOI:** 10.1186/s12889-017-4102-5

**Published:** 2017-02-07

**Authors:** Kimberly Page, Michelle Yu, Jennifer Cohen, Jennifer Evans, Martha Shumway, Elise D. Riley

**Affiliations:** 1 Division of Epidemiology, Biostatistics & Preventive Medicine, Department of Internal Medicine, University of New Mexico Health Sciences Center, MSC10 5550; 1 University of New Mexico, Albuquerque, NM USA; 2 Department of Epidemiology & Biostatistics, University of California San Francisco, San Francisco, USA; 3 Department of Clinical Pharmacy, University of California San Francisco, San Francisco, USA; 4 Department of Psychiatry, University of California San Francisco, San Francisco, USA; 5 Department of Medicine, Division of HIV, Infectious Disease and Global Health, San Francisco General Hospital, University of California San Francisco, San Francisco, USA

**Keywords:** Hepatitis C virus, HCV, Women, Homeless, HIV coinfection, Mental health

## Abstract

**Background:**

Hepatitis C virus (HCV) screening has taken on new importance as a result of updated guidelines and new curative therapies. Relatively few studies have assessed HCV infection in homeless populations, and a minority include women. We assessed prevalence and correlates of HCV exposure in a cohort of homeless and unstably housed women in San Francisco, and estimated the proportion undiagnosed.

**Methods:**

A probability sample of 246 women were recruited at free meal programs, homeless shelters, and low-cost single room occupancy hotels in San Francisco; women with HIV were oversampled. At baseline, anti-HCV status was assessed using an enzyme immunoassay, and results compared in both HIV-positive and negative women. Exposures were assessed by self-report. Logistic regression was used to assess factors independently associated th HCV exposure.

**Results:**

Among 246 women 45.9% were anti-HCV positive, of whom 61.1% were HIV coinfected; 27.4% of positives reported no prior screening. Most (72%) women were in the ‘baby-boomer’ birth cohort; 19% reported recent injection drug use (IDU). Factors independently associated with anti-HCV positivity were: being born in 1965 or earlier (AOR) 3.94; 95%CI: 1.88, 8.26), IDU history (AOR 4.0; 95%CI: 1.68, 9.55), and number of psychiatric diagnoses (AOR 1.16; 95%CI: 1.08, 1.25).

**Conclusions:**

Results fill an important gap in information regarding HCV among homeless women, and confirm the need for enhanced screening in this population where a high proportion are baby-boomers and have a history of drug use and psychiatric problems. Due to their age and risk profile, there is a high probability that women in this study have been infected for decades, and thus have significant liver disease. The association with mental illness and HCV suggests that in addition increased screening, augmenting mental health care and support may enhance treatment success.

**Electronic supplementary material:**

The online version of this article (doi:10.1186/s12889-017-4102-5) contains supplementary material, which is available to authorized users.

## Background

Hepatitis C virus (HCV) infection is a widespread and chronic disease that is most prevalent among people who inject drugs (PWID) [1, 2], and which also disproportionately impacts racial and ethnic minorities [3–5], veterans [6], those who are incarcerated [7–9], the poor [10, 11], and unstably housed persons [12, 13]. A large number and proportion of HIV-infected patients are also co-infected with HCV [14]. Household-based surveillance puts estimates of the number of adult Americans ever infected with HCV at 3.6 million persons, and 2.7 million persons with chronic HCV [10]. However, since these surveys exclude the majority of high-risk populations, including incarcerated and homeless persons, the actual number is likely much higher, with an estimated 3.5 million current chronic infections (range 2.7 to 4.7 million) [15]. While a new generation of antiviral drugs (direct acting antivirals or DAAs) is transforming clinical outcomes, access remains a challenge, especially for these most affected groups, as a result of economic and other barriers, including low rates of screening, diagnosis and linkage to care [3]. The lack of data regarding HCV in these populations adds to the uncertainty regarding the burden of HCV infection and the potential impact DAAs could have on population health and even potential eradication [16].

Testing and identifying those with HCV are the most significant first steps needed to engage infected patients in care and provide access to curative treatments. Risk-based HCV testing guidance issued by the Centers for Disease Control and Prevention (CDC) in 1998, and the U.S. Public Health Service and Infectious Diseases Society of America in 1999 [17, 18], was updated in 2012 by the CDC to include those born between 1945 and 1965 (the “baby boomers”) [19]. It was endorsed by the United States Preventive Services Task Force in 2013 after research showed this age group accounts for a large proportion (75%) of chronic HCV infections diagnosed and have elevated risk of mortality [20, 21]. While several studies have noted that HCV infection is elevated in homeless or unstably housed adult populations women are generally underrepresented in these studies [12, 13]. The purpose of this study was to determine the prevalence and correlates of HCV exposure in a well-characterized cohort of homeless and marginally housed women, and estimate the proportion of women with undiagnosed HCV exposure.

## Methods

### Participants and setting

This study analyzed cross-sectional data from “Shelter, Health and Drug Outcomes among Women” (SHADOW), a prospective cohort study of homeless and unstably housed women in San Francisco [22]. Recruitment and enrollment occurred between June 2008 and August 2010. Using methods developed by Burman and Koegel [23], designed to recruit a representative sample of homeless individuals, women were systematically approached at free meal programs, homeless shelters, and low-cost single room occupancy (SRO) hotels selected with probability proportionate to the number of individuals served, and invited to participate in baseline screening at the study venue located in the Tenderloin neighborhood in downtown San Francisco. HIV-infected women were oversampled to meet the primary aims of the SHADOW study [22, 24]. Study inclusion criteria included female sex (biological), age ≥18 years, and a lifetime history of housing instability (slept in a public place, a shelter, or stayed with a series other people because they had no other place to sleep [“couch-surfed”]). Private interviews were conducted to collect data on demographic and social factors as shown in detail in the study survey (See Additional file 1). Sensitive questions regarding drug use and sex were obtained via audio computer-assisted self-interviews. Participants were interviewed every 6 months and reimbursed $15 for each interview completed. The study protocol and procedures were reviewed and approved by the Institutional Review Board at the University of California, San Francisco (UCSF). All participants completed a signed informed consent process.

### Measures

Data for the current analysis were collected during a cross-sectional sub-study that screened for co-infections including HCV, herpes simplex virus type 2 (HSV-2) and Trichomonas vaginalis, conducted between April and October, 2010. *Serologic testing:* The primary outcome for the current study was HCV infection exposure, including monoinfection and coinfection with HIV. Cohort participants were tested for anti-HCV using an enzyme immunoassay (EIA) test (EIA-3; OrthoClinical Diagnostics, Raritan, NJ) with a signal to cut off >8 indicating a positive result (Quest Diagnostics, San Francisco, CA). The test has 100% sensitivity and 99.95% specificity for detection of anti-HCV [25]. HIV status was determined using a point of care test (OraQuick®; OraSure Technologies, Bethlehem, Pennsylvania) and confirmatory Western Blot if positive. All women participating in the study were referred for care and additional evaluation if they had positive results for any infections. Specifically with respect to HCV, women were informed that that this was a screening test and that confirmatory testing would be needed to determine whether they had chronic infection.

*Exposures of interest:* Women were asked if they had ever been tested for a sexually transmitted infection (STI). Those who answered ‘yes’, were asked to indicate if they had ever tested for HCV, and if so what year they were tested. Women who answered ‘no’ to testing for any sexually transmitted infection (STI) or no to a previous positive anti-HCV were judged to be unaware of their HCV status and categorized as not having had a previous positive test. Biological, social and behavioral health factors known to be more prevalent among disadvantaged women compared to women in the general population were used in these analyses and included: unmet subsistence needs (insufficient access to food, clothing, a restroom, a place to wash or a place to sleep) [26]; instrumental social support (someone who would give money or a place to sleep) [27]; sexual orientation (heterosexual, bisexual or lesbian); and drug use, including injection drug use, and any use of crack cocaine, heroin or methamphetamine. Alcohol use was categorized as ‘at-risk’ if women reported drinking >1 drink/day [28]) and binge drinking (>4 drinks at one time for women) [29]. Multiple mental health measures were directly assessed by the computerized Diagnostic Interview Schedule (DIS)-IV, which uses DSM-IV criteria [30], including depression, and psychiatric disorders (anxiety, psychotic, mood, pain, somatization, substance use, dementia, and post-traumatic stress disorders) [30, 31]. Psychiatric diagnoses were grouped, and analyzed based on the number of diagnoses due to interactions between diagnoses among persons with co-occurring conditions [32].

### Analyses

Standard descriptive statistics were used to analyze socio-demographic, behavioral and clinical parameters by anti-HCV status, including monoinfection and HCV/HIV coinfection. Bivariate comparisons were conducted using the Kruskal-Wallis test for nonparametric continuous variables and Pearson chi-square (χ^2^) test or Fisher exact test (for cell sizes ≤5) for categorical variables. Odds ratios (OR) and 95% confidence intervals (CI) determined the magnitude of effect as well as the amount of variability in each estimate. Inferences were based on simultaneous adjustment for exposure variables using multiple logistic regression. To assess independent associations with HCV exposure, a backward stepwise approach was employed in which bivariate predictors with a *p*-value of 0.25 or less were included in the initial multivariable model, and variables were eliminated until all remaining parameter estimates had *p*-values less than 0.05 [33]. We used a *p*-value cut-off point of 0.25 for variable selection over more conservative levels such as 0.05 in order to maximize potential to identify important variables [34]. Multi-collinearity between explanatory variables was examined and effect modification considered. Adjusted Odds Ratios (AOR) were computed to examine excess risk in association with variables found to be independently associated with anti-HCV positivity.

## Results

Over 90% of eligible women agreed to participate in the cohort study, resulting in a sample of 300. Of those, 246 (82%) had concomitant interview and anti-HCV testing data. A total of 113 (45.9%) women had detectable anti-HCV, of whom 61.1% were HIV coinfected. Consistent with the recruitment strategy, just over half of the sample (*n* = 127; 51.6%) was HIV-infected. There were no significant differences between participants who did and did not have available data for the current analysis with regard to socioeconomic, sexual and drug use variables (*p* > 0.05). Median age in the total group was 48 years (Interquartile Range [IQR] 43, 54), but this varied significantly by HIV and HCV infection or coinfection group. Seventy-two percent of the sample corresponded to the ‘baby-boomer’ cohort (born between 1945 and 1965); median birth year for women who were HIV/HCV coinfected and HCV monoinfected was 1958, for HIV monoinfected women it was 1964, and uninfected women, 1962. Table 1 shows sociodemographic and health characteristics and recent risk exposures (in previous 6 months) of the study sample and associations with HCV and HIV infection and coinfection outcomes. More than half (54%) of women met diagnostic criteria for depression, recent crack use was reported by 46%, and 19% reported recent injection drug use (IDU). In addition to age, significant differences were found by education, recent transactional sex, having seen a primary care provider recently, number of psychiatric diagnoses, recent heroin use, injection drug use and HCV and HIV outcomes in bivariate analyses (Table 1). No differences were found in reported binge drinking, or marijuana use between groups.

**Table 1:** Prevalence of selected characteristics among all participants and associations with anti-HCV, HIV and HIV/HCV infection status

Variable	Prevalence of characteristic^b^(*N* = 246)		HIV/HCV coinfected (*N* = 69)		HCV mono-infected (*N* = 44)		HIV mono-infected (*N* = 58)		Non-infected (*N* = 75)		
	*N*	(%)	*N*	(%)	*N*	(%)	*N*	(%)	*N*	(%)	*p*-value^a^
# Completed surveys	246	(100)	69	(28)	44	(18)	58	(24)	75	(30)	
Reported a previous positive test for HCV^b^											
Yes	88	(36)	51	(75)	29	(67)	6	(10)	2	(3)	*<.0001*
No/Skipped	155	(64)	17	(25)	14	(33)	52	(90)	72	(97)	
Race											
Nonwhite	176	(72)	48	(27)	28	(16)	44	(25)	56	(32)	*0.5*
White	70	(28)	21	(30)	16	(23)	14	(20)	19	(27)	
Age, median (IQR)	48	(42–54)	50	(46–54)	50	(46–54)	45	(40–51)	47	(41–54)	*0.0092*
Birth cohort											
After 1965	69	(28)	10	(14)	10	(14)	25	(36)	24	(35)	*0.0029*
1965 and earlier	177	(72)	59	(33)	34	(19)	33	(19)	51	(29)	
High school graduate											
No	86	(35)	37	(43)	15	(17)	21	(24)	13	(15)	*0.0001*
Yes	160	(65)	32	(20)	29	(18)	37	(23)	62	(39)	
Sexual Orientation											
Heterosexual	190	(77)	52	(27)	31	(16)	49	(26)	58	(31)	*0.39*
Lesbian/Bi-sexual	56	(23)	17	(30)	13	(23)	9	(16)	17	(30)	
Recent income (past 6 months): $, median (IQR)	934	(812–1149)	908	(800–1025)	973	(800–1200)	935	(845–1175)	949	(659–1375)	*0.41*
Physical Health Score, median (IQR)	39	(30–48)	36	(29–45)	38	(29–49)	37	(30–47)	42	(32–52)	*0.13*
Mental Health Score, median (IQR)	43	(34–55)	43	(36–53)	43	(32–57)	43	(32–53)	45	(34–55)	*0.9*
Primary care visit in last 6 months											
Yes	203	(83)	64	(32)	31	(15)	55	(27)	53	(26)	*<.0001*
No	43	(17)	5	(12)	13	(30)	3	(7)	22	(51)	
Current depression											
Yes	129	(54)	30	(23)	21	(16)	35	(27)	43	(33)	*0.11*
No	108	(46)	38	(35)	21	(19)	20	(19)	29	(27)	
No. of psychiatric diagnoses, median (IQR)	7	(4–10)	9	(5–13)	9	(5–11)	7	(4–9)	5	(3–9)	*0.0005*
*Risk exposures in the past 6 months:*											
Incarcerated											
Yes	28	(11)	10	(36)	2	(7)	7	(25)	9	(32)	*0.42*
No	218	(89)	59	(27)	42	(19)	51	(23)	66	(30)	
Slept in a public place or homeless shelter											
Yes	59	(24)	14	(24)	11	(19)	12	(20)	22	(37)	*0.56*
No	187	(76)	55	(29)	33	(18)	46	(25)	53	(28)	
Unmet subsistence needs											
Yes	104	(42)	31	(30)	18	(17)	25	(24)	30	(29)	*0.94*
No	142	(58)	38	(27)	26	(18)	33	(23)	45	(32)	
Sex for money											
Yes	23	(9)	1	(4)	10	(43)	8	(35)	4	(17)	*0.0005*
No	223	(91)	68	(30)	34	(15)	50	(22)	71	(32)	
Sex for drugs											
Yes	15	(6)	1	(7)	4	(27)	6	(40)	4	(27)	*0.12*
No	231	(94)	68	(29)	40	(17)	52	(23)	71	(31)	
Heavy drinking											
Yes	50	(20)	16	(32)	11	(22)	11	(22)	12	(24)	*0.6*
No	196	(80)	53	(27)	33	(17)	47	(24)	63	(32)	
Binge drinking											
Yes	96	(39)	31	(32)	18	(19)	19	(20)	28	(29)	*0.55*
No	150	(61)	38	(25)	26	(17)	39	(26)	47	(31)	
Injection drug use											
Yes	47	(19)	18	(38)	14	(30)	7	(15)	8	(17)	*0.0071*
No	199	(81)	51	(26)	30	(15)	51	(26)	67	(34)	
Crack use											
Yes	114	(46)	38	(33)	22	(19)	25	(22)	29	(25)	*0.22*
No	132	(54)	31	(23)	22	(17)	33	(25)	46	(35)	
Cocaine use											
Yes	30	(12)	10	(33)	5	(17)	5	(17)	10	(33)	*0.76*
No	216	(88)	59	(27)	39	(18)	53	(25)	65	(30)	
Methamphetamine use											
Yes	51	(21)	12	(24)	11	(22)	9	(18)	19	(37)	*0.41*
No	195	(79)	57	(29)	33	(17)	49	(25)	56	(29)	
Heroin use											
Yes	31	(13)	13	(42)	10	(32)	4	(13)	4	(13)	*0.0074*
No	215	(87)	56	(26)	34	(16)	54	(25)	71	(33)	

^a^ *P* values for medians were obtained using the Kruskal-Wallis test; Categorical variable *p* values use either the Chi-square test (if all cell counts are greater than 5), or Fisher’s Exact test (if one or more cell counts less than or equal to 5)

^b^Percentages are column percents

Table 2 shows unadjusted correlates and a final multivariable logistic model of factors independently associated with HCV exposure. In the final model, birth cohort, less than high school education, number of psychiatric diagnoses, and a history of IDU were associated with elevated odds of anti-HCV positivity. Women who were born before 1965, and with a history of IDU had four times higher odds of HCV exposure. In addition, odds increased 12% for each additional psychiatric diagnosis, but current depression was associated with lower odds of HCV exposure.

**Table 2:** Social and Behavioral Correlates of anti-HCV positive status among homeless and unstably housed biological women living in San Francisco, CA (*N* = 237)

	Unadjusted	Adjusted^a^
	OR (95% CI)	OR (95% CI)
Non-white Race	0.68 (0.39–1.18)	
Age (per year increase)	1.05 (1.02–1.08) ***	
Birth cohort		
*After 1965*	1	
*1965 and earlier*	2.71 (1.49–4.93) ***	3.94 (1.88–8.26) ***
Less than high school graduate (vs high school graduate)	2.48 (1.45–4.25) ***	2.56 (1.36–4.82) ***
Sexual Orientation		
*Heterosexual*	1	
*Homo-/Bi-sexual*	1.49 (0.82–2.71)	
Recent income (per $100 increase)	0.97 (0.93–1.01)	
Physical Health Score (per 1 point increase)	0.98 (0.96–1) *	
Mental Health Score (per 1 point increase)	1.01 (0.99–1.03)	
Current Depression	0.54 (0.32–0.91) **	0.24 (0.12–0.48) ***
No. of psychiatric diagnoses (per 1+ increase)	1.12 (1.06–1.18) ***	1.16 (1.08–1.25) ***
Risk exposures in the past 6 months:		
Incarcerated (yes vs. no)	0.87 (0.39–1.92)	
Slept in a public place or homeless shelter (yes vs. no)	0.83 (0.46–1.49)	
Unmet subsistence needs (yes vs. no)	1.09 (0.65–1.8)	
Sex for money (yes vs. no)	1.09 (0.46–2.57)	
Sex for drugs (yes vs. no)	0.57 (0.15–1.9)	
Heavy drinking (yes vs. no)	1.5 (0.81–2.8)	
Injection drug use (yes vs. no)	3.11 (1.58–6.11) ***	4.0 (1.68–9.55) ***
Crack use (yes vs. no)	1.66 (1–2.75) *	
Cocaine use (yes vs. no)	1.2 (0.56–2.59)	
Methamphetamine use (yes vs. no)	0.96 (0.52–1.78)	
Heroin use (yes vs. no)	3.99 (1.71–9.33) ***	

^a^Adjusted for birth cohort, education, current depression, number of psychiatric diagnoses, and injection drug use (each adjusted for the others)

* p < .1

** p < .05

*** p < .01

Among the 246 women in the sample, 180 (73.2%) reported prior STI testing, and 88 (35.8%) women reported a previous positive HCV test. Among all women with positive serological results and who responded to questions regarding previous testing (*n* = 111 of 113 total positives), over a quarter (27.9%) reported a prior negative test, indicating almost one-third were unaware of their status. Twenty-five percent (17/68) of HIV/HCV coinfected women were not aware of their positive anti-HCV status, compared to 32.6% (14/43) of those with HCV monoinfection results (*p* = 0.4). Among women who tested anti-HCV negative in the study, including 58 with HIV infection and 74 uninfected women, almost all had no previous HCV positive result: 89.7 and 97.3%, respectively. Women who were anti-HCV positive and reported no previous test or a negative test were significantly (*p* < 0.02) older (median age: 52 years [IQR: 48, 56]) than those who reported a previous positive test (median age 49 years [IQR: 45, 53]). Among the 176 women born before 1965 (baby-boomer cohort), 30.1% (*n* = 53) reported not having a previous HCV test, and among the 93 HCV positive women in this age cohort, 31.2% (*n* = 29) were not aware of their anti-HCV status. Among 47 women who reported a history of IDU, 12.8% (*n* = 6) reported no previous HCV test, and among the 32 HCV positive women with a history of this exposure, 16% (*n* = 5) were not aware of their positive status. Women who were anti-HCV positive but had no previous HCV test result compared to those who had a previous positive, reported significantly lower median monthly income in the past 6 months ($853 [IQR: 770, 934] vs. $956 [IQR: 848, 1200], and fewer psychiatric diagnoses (5 [2, 10] vs. 9 [IQR: 6,13]), respectively. Factors independently associated with women not knowing their HCV status (controlling for birth cohort status and IDU) included: lower income (per $100 increase), AOR; 0.78 (95%CI: 0.65, 0.94); fewer psychiatric diagnosis (per diagnosis), AOR: 0.80 (95%CI: 0.70, 0.95); and any recent cocaine use (yes vs. no), AOR; 5.63 (95%CI: 1.16, 27.36).

## Discussion

HCV seroprevalence was very high in this sample of homeless and unstably housed women in San Francisco with almost half (45.9%) testing positive, and among whom 61.1% were HIV coinfected. These results fill an important gap in information since the majority of studies assessing HCV infection in homeless populations have been conducted in samples that are all or predominantly male and were sampled over 15 years ago [35–40]. Nyamathi et al. [41], reported 22% anti-HCV prevalence in a large sample (*n* = 884) of homeless adults in Los Angeles, yet the number of women in the sample and sex-specific prevalence were not specified. In a study of 387 clients seen at *Healthcare for the Homeless* clinics in eight cities, Strehlow et al. report that only 27.1% were women, and 21.9% of these anti-HCV positive, which is substantially lower than the prevalence reported here [42]. Whether the difference is due to differences in populations is unclear (e.g., a clinic population in the Strehlow study vs. a homeless population in the current study, a higher proportion of women in the current study, or geographic differences). In particular, the over-sampling of HIV infected women in this study may account for differences seen in the overall prevalence of anti-HCV in this compared to other studies, few of which included populations with HIV infection. However, the prevalence of anti-HCV among HIV-negative women participating in the current study was 37% (44 of 119 women), which is still higher than that reported in prior studies. We did not measure HCV RNA in this study, however based on other studies [43], including the National Health and Nutrition Examination Survey (NHANES) [10] showing that 75 to 82% of anti-HCV positive persons remain viremic, we estimate that 34.5 to 37.8% of women had chronic disease. This is potentially underestimated since some HIV-positive women may not have had detectable anti-HCV [44]. Due to their age and risk profile, there is a high probability that women in this study have been infected for decades, and thus many likely already have significant liver disease as well as other associated extra-hepatic comorbidities in addition to reduced quality of life [45, 46]. HIV/HCV coinfection among women in particular confers a significantly greater risk of disease compared to men suggesting that there is a sex differential in the role of immune suppression [47]. In a recent study (among a similarly aged population, median age 46 years), researchers found that HCV/HIV coinfected women with low CD4 counts had almost 10 times the risk of liver disease progression compared to monoinfected women, and coinfected men had 2.86 times the risk compared to monoinfected men. Results presented here, in combination with prior studies, suggest the possibility of unrecognized but substantial liver-related morbidity among homeless and unstably housed women. As with all studies and programs providing screening, referrals for further assessment for chronic infection and liver disease should be part of clinical follow up in this population.

Over two-thirds (72%) of women in this study were born before 1965, i.e., the ‘baby-boomer’ cohort. Not surprisingly, they, as well as those with a history of IDU, had significantly higher independent odds of HCV exposure compared to younger women and those with no IDU history [10, 39], reinforcing the current recommendations for HCV screening in these groups. Homeless women are a key medically and socially challenged population, who experience significant health disparities. While a substantive proportion of women in this sample were undiagnosed, including 30% of women in the baby-boomer cohort and 12.8% of women with a history of injecting, these proportions are lower than those seen in the general population where up to 50% of infected persons are not aware of infection status. In combination with the San Francisco Department of Public Health’s Homeless Outreach Team, the San Francisco Mayor’s Office has a dedicated HCV task force, and significant services targeting homeless populations (e.g., The Housing, Partnerships, Opportunity and Engagement Program [http://www.sfmayor.org/]), all of which may contribute to the higher testing rate. We observed that women who had lower income, fewer psychiatric diagnoses, and who had recently used cocaine were more likely to be unaware of their HCV status. These associations with income and psychiatric diagnoses suggest a need to expand HCV testing in homeless women overall, as both economic and psychiatric status may be tenuous. While associations between cocaine use and HCV are mixed [48] this exposure may be a proxy for higher risk exposures and thus patients who report this could benefit from HCV testing [49]. A large proportion of women overall report seeing a primary care provider recently, which also presents an opportunity for HCV screening. Finally, a higher proportion of HCV monoinfected women were unaware of their infection suggesting that those receiving HIV care were more likely to be tested for HCV. However, given that HCV screening in HIV-positive populations has been recommended since 2002 [50], it is surprising to find that more than a quarter (26.4%) of HIV/HCV coinfected women were unaware of their HCV status.

Unlike other studies we did not find associations between race/ethnicity, alcohol use, or sexual risk factors and HCV serostatus [41, 42], which may be due to less heterogeneity in risk within this population composed entirely of homeless and unstably housed women and oversampled for HIV. Our cut-offs for heavy and binge drinking may be overly conservative and could obscure higher levels of alcohol use. The positive association between number of psychiatric diagnoses and HCV exposure extends results from prior studies. For instance, Nyamathi et al. [41], reported elevated HCV among women with a history of hospitalization for mental illness. More recent reports underscore the high levels of HCV among persons with severe mental illness (17 vs. 1% in the general population) [51, 52]. Results presented here show for the first time that anti-HCV positivity increases additively with additional psychiatric conditions. As previously reported, the median number of co-occurring psychiatric conditions in this population is nine [22], suggesting odds of HCV exposure is elevated more than two-fold times higher in comparison with populations not impacted by psychiatric conditions. It is difficult to interpret the inverse association between depression and anti-HCV status, however this effect was independent of the number of psychiatric diagnoses, hinting that there are differential impacts by diagnosis. On a positive note, women with more psychiatric diagnoses in this study were more likely to be aware of their serostatus, potentially indicating that screening was incorporated into their clinical care.

The study has limitations which should be considered when interpreting results. The analyses are cross-sectional and thus temporal and causal effects cannot be inferred; however, confirming causation is not the goal of the study. Our overall intent is to inform testing and treatment programs by describing correlates of prevalent infection in a high risk population. While the analyses are secondary, and original data were gathered for other primary research questions, the SHADOW study did have secondary goals of documenting multiple health outcomes, of which HCV exposure was one. Only women who affirmed prior STI testing were specifically asked about HCV testing, and not all study participants would regard HCV as an infection included among STIs, thus the proportion of the entire population unaware of their anti-HCV status may be underestimated. In addition, sensitive self-reported data, such as injection drug use may have been underreported, however the effect of such would be to bias excess risk estimates toward the null, thus our results in this regard are likely conservative. Major strengths of the SHADOW study were its sampling methods, which recruited a group of women experiencing unstable housing that reflected San Francisco’s larger population of unstably housed women, and disparate health outcomes, including directly measured mental health conditions, thus our results are likely to have high external validity.

## Conclusions

The recent availability of simple, safe and curative treatment for HCV offers an enormous opportunity for improving health of millions of people, especially those experiencing homelessness, a population that experiences disproportionate impact of HCV. In 2014, surveys using ‘point-in-time’ counts found 564,708 to 578,424 homeless adults in the U.S., and 40% are women [53, 54]. Almost half of women participating in the current study had antibodies to HCV (anti-HCV) and approximately one quarter were unaware of their status. At a population level, the effect curative treatment could have on this population could be substantial. As a first step to advancing this goal, screening is critical. Increased risk-based testing and linkage to care, particularly among baby boomers and women with co-occurring mental health conditions, would benefit homeless women. Interventions to increase testing in high risk groups including targeted case finding, support and training for primary care practitioners, and offering dried blood spot testing have been shown to increase testing uptake [55]. Other approaches including electronic health record best practice alert and/or physician office based direct patient-solicitation could also effectively promote HCV testing in this group [56]. The presence of anti-HCV is correlated with an increasing number of psychiatric conditions in this population, and augmenting mental health care and support during HCV treatment may increase successful treatment in homeless and unstably housed women.

## Additional file

Additional file 1: SHADOW study interview. (PDF 531 kb)

## Abbreviations

AOR: Adjusted odds ratio
CDC: Centers for Disease Control and Prevention
CI: Confidence interval
DAA: Direct acting antivirals
EIA: Enzyme immunoassay
HCV: Hepatitis C virus
IDU: Injection drug use
PWID: People who inject drugs
SHADOW: Shelter, Health and Drug Outcomes among Women
SRO: Single room occupancy
STI: Sexually transmitted infection

## References

[1] Lansky A, Finlayson T, Johnson C, Holtzman D, Wejnert C, Mitsch A, Gust D, Chen R, Mizuno Y, Crepaz N (2014). Estimating the number of persons who inject drugs in the united states by meta-analysis to calculate national rates of HIV and hepatitis C virus infections. PLoS One.

[2] Page K, Hahn JA, Evans J, Shiboski S, Lum P, Delwart E, Tobler L, Andrews W, Avanesyan L, Cooper S (2009). Acute hepatitis C virus infection in young adult injection drug users: a prospective study of incident infection, resolution, and reinfection. J Infect Dis.

[3] Tohme RA, Xing J, Liao Y, Holmberg SD (2013). Hepatitis C testing, infection, and linkage to care among racial and ethnic minorities in the United States, 2009-2010. Am J Public Health.

[4] Fleckenstein J (2004). Chronic hepatitis C in African Americans and other minority groups. Curr Gastroenterol Rep.

[5] Trooskin SB, Navarro VJ, Winn RJ, Axelrod DJ, McNeal AS, Velez M, Herrine SK, Rossi S (2007). Hepatitis C risk assessment, testing and referral for treatment in urban primary care: role of race and ethnicity. World J Gastroenterol.

[6] Backus LI, Belperio PS, Loomis TP, Yip GH, Mole LA (2013). Hepatitis C virus screening and prevalence among US veterans in Department of Veterans Affairs care. JAMA Intern Med.

[7] Larney S, Kopinski H, Beckwith CG, Zaller ND, Jarlais DD, Hagan H, Rich JD, van den Bergh BJ, Degenhardt L (2013). Incidence and prevalence of hepatitis C in prisons and other closed settings: results of a systematic review and meta-analysis. Hepatology.

[8] Fox RK, Currie S, Wright TL, Tobler LH, Dailey P, Phelps B, Moss N, Busch MP, Page Shafer K (2002). Hepatitis C virus infection among prisoners in the California State Correctional system. J Gen Intern Med.

[9] McGovern BH, Wurcel A, Kim AY, Schulze zur Wiesch J, Bica I, Zaman MT, Timm J, Walker BD, Lauer GM (2006). Acute hepatitis C virus infection in incarcerated injection drug users. Clin Infect Dis.

[10] Denniston MM, Jiles RB, Drobeniuc J, Klevens RM, Ward JW, McQuillan GM, Holmberg SD (2014). Chronic hepatitis C virus infection in the United States, National Health and Nutrition Examination Survey 2003 to 2010. Ann Intern Med.

[11] Page-Shafer KA, Cahoon-Young B, Klausner JD, Morrow S, Molitor F, Ruiz J, McFarland W (2002). Hepatitis C virus infection in young, low-income women: the role of sexually transmitted infection as a potential cofactor for HCV infection. Am J Public Health.

[12] Hall CS, Charlebois ED, Hahn JA, Moss AR, Bangsberg DR (2004). Hepatitis C virus infection in San Francisco’s HIV-infected urban poor. J Gen Intern Med.

[13] Schwarz KB, Garrett B, Alter MJ, Thompson D, Strathdee SA (2008). Seroprevalence of HCV infection in homeless Baltimore families. J Health Care Poor Underserved.

[14] Backus LI, Boothroyd D, Deyton LR (2005). HIV, hepatitis C and HIV/hepatitis C virus co-infection in vulnerable populations. AIDS.

[15] Edlin BR, Eckhardt BJ, Shu MA, Holmberg SD, Swan T (2015). Toward a more accurate estimate of the prevalence of hepatitis C in the United States. Hepatology.

[16] Edlin BR, Winkelstein ER (2014). Can hepatitis C be eradicated in the United States?. Antiviral Res.

[17] CDC (1998). Recommendations for prevention and control of hepatitis C virus (HCV) infection and HCV-related chronic disease. MMWR Recomm Rep.

[18] USPHS/IDSA: Guidelines for the prevention of opportunistic infections in persons infected with human immunodeficiency virus. US Public Health Service (USPHS) and Infectious Diseases Society of America (IDSA) MMWR Recomm Rep 1999; 48(RR-10): 1–59, 61–6 1999. 10499670

[19] Smith BD, Morgan RL, Beckett GA (2012). Recommendations for the identification of chronic hepatitis C virus infection among persons born during 1945–1965. MMWR Recomm Rep.

[20] U.S. Preventive Services Task Force. Screening for hepatitis C virus infection in adults. Available at: http://www.uspreventiveservicestaskforce.org/uspstf/uspshepc.htm. Accessed 31 Aug 2015. 2013.

[21] Ly KN, Xing J, Klevens RM, Jiles RB, Ward JW, Holmberg SD (2012). The increasing burden of mortality from viral hepatitis in the United States between 1999 and 2007. Ann Intern Med.

[22] Riley ED, Cohen J, Knight KR, Decker A, Marson K, Shumway M (2014). Recent violence in a community-based sample of homeless and unstably housed women with high levels of psychiatric comorbidity. Am J Public Health.

[23] Burnam MA, Koegel P (1988). Methodology for obtaining a representative sample of homeless persons: the Los Angeles Skid Row Study. Eval Rev.

[24] Riley ED, Moore K, Sorensen JL, Tulsky JP, Bangsberg DR, Neilands TB (2011). Basic subsistence needs and overall health among human immunodeficiency virus-infected homeless and unstably housed women. Am J Epidemiol.

[25] ORTHO®: Hepatitis C Virus Encoded Antigen (Recombinant c22-3, c200 and NS5) ORTHO® HCV Version 3.0 ELISA Test System Enzyme-Linked Immunosorbent Assay for the Detection of Antibody to Hepatitis C Virus (Anti-HCV) in Human Serum or Plasma. Package Insert. Revised March 2009. http://www.fda.gov/downloads/BiologicsBloodVaccines/BloodBloodProducts/ApprovedProducts/LicensedProductsBLAs/BloodDonorScreening/InfectiousDisease/UCM176421.pdf (Accessed 8 Jan 2017). 2009.

[26] Gelberg L, Gallagher TC, Andersen RM, Koegel P (1997). Competing priorities as a barrier to medical care among homeless adults in Los Angeles. Am J Public Health.

[27] Gielen AC, McDonnell KA, Wu AW, O’Campo P, Faden R (2001). Quality of life among women living with HIV: the importance violence, social support, and self care behaviors. Soc Sci Med.

[28] Humphreys K, Moos RH, Finney JW (1995). Two pathways out of drinking problems without professional treatment. Addict Behav.

[29] Alcoholism. NIoAAa: NIAAA council approves definition of binge drinking [PDF-1.62 MB]. NIAAA Newsletter 2004; No. 3, p. 3.

[30] Zimmerman M, Coryell W (1988). The validity of a self-report questionnaire for diagnosing major depressive disorder. Arch Gen Psychiatry.

[31] American Psychiatric Association (1994). Diagnostic and statistical manual of mental disorders.

[32] Riley ED, Sorensen JL, Moore K, Tulsky JP, Bangsberg DR, Neilands TB (2011). Riley et al. respond to “co-occurring health conditions and life challenges”. Am J Epidemiol.

[33] Hosmer DW, Lemeshow S (2000). Applied Logistic Regression.

[34] Mickey RM, Greenland S (1989). The impact of confounder selection criteria on effect estimation. Am J Epidemiol.

[35] Desai RA, Rosenheck RA, Agnello V (2003). Prevalence of Hepatitis C virus infection in a sample of homeless veterans. Soc Psychiatry Psychiatr Epidemiol.

[36] Nyamathi AM, Dixon EL, Wiley D, Christiani A, Lowe A (2006). Hepatitis C virus infection among homeless men referred from a community clinic. West J Nurs Res.

[37] Cheung RC, Hanson AK, Maganti K, Keeffe EB, Matsui SM (2002). Viral hepatitis and other infectious diseases in a homeless population. J Clin Gastroenterol.

[38] Stein JA, Nyamathi A (2004). Correlates of hepatitis C virus infection in homeless men: a latent variable approach. Drug Alcohol Depend.

[39] Gelberg L, Robertson MJ, Arangua L, Leake BD, Sumner G, Moe A, Andersen RM, Morgenstern H, Nyamathi A (2012). Prevalence, distribution, and correlates of hepatitis C virus infection among homeless adults in Los Angeles. Public Health Rep.

[40] Boyce DE, Tice AD, Ona FV, Akinaka KT, Lusk H (2009). Viral hepatitis in a homeless shelter in Hawai’i. Hawaii Med J.

[41] Nyamathi AM, Dixon EL, Robbins W, Smith C, Wiley D, Leake B, Longshore D, Gelberg L (2002). Risk factors for hepatitis C virus infection among homeless adults. J Gen Intern Med.

[42] Strehlow AJ, Robertson MJ, Zerger S, Rongey C, Arangua L, Farrell E, O’Sullivan A, Gelberg L (2012). Hepatitis C among clients of health care for the homeless primary care clinics. J Health Care Poor Underserved.

[43] Grebely J, Page K, Sacks-Davis R, van der Loeff MS, Rice TM, Bruneau J, Morris MD, Hajarizadeh B, Amin J, Cox AL (2014). The effects of female sex, viral genotype, and IL28B genotype on spontaneous clearance of acute hepatitis C virus infection. Hepatology.

[44] George SL, Gebhardt J, Klinzman D, Foster MB, Patrick KD, Schmidt WN, Alden B, Pfaller MA, Stapleton JT (2002). Hepatitis C virus viremia in HIV-infected individuals with negative HCV antibody tests. J Acquir Immune Defic Syndr.

[45] Lingala S, Ghany MG (2015). Natural history of hepatitis C. Gastroenterol Clin North Am.

[46] Younossi Z, Park H, Adeyemi A, Stepanova M. Extrahepatic Manifestations of Hepatitis C: A Meta-analysis of Prevalence, Quality of Life, and Economic Burden.Gastroenterology. 2016;150(7):1599–608. doi:10.1053/j.gastro.2016.02.039. Epub 2016 Feb 26. 10.1053/j.gastro.2016.02.039 26924097

[47] Baranoski AS, Cotton D, Heeren T, Nunes D, Kubiak RW, Horsburgh CR (2016). Clinical liver disease progression among hepatitis C-infected drug users with CD4 cell count less than 200 cells/mm(3) is more pronounced among women than men. Open Forum Infect Dis.

[48] Scheinmann R, Hagan H, Lelutiu-Weinberger C, Stern R, Des Jarlais DC, Flom PL, Strauss S (2007). Non-injection drug use and Hepatitis C Virus: a systematic review. Drug Alcohol Depend.

[49] Artenie AA, Jutras-Aswad D, Roy E, Zang G, Bamvita JM, Levesque A, Bruneau J (2015). Visits to primary care physicians among persons who inject drugs at high risk of hepatitis C virus infection: room for improvement. J Viral Hepat.

[50] Kaplan JE, Masur H, Holmes KK, Usphs, Infectious Disease Society of A (2002). Guidelines for preventing opportunistic infections among HIV-infected persons--2002. Recommendations of the U.S. Public Health Service and the Infectious Diseases Society of America. MMWR Recomm Rep.

[51] Hughes E, Bassi S, Gilbody S, Bland M, Martin F (2016). Prevalence of HIV, hepatitis B, and hepatitis C in people with severe mental illness: a systematic review and meta-analysis. Lancet Psychiatry.

[52] Trager E, Khalili M, Masson CL, Vittinghoff E, Creasman J, Mangurian C (2016). Hepatitis C screening rate among underserved adults with serious mental illness receiving care in California Community Mental Health Centers. Am J Public Health.

[53] Henry M, Shivji A, de Sousa T, Cohen RaAAI: The 2015 Annual Homeless Assessment Report (AHAR) to Congress..US Department of Housing and Urban Development; Office of Community Planning and Development November 2015 https://www.hudexchange.info/resources/documents/2015-AHAR-Part-1.pdf Accessed 1 Apr 2016. 2015.

[54] National Alliance to End Homelessness. The State of Homelessness in America 2015 http://www.endhomelessness.org/page/-/files/State_of_Homelessness_2015_FINAL_online.pdf Accessed 1 Apr 2016

[55] Jones L, Bates G, McCoy E, Beynon C, McVeigh J, Bellis MA (2014). Effectiveness of interventions to increase hepatitis C testing uptake among high-risk groups: a systematic review. Eur J Public Health.

[56] Brady JE, Liffmann DK, Yartel A, Kil N, Federman AD, Kannry J, Jordan C, Massoud OI, Nerenz DR, Brown KA (2017). Uptake of hepatitis C screening, characteristics of patients tested, and intervention costs in the BEST-C study. Hepatology.

