# Supplementary material for: HCV screening in a cohort of HIV infected and uninfected homeless and marginally housed women in San Francisco, California
Source: BMC Public Health. 2017 Feb 7;17:171. doi: 10.1186/s12889-017-4102-5 (PMC5297184; doi:10.1186/s12889-017-4102-5)
Supplement: Additional file 1: — SHADOW study interview. (PDF 531 kb) [file 12889_2017_4102_MOESM1_ESM.pdf]

SHADOW 24mo (English)

Q1.	*[Enter participant ID.]*	__ __ __ __ __ __	
Q2.	*[Re-enter participant ID.]*	__ __ __ __ __ __	

If Q1 is not equal to Q2 then Subject IDs don't match. Please review. and skip to Q1.

If COUNTC1 is not equal to 0, then skip to instruction before Q3.

[BROKENC1]

If COUNTC1 is equal to 0, then skip to end of questionnaire.

If COUNT4 is not equal to 0, then skip to instruction before Q6.

Q3.	*[[BROKEN4A] Did PID [Response to Q1] miss her 18 month interview?]*	1	Yes
		0	No

If Q3 is equal to 1, then skip to instruction before Q4.

[BROKEN2A]

If COUNT4 is equal to 0, then skip to end of questionnaire.

If COUNT3 is not equal to 0, then skip to instruction before Q6.

Q4.	*[[BROKEN3] Did PID [Response to Q1] miss her 12 month interview?]*	1	Yes
		0	No

If Q4 is equal to 1, then skip to instruction before Q5.

[BROKEN2]

If COUNT3 is equal to 0, then skip to end of questionnaire.

If Q4 is not equal to 1, then skip to instruction before Q6.

If COUNT2 is not equal to 0, then skip to instruction before Q6.

Q5.	*[[BROKEN1] Did PID [Response to Q1] miss her 6 month interview?]*	1	Yes
		0	No

If Q5 is equal to 1, then skip to instruction before Q6.

If COUNT2 is not equal to 0, then skip to instruction before Q6.

[BROKEN4]

If COUNT2 is equal to 0, then skip to end of questionnaire.

If COUNT11 is not equal to 0, then skip to instruction before Q6.

[BROKEN11]

If COUNT11 is equal to 0, then skip to end of questionnaire.

Q6. *[Enter interviewer initials]* __

Q7. *[Enter date of last interview.]* __ __ / __ __ / __ __ __ __ mm / dd / yyyy

Q8. *[Interviewer enter participant's HIV status.]* (Choose one)

0	Negative
1	Positive
8	Refuse to Answer

Q9. *[Enter computer number.]* (Choose one)

1	Computer 1
2	Computer 2
3	Computer 3
4	Computer 4

Q10. *[Enter interview location.]* (Choose one)

1	Fieldsite
2	Phone
3	R's residence
4	Jail/prison
5	Hospital
6	Other

If Q10 is not equal to 6, then skip to instruction before DEM1.

Q11. *[Specify other interview location.]*

__ __
__ __ __ __ __ __ __ __ __ __

INTRODUCTION

Thank you for taking the time to talk with me today. We're going to be here for about an hour, so if you need to take a break, just let me know. There are no right or wrong answers, the questions are about your experiences and you're the only one who can know what that's like.

I just want to remind you that all of the information you give me is confidential. You do not have to answer any question that you do not want to answer, but I do ask that you be as honest as you can when you choose to answer.

We're going to start with me asking you questions. Then, we'll switch to you answering questions by yourself as the computer reads them to you. If you have any questions or would prefer to have me read the questions instead, just let me know.

Do you have any questions before we get started?

DEMOGRAPHICS

DEM1. What is your current marital status? (Choose one)

1 Single
2 Common-law married
3 Legally married
4 Separated/divorced
5 Widowed
8 Refuse to Answer

DEM2. What is your sexual orientation? (Choose one)

1 Heterosexual, you prefer to have sex with men
2 Homosexual/Lesbian, you prefer to have sex with women
3 Bisexual, you do not prefer sex with one over the other
8 Refuse to Answer

HOUSING/LIVING SITUATION

Now, I'm going to read a list of places where you may have stayed.

HOU1. In the past 6 months, have you stayed in a homeless shelter (such as MSC-South, A Woman's Place, or Sanctuary)?

1 Yes
0 No
8 Refuse to Answer

If HOU1 is not equal to 1, then skip to instruction before HOU3.

HOU2. About how many nights, weeks, or months have you stayed in a homeless shelter (such as MSC-South, A Woman's Place, or Sanctuary) in the past 6 months?

__ MONTHS
__ WEEKS
__ DAYS
8 Refuse to Answer (Months)

If HOU2 is greater than 6 then Number is too big. Please review. and skip to HOU2.

HOU3. In the past 6 months, have you stayed in a battered women's shelter, such as Casa de las Madres, or a crisis center?

1 Yes
0 No
8 Refuse to Answer

If HOU3 is not equal to 1, then skip to instruction before HOU5.

HOU4. About how many nights, weeks, or months have you stayed in a battered women's shelter, such as Casa de las Madres, or a crisis center in the past 6 months?

__ MONTHS
__ WEEKS
__ DAYS
8 Refuse to Answer (Months)

If HOU4 is greater than 6 then Number is too big. Please review. and skip to HOU4.

HOU5. In the past 6 months, have you stayed on the street, in an alleyway, in a park, or outdoor place?

1 Yes
0 No
8 Refuse to Answer

If HOU5 is not equal to 1, then skip to instruction before HOU7.

HOU6. About how many nights, weeks, or months have you stayed on the street, in an alleyway, in a park, or outdoor place in the past 6 months?

__ MONTHS
__ WEEKS
__ DAYS
8 Refuse to Answer (Months)

If HOU6 is greater than 6 then Number is too big. Please review. and skip to HOU6.

HOU7. In an abandoned building, warehouse, stairwell or other indoor place?

1 Yes
0 No
8 Refuse to Answer

If HOU7 is not equal to 1, then skip to instruction before HOU9.

HOU8. About how many nights, weeks, or months have you stayed in an abandoned building, warehouse, stairwell or other indoor place in the past 6 months?

__ MONTHS
__ WEEKS
__ DAYS
8 Refuse to Answer (Months)

If HOU8 is greater than 6 then Number is too big. Please review. and skip to HOU8.

HOU9. In a car, truck, or van?

1 Yes
0 No
8 Refuse to Answer

If HOU9 is not equal to 1, then skip to instruction before HOU11.

HOU10. About how many nights, weeks, or months have you stayed in a car, truck, or van in the past 6 months?

__ MONTHS
__ WEEKS
__ DAYS
8 Refuse to Answer (Months)

If HOU10 is greater than 6 then Number is too big. Please review. and skip to HOU10.

HOU11. In the past 6 months, have you stayed in your own SRO, weekly, or monthly hotel room?

1 Yes
0 No
8 Refuse to Answer

If HOU11 is not equal to 1, then skip to HOU110.

HOU12. How many different daily, weekly or monthly hotels have you stayed in during the past 6-months?

__ __ __ SRO rooms
998 Refuse to Answer

HOU13. What are the names of the hotels where you stayed?

[Note: Numbers through A Screen. Click GO TO NEXT SCREEN for B through C.] (Check all that apply)

- __ 16th Street Hotel
- __ Adrian Hotel
- __ Alcain Hotel
- __ Alder Hotel
- __ Alexander
- __ All Star Hotel
- __ Ambassador Hotel
- __ Apollo Hotel
- __ Aranda Hotel
- __ Arlington
- __ Artmar Hotel
- __ Auburn Hotel
- __ *** GO TO NEXT SCREEN ***
- __ Refuse to Answer

If HOU13Z is not equal to 1, then skip to instruction before HOU19.

HOU14. What are the names of the hotels where you stayed?

[Note: B through C Screen. Click GO TO NEXT SCREEN for D through H.] (Check all that apply)

- __ Baldwin Hotel
- __ Balmoral South
- __ Bayanihan
- __ Boston Hotel
- __ Boyd Hotel
- __ Bristol Hotel
- __ Broderick Residential Care
- __ CalDrake
- __ Cambridge
- __ Camelot Hotel
- __ Canon Kip
- __ CCR
- __ Civic Center Hotel
- __ Coast
- __ Coronado Hotel
- __ Covered Wagon
- __ CR Hotels
- __ Crown Hotel
- __ *** GO TO NEXT SCREEN ***
- __ Refuse to Answer

If HOU14Z is not equal to 1, then skip to instruction before HOU19.

HOU15. What are the names of the hotels where you stayed?

[Note: D through H Screen. Click GO TO NEXT SCREEN for J through N.] (Check all that apply)

__ Dahlia Hotel
__ Dalt
__ Desmond
__ Drake Hotel
__ Dudley
__ Elk
__ Elis Hotel
__ Elm Hotel
__ Empress Hotel
__ Folsom/Dore Hotel
__ Grand Southern Hotel
__ Graystone
__ Hamlin
__ Hartland Hotel
__ Henry Hotel
__ Hillsdale
__ Hotel Union
__ *** GO TO NEXT SCREEN ***
__ Refuse to Answer

If HOU15Z is not equal to 1, then skip to instruction before HOU19.

HOU16. What are the names of the hotels where you stayed?

[Note: J through N Screen. Click GO TO NEXT SCREEN for O through R.] (Check all that apply)

__ Jefferson
__ Kean Hotel
__ Kinney Hotel
__ Knox
__ Le Nain Hotel
__ Leroy Looper
__ Lifeboat Lodge Hotel
__ Lyric
__ Mary Elizabeth Inn
__ McAllister Hotel
__ Mentone
__ Minna Lee Hotel
__ Mission Creek Apartments
__ Mission Hotel
__ National Hotel
__ Neil
__ *** GO TO NEXT SCREEN ***
__ Refuse to Answer

If HOU16Z is not equal to 1, then skip to instruction before HOU19.

HOU17. What are the names of the hotels where you stayed?

[Note: O through R Screen. Click GO TO NEXT SCREEN for S through W.] (Check all that apply)

__ Oakwood Hotel
__ Orlando Hotel
__ Pacific Bay Inn
__ Pierre
__ Plaza Hotel
__ Prita
__ Rahda
__ Raymond Hotel
__ Ritz
__ Riviera Hotel
__ Rose
__ Royan
__ *** GO TO NEXT SCREEN ***
__ Refuse to Answer

If HOU17Z is not equal to 1, then skip to instruction before HOU19.

HOU18. What are the names of the hotels where you stayed?

[Note: Sthrough W Screen.] (Check all that apply)

__	Seneca
__	Seneca Hotel
__	Star Hotel
__	Sunnyside Hotel
__	Vincent
__	Vincent Hotel
__	Warfield Hotel
__	West Hotel
__	William Penn
__	Windsor Hotel
__	Other #1 (specify)
__	Other #2 (specify)
__	Other #3 (specify)
__	Refuse to Answer

If SROLNUM is not equal to HOU12 then Earlier, you reported staying in &[HOU6NUM] of your own SRO hotels, but you've selected &[SROLNUM] ones from the list. Please review. and skip to HOU12.

If HOU13A is not equal to 1, then skip to instruction before HOU20.

HOU19. About how many nights, weeks, or months did you stay at the 16th Street Hotel in the past 6 months?

__	MONTHS
__	WEEKS
__	DAYS
8	Refuse to Answer (Months)

If HOU13B is not equal to 1, then skip to instruction before HOU21.

HOU20. About how many nights, weeks, or months did you stay at the Adrian Hotel in the past 6 months?

__	MONTHS
__	WEEKS
__	DAYS
8	Refuse to Answer (Months)

If HOU13C is not equal to 1, then skip to instruction before HOU22.

HOU21. About how many nights, weeks, or months did you stay at the Alcain Hotel in the past 6 months?

__	MONTHS
__	WEEKS
__	DAYS
8	Refuse to Answer (Months)

If HOU13D is not equal to 1, then skip to instruction before HOU23.

HOU22. About how many nights, weeks, or months did you stay at the Alder Hotel in the past 6 months?

__	MONTHS
__	WEEKS
__	DAYS
8	Refuse to Answer (Months)

If HOU13E is not equal to 1, then skip to instruction before HOU24.

HOU23. About how many nights, weeks, or months did you stay at the Alexander in the past 6 months?

__	MONTHS
__	WEEKS
__	DAYS
8	Refuse to Answer (Months)

If HOU13F is not equal to 1, then skip to instruction before HOU25.

HOU24. About how many nights, weeks, or months did you stay at the All Star Hotel in the past 6 months?

__	MONTHS
__	WEEKS
__	DAYS
8	Refuse to Answer (Months)

If HOU13G is not equal to 1, then skip to instruction before HOU26.

HOU25. About how many nights, weeks, or months did you stay at the Ambassador Hotel in the past 6 months?

__	MONTHS
__	WEEKS
__	DAYS
8	Refuse to Answer (Months)

If HOU13H is not equal to 1, then skip to instruction before HOU27.

HOU26. About how many nights, weeks, or months did you stay at the Apollo Hotel in the past 6 months?

__	MONTHS
__	WEEKS
__	DAYS
8	Refuse to Answer (Months)

If HOU13I is not equal to 1, then skip to instruction before HOU28.

HOU27. About how many nights, weeks, or months did you stay at the Aranda Hotel in the past 6 months?

__	MONTHS
__	WEEKS
__	DAYS
8	Refuse to Answer (Months)

If HOU13J is not equal to 1, then skip to instruction before HOU29.

HOU28. About how many nights, weeks, or months did you stay at the Arlington in the past 6 months?

__	MONTHS
__	WEEKS
__	DAYS
8	Refuse to Answer (Months)

If HOU13K is not equal to 1, then skip to instruction before HOU30.

HOU29. About how many nights, weeks, or months did you stay at the Artmar Hotel in the past 6 months?

__	MONTHS
__	WEEKS
__	DAYS
8	Refuse to Answer (Months)

If HOU13L is not equal to 1, then skip to instruction before HOU31.

HOU30. About how many nights, weeks, or months did you stay at the Auburn Hotel in the past 6 months?

__	MONTHS
__	WEEKS
__	DAYS
8	Refuse to Answer (Months)

If HOU14A is not equal to 1, then skip to instruction before HOU32.

HOU31. About how many nights, weeks, or months did you stay at the Baldwin Hotel in the past 6 months?

__	MONTHS
__	WEEKS
__	DAYS
8	Refuse to Answer (Months)

If HOU14B is not equal to 1, then skip to instruction before HOU33.

HOU32. About how many nights, weeks, or months did you stay at the Balmoral South Hotel in the past 6 months?

__	MONTHS
__	WEEKS
__	DAYS
8	Refuse to Answer (Months)

If HOU14C is not equal to 1, then skip to instruction before HOU34.

HOU33. About how many nights, weeks, or months did you stay at the Bayanihan in the past 6 months?

__	MONTHS
__	WEEKS
__	DAYS
8	Refuse to Answer (Months)

If HOU14D is not equal to 1, then skip to instruction before HOU35.

HOU34. About how many nights, weeks, or months did you stay at the Boston Hotel in the past 6 months?

__	MONTHS
__	WEEKS
__	DAYS
8	Refuse to Answer (Months)

If HOU14E is not equal to 1, then skip to instruction before HOU36.

HOU35. About how many nights, weeks, or months did you stay at the Boyd Hotel in the past 6 months?

__	MONTHS
__	WEEKS
__	DAYS
8	Refuse to Answer (Months)

If HOU14F is not equal to 1, then skip to instruction before HOU37.

HOU36. About how many nights, weeks, or months did you stay at the Bristol Hotel in the past 6 months?

__	MONTHS
__	WEEKS
__	DAYS
8	Refuse to Answer (Months)

If HOU14G is not equal to 1, then skip to instruction before HOU38.

HOU37. About how many nights, weeks, or months did you stay at the Broderick Residential Care Hotel in the past 6 months?

__	MONTHS
__	WEEKS
__	DAYS
8	Refuse to Answer (Months)

If HOU14H is not equal to 1, then skip to instruction before HOU39.

HOU38. About how many nights, weeks, or months did you stay at the CalDrake in the past 6 months?

__	MONTHS
__	WEEKS
__	DAYS
8	Refuse to Answer (Months)

If HOU14I is not equal to 1, then skip to instruction before HOU40.

HOU39. About how many nights, weeks, or months did you stay at the Cambridge in the past 6 months?

__	MONTHS
__	WEEKS
__	DAYS
8	Refuse to Answer (Months)

If HOU14J is not equal to 1, then skip to instruction before HOU41.

HOU40. About how many nights, weeks, or months did you stay at the Camelot Hotel in the past 6 months?

__	MONTHS
__	WEEKS
__	DAYS
8	Refuse to Answer (Months)

If HOU14K is not equal to 1, then skip to instruction before HOU42.

HOU41. About how many nights, weeks, or months did you stay at the Canon Kip in the past 6 months?

__	MONTHS
__	WEEKS
__	DAYS
8	Refuse to Answer (Months)

If HOU14L is not equal to 1, then skip to instruction before HOU43.

HOU42. About how many nights, weeks, or months did you stay at the CCR in the past 6 months?

__	MONTHS
__	WEEKS
__	DAYS
8	Refuse to Answer (Months)

If HOU14M is not equal to 1, then skip to instruction before HOU44.

HOU43. About how many nights, weeks, or months did you stay at the Civic Center Hotel in the past 6 months?

__	MONTHS
__	WEEKS
__	DAYS
8	Refuse to Answer (Months)

If HOU14N is not equal to 1, then skip to instruction before HOU45.

HOU44. About how many nights, weeks, or months did you stay at the Coast Hotel in the past 6 months?

__	MONTHS
__	WEEKS
__	DAYS
8	Refuse to Answer (Months)

If HOU14O is not equal to 1, then skip to instruction before HOU46.

HOU45. About how many nights, weeks, or months did you stay at the Coronado Hotel in the past 6 months?

__	MONTHS
__	WEEKS
__	DAYS
8	Refuse to Answer (Months)

If HOU14P is not equal to 1, then skip to instruction before HOU47.

HOU46. About how many nights, weeks, or months did you stay at the Covered Wagon in the past 6 months?

__	MONTHS
__	WEEKS
__	DAYS
8	Refuse to Answer (Months)

If HOU14Q is not equal to 1, then skip to instruction before HOU48.

HOU47. About how many nights, weeks, or months did you stay at the CR Hotels in the past 6 months?

__	MONTHS
__	WEEKS
__	DAYS
8	Refuse to Answer (Months)

If HOU14R is not equal to 1, then skip to instruction before HOU49.

HOU48. About how many nights, weeks, or months did you stay at the Crown Hotel in the past 6 months?

__	MONTHS
__	WEEKS
__	DAYS
8	Refuse to Answer (Months)

If HOU15A is not equal to 1, then skip to instruction before HOU50.

HOU49. About how many nights, weeks, or months did you stay at the Dahlia Hotel in the past 6 months?

__	MONTHS
__	WEEKS
__	DAYS
8	Refuse to Answer (Months)

If HOU15B is not equal to 1, then skip to instruction before HOU51.

HOU50. About how many nights, weeks, or months did you stay at the Dalt in the past 6 months?

__	MONTHS
__	WEEKS
__	DAYS
8	Refuse to Answer (Months)

If HOU15C is not equal to 1, then skip to instruction before HOU52.

HOU51. About how many nights, weeks, or months did you stay at the Desmond in the past 6 months?

__	MONTHS
__	WEEKS
__	DAYS
8	Refuse to Answer (Months)

If HOU15D is not equal to 1, then skip to instruction before HOU53.

HOU52. About how many nights, weeks, or months did you stay at the Drake Hotel in the past 6 months?

__	MONTHS
__	WEEKS
__	DAYS
8	Refuse to Answer (Months)

If HOU15E is not equal to 1, then skip to instruction before HOU54.

HOU53. About how many nights, weeks, or months did you stay at the Dudley in the past 6 months?

__	MONTHS
__	WEEKS
__	DAYS
8	Refuse to Answer (Months)

If HOU15F is not equal to 1, then skip to instruction before HOU55.

HOU54. About how many nights, weeks, or months did you stay at the Elk in the past 6 months?

__	MONTHS
__	WEEKS
__	DAYS
8	Refuse to Answer (Months)

If HOU15G is not equal to 1, then skip to instruction before HOU56.

HOU55. About how many nights, weeks, or months did you stay at the Ellis Hotel in the past 6 months?

__	MONTHS
__	WEEKS
__	DAYS
8	Refuse to Answer (Months)

If HOU15H is not equal to 1, then skip to instruction before HOU57.

HOU56. About how many nights, weeks, or months did you stay at the Elm Hotel in the past 6 months?

__	MONTHS
__	WEEKS
__	DAYS
8	Refuse to Answer (Months)

If HOU15I is not equal to 1, then skip to instruction before HOU58.

HOU57. About how many nights, weeks, or months did you stay at the Empress Hotel in the past 6 months?

__	MONTHS
__	WEEKS
__	DAYS
8	Refuse to Answer (Months)

If HOU15J is not equal to 1, then skip to instruction before HOU59.

HOU58. About how many nights, weeks, or months did you stay at the Folsom/Dore Hotel in the past 6 months?

__	MONTHS
__	WEEKS
__	DAYS
8	Refuse to Answer (Months)

If HOU15K is not equal to 1, then skip to instruction before HOU60.

HOU59. About how many nights, weeks, or months did you stay at the Grand Southern Hotel in the past 6 months?

__	MONTHS
__	WEEKS
__	DAYS
8	Refuse to Answer (Months)

If HOU15L is not equal to 1, then skip to instruction before HOU61.

HOU60. About how many nights, weeks, or months did you stay at the Graystone in the past 6 months?

__	MONTHS
__	WEEKS
__	DAYS
8	Refuse to Answer (Months)

If HOU15M is not equal to 1, then skip to instruction before HOU62.

HOU61. About how many nights, weeks, or months did you stay at the Hamlin in the past 6 months?

__	MONTHS
__	WEEKS
__	DAYS
8	Refuse to Answer (Months)

If HOU15N is not equal to 1, then skip to instruction before HOU63.

HOU62. About how many nights, weeks, or months did you stay at the Hartland Hotel in the past 6 months?

__	MONTHS
__	WEEKS
__	DAYS
8	Refuse to Answer (Months)

If HOU15O is not equal to 1, then skip to instruction before HOU64.

HOU63. About how many nights, weeks, or months did you stay at the Henry Hotel in the past 6 months?

__	MONTHS
__	WEEKS
__	DAYS
8	Refuse to Answer (Months)

If HOU15P is not equal to 1, then skip to instruction before HOU65.

HOU64. About how many nights, weeks, or months did you stay at the Hillsdale in the past 6 months?

__	MONTHS
__	WEEKS
__	DAYS
8	Refuse to Answer (Months)

If HOU15Q is not equal to 1, then skip to instruction before HOU66.

HOU65. About how many nights, weeks, or months did you stay at the Hotel Union in the past 6 months?

__	MONTHS
__	WEEKS
__	DAYS
8	Refuse to Answer (Months)

If HOU16A is not equal to 1, then skip to instruction before HOU67.

HOU66. About how many nights, weeks, or months did you stay at the Jefferson in the past 6 months?

__	MONTHS
__	WEEKS
__	DAYS
8	Refuse to Answer (Months)

If HOU16B is not equal to 1, then skip to instruction before HOU68.

HOU67. About how many nights, weeks, or months did you stay at the Kean Hotel in the past 6 months?

__	MONTHS
__	WEEKS
__	DAYS
8	Refuse to Answer (Months)

If HOU16C is not equal to 1, then skip to instruction before HOU69.

HOU68. About how many nights, weeks, or months did you stay at the Kinney Hotel in the past 6 months?

__	MONTHS
__	WEEKS
__	DAYS
8	Refuse to Answer (Months)

If HOU16D is not equal to 1, then skip to instruction before HOU70.

HOU69. About how many nights, weeks, or months did you stay at the Knox in the past 6 months?

__	MONTHS
__	WEEKS
__	DAYS
8	Refuse to Answer (Months)

If HOU16E is not equal to 1, then skip to instruction before HOU71.

HOU70. About how many nights, weeks, or months did you stay at the Le Nain Hotel in the past 6 months?

__	MONTHS
__	WEEKS
__	DAYS
8	Refuse to Answer (Months)

If HOU16F is not equal to 1, then skip to instruction before HOU72.

HOU71. About how many nights, weeks, or months did you stay at the Leroy Looper in the past 6 months?

__	MONTHS
__	WEEKS
__	DAYS
8	Refuse to Answer (Months)

If HOU16G is not equal to 1, then skip to instruction before HOU73.

HOU72. About how many nights, weeks, or months did you stay at the Lifeboat Lodge Hotel in the past 6 months?

__	MONTHS
__	WEEKS
__	DAYS
8	Refuse to Answer (Months)

If HOU16H is not equal to 1, then skip to instruction before HOU74.

HOU73. About how many nights, weeks, or months did you stay at the Lyric in the past 6 months?

__	MONTHS
__	WEEKS
__	DAYS
8	Refuse to Answer (Months)

If HOU16I is not equal to 1, then skip to instruction before HOU75.

HOU74. About how many nights, weeks, or months did you stay at the Mary Elizabeth Inn in the past 6 months?

__	MONTHS
__	WEEKS
__	DAYS
8	Refuse to Answer (Months)

If HOU16J is not equal to 1, then skip to instruction before HOU76.

HOU75. About how many nights, weeks, or months did you stay at the McAllister Hotel in the past 6 months?

__	MONTHS
__	WEEKS
__	DAYS
8	Refuse to Answer (Months)

If HOU16K is not equal to 1, then skip to instruction before HOU77.

HOU76. About how many nights, weeks, or months did you stay at the Mentone in the past 6 months?

__	MONTHS
__	WEEKS
__	DAYS
8	Refuse to Answer (Months)

If HOU16L is not equal to 1, then skip to instruction before HOU78.

HOU77. About how many nights, weeks, or months did you stay at the Minna Lee Hotel in the past 6 months?

__	MONTHS
__	WEEKS
__	DAYS
8	Refuse to Answer (Months)

If HOU16M is not equal to 1, then skip to instruction before HOU79.

HOU78. About how many nights, weeks, or months did you stay at the Mission Creek Apartments in the past 6 months?

__	MONTHS
__	WEEKS
__	DAYS
8	Refuse to Answer (Months)

If HOU16N is not equal to 1, then skip to instruction before HOU80.

HOU79. About how many nights, weeks, or months did you stay at the Mission Hotel in the past 6 months?

__	MONTHS
__	WEEKS
__	DAYS
8	Refuse to Answer (Months)

If HOU16O is not equal to 1, then skip to instruction before HOU81.

HOU80. About how many nights, weeks, or months did you stay at the National Hotel in the past 6 months?

__	MONTHS
__	WEEKS
__	DAYS
8	Refuse to Answer (Months)

If HOU16P is not equal to 1, then skip to instruction before HOU82.

HOU81. About how many nights, weeks, or months did you stay at the Neil in the past 6 months?

__	MONTHS
__	WEEKS
__	DAYS
8	Refuse to Answer (Months)

If HOU17A is not equal to 1, then skip to instruction before HOU83.

HOU82. About how many nights, weeks, or months did you stay at the Oakwood Hotel in the past 6 months?

__	MONTHS
__	WEEKS
__	DAYS
8	Refuse to Answer (Months)

If HOU17B is not equal to 1, then skip to instruction before HOU84.

HOU83. About how many nights, weeks, or months did you stay at the Orlando Hotel in the past 6 months?

__	MONTHS
__	WEEKS
__	DAYS
8	Refuse to Answer (Months)

If HOU17D is not equal to 1, then skip to instruction before HOU85.

HOU84. About how many nights, weeks, or months did you stay at the Pacific Bay Inn in the past 6 months?

__	MONTHS
__	WEEKS
__	DAYS
8	Refuse to Answer (Months)

If HOU17E is not equal to 1, then skip to instruction before HOU86.

HOU85. About how many nights, weeks, or months did you stay at the Pierre in the past 6 months?

__	MONTHS
__	WEEKS
__	DAYS
8	Refuse to Answer (Months)

If HOU17F is not equal to 1, then skip to instruction before HOU87.

HOU86. About how many nights, weeks, or months did you stay at the Plaza Hotel in the past 6 months?

__	MONTHS
__	WEEKS
__	DAYS
8	Refuse to Answer (Months)

If HOU17G is not equal to 1, then skip to instruction before HOU88.

HOU87. About how many nights, weeks, or months did you stay at the Prita in the past 6 months?

__	MONTHS
__	WEEKS
__	DAYS
8	Refuse to Answer (Months)

If HOU17H is not equal to 1, then skip to instruction before HOU89.

HOU88. About how many nights, weeks, or months did you stay at the Rahda in the past 6 months?

__	MONTHS
__	WEEKS
__	DAYS
8	Refuse to Answer (Months)

If HOU17I is not equal to 1, then skip to instruction before HOU90.

HOU89. About how many nights, weeks, or months did you stay at the Raymond Hotel in the past 6 months?

__	MONTHS
__	WEEKS
__	DAYS
8	Refuse to Answer (Months)

If HOU17J is not equal to 1, then skip to instruction before HOU91.

HOU90. About how many nights, weeks, or months did you stay at the Ritz in the past 6 months?

__	MONTHS
__	WEEKS
__	DAYS
8	Refuse to Answer (Months)

If HOU17K is not equal to 1, then skip to instruction before HOU92.

HOU91. About how many nights, weeks, or months did you stay at the Riviera Hotel in the past 6 months?

__	MONTHS
__	WEEKS
__	DAYS
8	Refuse to Answer (Months)

If HOU17L is not equal to 1, then skip to instruction before HOU93.

HOU92. About how many nights, weeks, or months did you stay at the Rose in the past 6 months?

__	MONTHS
__	WEEKS
__	DAYS
8	Refuse to Answer (Months)

If HOU17M is not equal to 1, then skip to instruction before HOU94.

HOU93. About how many nights, weeks, or months did you stay at the Royan in the past 6 months?

__	MONTHS
__	WEEKS
__	DAYS
8	Refuse to Answer (Months)

If HOU18A is not equal to 1, then skip to instruction before HOU95.

HOU94. About how many nights, weeks, or months did you stay at the Seneca in the past 6 months?

__	MONTHS
__	WEEKS
__	DAYS
8	Refuse to Answer (Months)

If HOU18B is not equal to 1, then skip to instruction before HOU96.

HOU95. About how many nights, weeks, or months did you stay at the Seneca Hotel in the past 6 months?

__	MONTHS
__	WEEKS
__	DAYS
8	Refuse to Answer (Months)

If HOU18C is not equal to 1, then skip to instruction before HOU97.

HOU96. About how many nights, weeks, or months did you stay at the Star Hotel in the past 6 months?

__	MONTHS
__	WEEKS
__	DAYS
8	Refuse to Answer (Months)

If HOU18D is not equal to 1, then skip to instruction before HOU98.

HOU97. About how many nights, weeks, or months did you stay at the Sunnyside Hotel in the past 6 months?

__	MONTHS
__	WEEKS
__	DAYS
8	Refuse to Answer (Months)

If HOU18E is not equal to 1, then skip to instruction before HOU99.

HOU98. About how many nights, weeks, or months did you stay at the Vincent in the past 6 months?

__	MONTHS
__	WEEKS
__	DAYS
8	Refuse to Answer (Months)

If HOU18F is not equal to 1, then skip to instruction before HOU100.

HOU99. About how many nights, weeks, or months did you stay at the Vincent Hotel in the past 6 months?

__	MONTHS
__	WEEKS
__	DAYS
8	Refuse to Answer (Months)

If HOU18G is not equal to 1, then skip to instruction before HOU101.

HOU100. About how many nights, weeks, or months did you stay at the Warfield Hotel in the past 6 months?

__	MONTHS
__	WEEKS
__	DAYS
8	Refuse to Answer (Months)

If HOU18H is not equal to 1, then skip to instruction before HOU102.

HOU101. About how many nights, weeks, or months did you stay at the West Hotel in the past 6 months?

__	MONTHS
__	WEEKS
__	DAYS
8	Refuse to Answer (Months)

If HOU18I is not equal to 1, then skip to instruction before HOU103.

HOU102. About how many nights, weeks, or months did you stay at the William Penn in the past 6 months?

__	MONTHS
__	WEEKS
__	DAYS
8	Refuse to Answer (Months)

If HOU18J is not equal to 1, then skip to instruction before HOU104.

HOU103. About how many nights, weeks, or months did you stay at the Windsor Hotel in the past 6 months?

__	MONTHS
__	WEEKS
__	DAYS
8	Refuse to Answer (Months)

If HOU18K is not equal to 1, then skip to HOU110.

HOU104. Please specify the name of the first "other" SRO.

— —
— — — — — — — — — —

HOU105. About how many nights, weeks, or months did you stay at the [Response to HOU104] in the past 6 months?

__	MONTHS
__	WEEKS
__	DAYS
8	Refuse to Answer (Months)

If HOU18L is not equal to 1, then skip to HOU110.

HOU106. Please specify the name of the second "other" SRO.

— —

HOU107. About how many nights, weeks, or months did you stay at the [Response to HOU106] in the past 6 months?

__	MONTHS
__	WEEKS
__	DAYS
8	Refuse to Answer (Months)

If HOU18M is not equal to 1, then skip to HOU110.

HOU108. Please specify the name of the third "other" SRO.

— —

HOU109. About how many nights, weeks, or months did you stay at the [Response to HOU108] in the past 6 months?

__	MONTHS
__	WEEKS
__	DAYS
8	Refuse to Answer (Months)

HOU110. In someone else's SRO, weekly or monthly hotel room?

1	Yes
0	No
8	Refuse to Answer

If HOU110 is not equal to 1, then skip to HOU209.

HOU111. How many different daily, weekly, or monthly hotels did you stay with someone in during the past 6-months?

__ __ __	SRO rooms
998	Refuse to Answer

HOU112. What are the names of the hotels where you stayed with someone else?

[Note: Numbers through A Screen. Click GO TO NEXT SCREEN for B through C.] (Check all that apply)

- __ 16th Street Hotel
- __ Adrian Hotel
- __ Alcain Hotel
- __ Alder Hotel
- __ Alexander
- __ All Star Hotel
- __ Ambassador Hotel
- __ Apollo Hotel
- __ Aranda Hotel
- __ Arlington
- __ Artmar Hotel
- __ Auburn Hotel
- __ *** GO TO NEXT SCREEN ***
- __ Refuse to Answer

If HOU112Z is not equal to 1, then skip to instruction before HOU118.

HOU113. What are the names of the hotels where you stayed with someone else?

[Note: B through C Screen. Click GO TO NEXT SCREEN for D through H.] (Check all that apply)

- __ Baldwin Hotel
- __ Balmoral South
- __ Bayanihan
- __ Boston Hotel
- __ Boyd Hotel
- __ Bristol Hotel
- __ Broderick Residential Care
- __ CalDrake
- __ Cambridge
- __ Camelot Hotel
- __ Canon Kip
- __ CCR
- __ Civic Center Hotel
- __ Coast
- __ Coronado Hotel
- __ Covered Wagon
- __ CR Hotels
- __ Crown Hotel
- __ *** GO TO NEXT SCREEN ***
- __ Refuse to Answer

If HOU113Z is not equal to 1, then skip to instruction before HOU118.

HOU114. What are the names of the hotels where you stayed with someone else?

[Note: D through H Screen. Click GO TO NEXT SCREEN for J through N.] (Check all that apply)

__ Dahlia Hotel
__ Dalt
__ Desmond
__ Drake Hotel
__ Dudley
__ Elk
__ Elis Hotel
__ Elm Hotel
__ Empress Hotel
__ Folsom/Dore Hotel
__ Grand Southern Hotel
__ Graystone
__ Hamlin
__ Hartland Hotel
__ Henry Hotel
__ Hillsdale
__ Hotel Union
__ *** GO TO NEXT SCREEN ***
__ Refuse to Answer

If HOU114Z is not equal to 1, then skip to instruction before HOU118.

HOU115. What are the names of the hotels where you stayed with someone else?

[Note: J through N Screen. Click GO TO NEXT SCREEN for O through R.] (Check all that apply)

- __ Jefferson
- __ Kean Hotel
- __ Kinney Hotel
- __ Knox
- __ Le Nain Hotel
- __ Leroy Looper
- __ Lifeboat Lodge Hotel
- __ Lyric
- __ Mary Elizabeth Inn
- __ McAllister Hotel
- __ Mentone
- __ Minna Lee Hotel
- __ Mission Creek Apartments
- __ Mission Hotel
- __ National Hotel
- __ Neil
- __ *** GO TO NEXT SCREEN ***
- __ Refuse to Answer

If HOU115Z is not equal to 1, then skip to instruction before HOU118.

HOU116. What are the names of the hotels where you stayed with someone else?

[Note: O through R Screen. Click GO TO NEXT SCREEN for S through W.] (Check all that apply)

- __ Oakwood Hotel
- __ Orlando Hotel
- __ Pacific Bay Inn
- __ Pierre
- __ Plaza Hotel
- __ Prita
- __ Rahda
- __ Raymond Hotel
- __ Ritz
- __ Riviera Hotel
- __ Rose
- __ Royan
- __ *** GO TO NEXT SCREEN ***
- __ Refuse to Answer

If HOU116Z is not equal to 1, then skip to instruction before HOU118.

HOU117. What are the names of the hotels where you stayed with someone else?

[Note: Sthrough W Screen.] (Check all that apply)

__	Seneca
__	Seneca Hotel
__	Star Hotel
__	Sunnyside Hotel
__	Vincent
__	Vincent Hotel
__	Warfield Hotel
__	West Hotel
__	William Penn
__	Windsor Hotel
__	Other 1 (specify)
__	Other 2 (specify)
__	Other 3 (specify)
__	Refuse to Answer

If SROMNUM is not equal to HOU111 then Earlier, you reported staying in &[HOU7NUM] SRO hotels with someone else, but you've selected &[SROmNUM] ones from the list. Please review. and skip to HOU111.

If HOU112A is not equal to 1, then skip to instruction before HOU119.

HOU118. About how many nights, weeks, or months did you stay at the 16th Street Hotel with someone else in the past 6 months?

__	MONTHS
__	WEEKS
__	DAYS
8	Refuse to Answer (Months)

If HOU112B is not equal to 1, then skip to instruction before HOU120.

HOU119. About how many nights, weeks, or months did you stay at the Adrian Hotel with someone else in the past 6 months?

__	MONTHS
__	WEEKS
__	DAYS
8	Refuse to Answer (Months)

If HOU112C is not equal to 1, then skip to instruction before HOU121.

HOU120. About how many nights, weeks, or months did you stay at the Alcain Hotel with someone else in the past 6 months?

__	MONTHS
__	WEEKS
__	DAYS
8	Refuse to Answer (Months)

If HOU112D is not equal to 1, then skip to instruction before HOU122.

HOU121. About how many nights, weeks, or months did you stay at the Alder Hotel with someone else in the past 6 months?

__	MONTHS
__	WEEKS
__	DAYS
8	Refuse to Answer (Months)

If HOU112E is not equal to 1, then skip to instruction before HOU123.

HOU122. About how many nights, weeks, or months did you stay at the Alexander with someone else in the past 6 months?

__	MONTHS
__	WEEKS
__	DAYS
8	Refuse to Answer (Months)

If HOU112F is not equal to 1, then skip to instruction before HOU124.

HOU123. About how many nights, weeks, or months did you stay at the All Star Hotel with someone else in the past 6 months?

__	MONTHS
__	WEEKS
__	DAYS
8	Refuse to Answer (Months)

If HOU112G is not equal to 1, then skip to instruction before HOU125.

HOU124. About how many nights, weeks, or months did you stay at the Ambassador Hotel with someone else in the past 6 months?

__	MONTHS
__	WEEKS
__	DAYS
8	Refuse to Answer (Months)

If HOU112H is not equal to 1, then skip to instruction before HOU126.

HOU125. About how many nights, weeks, or months did you stay at the Apollo Hotel with someone else in the past 6 months?

__	MONTHS
__	WEEKS
__	DAYS
8	Refuse to Answer (Months)

If HOU112I is not equal to 1, then skip to instruction before HOU127.

HOU126. About how many nights, weeks, or months did you stay at the Aranda Hotel with someone else in the past 6 months?

__	MONTHS
__	WEEKS
__	DAYS
8	Refuse to Answer (Months)

If HOU112J is not equal to 1, then skip to instruction before HOU128.

HOU127. About how many nights, weeks, or months did you stay at the Arlington with someone else in the past 6 months?

__	MONTHS
__	WEEKS
__	DAYS
8	Refuse to Answer (Months)

If HOU112K is not equal to 1, then skip to instruction before HOU129.

HOU128. About how many nights, weeks, or months did you stay at the Artmar Hotel with someone else in the past 6 months?

__	MONTHS
__	WEEKS
__	DAYS
8	Refuse to Answer (Months)

If HOU112L is not equal to 1, then skip to instruction before HOU130.

HOU129. About how many nights, weeks, or months did you stay at the Auburn Hotel with someone else in the past 6 months?

__	MONTHS
__	WEEKS
__	DAYS
8	Refuse to Answer (Months)

If HOU113A is not equal to 1, then skip to instruction before HOU131.

HOU130. About how many nights, weeks, or months did you stay at the Baldwin Hotel with someone else in the past 6 months?

__	MONTHS
__	WEEKS
__	DAYS
8	Refuse to Answer (Months)

If HOU113B is not equal to 1, then skip to instruction before HOU132.

HOU131. About how many nights, weeks, or months did you stay at the Balmoral South Hotel with someone else in the past 6 months?

__	MONTHS
__	WEEKS
__	DAYS
8	Refuse to Answer (Months)

If HOU113C is not equal to 1, then skip to instruction before HOU133.

HOU132. About how many nights, weeks, or months did you stay at the Bayanihan with someone else in the past 6 months?

__	MONTHS
__	WEEKS
__	DAYS
8	Refuse to Answer (Months)

If HOU113D is not equal to 1, then skip to instruction before HOU134.

HOU133. About how many nights, weeks, or months did you stay at the Boston Hotel with someone else in the past 6 months?

__	MONTHS
__	WEEKS
__	DAYS
8	Refuse to Answer (Months)

If HOU113E is not equal to 1, then skip to instruction before HOU135.

HOU134. About how many nights, weeks, or months did you stay at the Boyd Hotel with someone else in the past 6 months?

__	MONTHS
__	WEEKS
__	DAYS
8	Refuse to Answer (Months)

If HOU113F is not equal to 1, then skip to instruction before HOU136.

HOU135. About how many nights, weeks, or months did you stay at the Bristol Hotel with someone else in the past 6 months?

__	MONTHS
__	WEEKS
__	DAYS
8	Refuse to Answer (Months)

If HOU113G is not equal to 1, then skip to instruction before HOU137.

HOU136. About how many nights, weeks, or months did you stay at the Broderick Residential Care Hotel with someone else in the past 6 months?

__	MONTHS
__	WEEKS
__	DAYS
8	Refuse to Answer (Months)

If HOU113H is not equal to 1, then skip to instruction before HOU138.

HOU137. About how many nights, weeks, or months did you stay at the CalDrake with someone else in the past 6 months?

__	MONTHS
__	WEEKS
__	DAYS
8	Refuse to Answer (Months)

If HOU113I is not equal to 1, then skip to instruction before HOU139.

HOU138. About how many nights, weeks, or months did you stay at the Cambridge with someone else in the past 6 months?

__	MONTHS
__	WEEKS
__	DAYS
8	Refuse to Answer (Months)

If HOU113J is not equal to 1, then skip to instruction before HOU140.

HOU139. About how many nights, weeks, or months did you stay at the Camelot Hotel with someone else in the past 6 months?

__	MONTHS
__	WEEKS
__	DAYS
8	Refuse to Answer (Months)

If HOU113K is not equal to 1, then skip to instruction before HOU141.

HOU140. About how many nights, weeks, or months did you stay at the Canon Kip with someone else in the past 6 months?

__	MONTHS
__	WEEKS
__	DAYS
8	Refuse to Answer (Months)

If HOU113L is not equal to 1, then skip to instruction before HOU142.

HOU141. About how many nights, weeks, or months did you stay at the CCR with someone else in the past 6 months?

__	MONTHS
__	WEEKS
__	DAYS
8	Refuse to Answer (Months)

If HOU113M is not equal to 1, then skip to instruction before HOU143.

HOU142. About how many nights, weeks, or months did you stay at the Civic Center Hotel with someone else in the past 6 months?

__	MONTHS
__	WEEKS
__	DAYS
8	Refuse to Answer (Months)

If HOU113N is not equal to 1, then skip to instruction before HOU144.

HOU143. About how many nights, weeks, or months did you stay at the Coast Hotel with someone else in the past 6 months?

__	MONTHS
__	WEEKS
__	DAYS
8	Refuse to Answer (Months)

If HOU113O is not equal to 1, then skip to instruction before HOU145.

HOU144. About how many nights, weeks, or months did you stay at the Coronado Hotel with someone else in the past 6 months?

__	MONTHS
__	WEEKS
__	DAYS
8	Refuse to Answer (Months)

If HOU113P is not equal to 1, then skip to instruction before HOU146.

HOU145. About how many nights, weeks, or months did you stay at the Covered Wagon with someone else in the past 6 months?

__	MONTHS
__	WEEKS
__	DAYS
8	Refuse to Answer (Months)

If HOU113Q is not equal to 1, then skip to instruction before HOU147.

HOU146. About how many nights, weeks, or months did you stay at the CR Hotels with someone else in the past 6 months?

__	MONTHS
__	WEEKS
__	DAYS
8	Refuse to Answer (Months)

If HOU113R is not equal to 1, then skip to instruction before HOU148.

HOU147. About how many nights, weeks, or months did you stay at the Crown Hotel with someone else in the past 6 months?

__	MONTHS
__	WEEKS
__	DAYS
8	Refuse to Answer (Months)

If HOU114A is not equal to 1, then skip to instruction before HOU149.

HOU148. About how many nights, weeks, or months did you stay at the Dahlia Hotel with someone else in the past 6 months?

__	MONTHS
__	WEEKS
__	DAYS
8	Refuse to Answer (Months)

If HOU114B is not equal to 1, then skip to instruction before HOU150.

HOU149. About how many nights, weeks, or months did you stay at the Dalt with someone else in the past 6 months?

__	MONTHS
__	WEEKS
__	DAYS
8	Refuse to Answer (Months)

If HOU114C is not equal to 1, then skip to instruction before HOU151.

HOU150. About how many nights, weeks, or months did you stay at the Desmond with someone else in the past 6 months?

__	MONTHS
__	WEEKS
__	DAYS
8	Refuse to Answer (Months)

If HOU114D is not equal to 1, then skip to instruction before HOU152.

HOU151. About how many nights, weeks, or months did you stay at the Drake Hotel with someone else in the past 6 months?

__	MONTHS
__	WEEKS
__	DAYS
8	Refuse to Answer (Months)

If HOU114E is not equal to 1, then skip to instruction before HOU153.

HOU152. About how many nights, weeks, or months did you stay at the Dudley with someone else in the past 6 months?

__	MONTHS
__	WEEKS
__	DAYS
8	Refuse to Answer (Months)

If HOU114F is not equal to 1, then skip to instruction before HOU154.

HOU153. About how many nights, weeks, or months did you stay at the Elk with someone else in the past 6 months?

__	MONTHS
__	WEEKS
__	DAYS
8	Refuse to Answer (Months)

If HOU114G is not equal to 1, then skip to instruction before HOU155.

HOU154. About how many nights, weeks, or months did you stay at the Ellis Hotel with someone else in the past 6 months?

__	MONTHS
__	WEEKS
__	DAYS
8	Refuse to Answer (Months)

If HOU114H is not equal to 1, then skip to instruction before HOU156.

HOU155. About how many nights, weeks, or months did you stay at the Elm Hotel with someone else in the past 6 months?

__	MONTHS
__	WEEKS
__	DAYS
8	Refuse to Answer (Months)

If HOU114I is not equal to 1, then skip to instruction before HOU157.

HOU156. About how many nights, weeks, or months did you stay at the Empress Hotel with someone else in the past 6 months?

__	MONTHS
__	WEEKS
__	DAYS
8	Refuse to Answer (Months)

If HOU114J is not equal to 1, then skip to instruction before HOU158.

HOU157. About how many nights, weeks, or months did you stay at the Folsom/Dore Hotel with someone else in the past 6 months?

__	MONTHS
__	WEEKS
__	DAYS
8	Refuse to Answer (Months)

If HOU114K is not equal to 1, then skip to instruction before HOU159.

HOU158. About how many nights, weeks, or months did you stay at the Grand Southern Hotel with someone else in the past 6 months?

__	MONTHS
__	WEEKS
__	DAYS
8	Refuse to Answer (Months)

If HOU114L is not equal to 1, then skip to instruction before HOU160.

HOU159. About how many nights, weeks, or months did you stay at the Graystone with someone else in the past 6 months?

__	MONTHS
__	WEEKS
__	DAYS
8	Refuse to Answer (Months)

If HOU114M is not equal to 1, then skip to instruction before HOU161.

HOU160. About how many nights, weeks, or months did you stay at the Hamlin with someone else in the past 6 months?

__	MONTHS
__	WEEKS
__	DAYS
8	Refuse to Answer (Months)

If HOU114N is not equal to 1, then skip to instruction before HOU162.

HOU161. About how many nights, weeks, or months did you stay at the Hartland Hotel with someone else in the past 6 months?

__	MONTHS
__	WEEKS
__	DAYS
8	Refuse to Answer (Months)

If HOU114O is not equal to 1, then skip to instruction before HOU163.

HOU162. About how many nights, weeks, or months did you stay at the Henry Hotel with someone else in the past 6 months?

__	MONTHS
__	WEEKS
__	DAYS
8	Refuse to Answer (Months)

If HOU114P is not equal to 1, then skip to instruction before HOU164.

HOU163. About how many nights, weeks, or months did you stay at the Hillsdale with someone else in the past 6 months?

__	MONTHS
__	WEEKS
__	DAYS
8	Refuse to Answer (Months)

If HOU114Q is not equal to 1, then skip to instruction before HOU165.

HOU164. About how many nights, weeks, or months did you stay at the Hotel Union with someone else in the past 6 months?

__	MONTHS
__	WEEKS
__	DAYS
8	Refuse to Answer (Months)

If HOU115A is not equal to 1, then skip to instruction before HOU166.

HOU165. About how many nights, weeks, or months did you stay at the Jefferson with someone else in the past 6 months?

__	MONTHS
__	WEEKS
__	DAYS
8	Refuse to Answer (Months)

If HOU115B is not equal to 1, then skip to instruction before HOU167.

HOU166. About how many nights, weeks, or months did you stay at the Kean Hotel with someone else in the past 6 months?

__	MONTHS
__	WEEKS
__	DAYS
8	Refuse to Answer (Months)

If HOU115C is not equal to 1, then skip to instruction before HOU168.

HOU167. About how many nights, weeks, or months did you stay at the Kinney Hotel with someone else in the past 6 months?

__	MONTHS
__	WEEKS
__	DAYS
8	Refuse to Answer (Months)

If HOU115D is not equal to 1, then skip to instruction before HOU169.

HOU168. About how many nights, weeks, or months did you stay at the Knox with someone else in the past 6 months?

__	MONTHS
__	WEEKS
__	DAYS
8	Refuse to Answer (Months)

If HOU115E is not equal to 1, then skip to instruction before HOU170.

HOU169. About how many nights, weeks, or months did you stay at the Le Nain Hotel with someone else with someone else in the past 6 months?

__	MONTHS
__	WEEKS
__	DAYS
8	Refuse to Answer (Months)

If HOU115F is not equal to 1, then skip to instruction before HOU171.

HOU170. About how many nights, weeks, or months did you stay at the Leroy Looper with someone else in the past 6 months?

__	MONTHS
__	WEEKS
__	DAYS
8	Refuse to Answer (Months)

If HOU115G is not equal to 1, then skip to instruction before HOU172.

HOU171. About how many nights, weeks, or months did you stay at the Lifeboat Lodge Hotel with someone else in the past 6 months?

__	MONTHS
__	WEEKS
__	DAYS
8	Refuse to Answer (Months)

If HOU115H is not equal to 1, then skip to instruction before HOU173.

HOU172. About how many nights, weeks, or months did you stay at the Lyric with someone else in the past 6 months?

__	MONTHS
__	WEEKS
__	DAYS
8	Refuse to Answer (Months)

If HOU115I is not equal to 1, then skip to instruction before HOU174.

HOU173. About how many nights, weeks, or months did you stay at the Mary Elizabeth Inn with someone else in the past 6 months?

__	MONTHS
__	WEEKS
__	DAYS
8	Refuse to Answer (Months)

If HOU115J is not equal to 1, then skip to instruction before HOU175.

HOU174. About how many nights, weeks, or months did you stay at the McAllister Hotel with someone else in the past 6 months?

__	MONTHS
__	WEEKS
__	DAYS
8	Refuse to Answer (Months)

If HOU115K is not equal to 1, then skip to instruction before HOU176.

HOU175. About how many nights, weeks, or months did you stay at the Mentone with someone else in the past 6 months?

__	MONTHS
__	WEEKS
__	DAYS
8	Refuse to Answer (Months)

If HOU115L is not equal to 1, then skip to instruction before HOU177.

HOU176. About how many nights, weeks, or months did you stay at the Minna Lee Hotel with someone else in the past 6 months?

__	MONTHS
__	WEEKS
__	DAYS
8	Refuse to Answer (Months)

If HOU115M is not equal to 1, then skip to instruction before HOU178.

HOU177. About how many nights, weeks, or months did you stay at the Mission Creek Apartments with someone else in the past 6 months?

__	MONTHS
__	WEEKS
__	DAYS
8	Refuse to Answer (Months)

If HOU115N is not equal to 1, then skip to instruction before HOU179.

HOU178. About how many nights, weeks, or months did you stay at the Mission Hotel with someone else in the past 6 months?

__	MONTHS
__	WEEKS
__	DAYS
8	Refuse to Answer (Months)

If HOU115O is not equal to 1, then skip to instruction before HOU180.

HOU179. About how many nights, weeks, or months did you stay at the National Hotel with someone else in the past 6 months?

__	MONTHS
__	WEEKS
__	DAYS
8	Refuse to Answer (Months)

If HOU115P is not equal to 1, then skip to instruction before HOU181.

HOU180. About how many nights, weeks, or months did you stay at the Neil with someone else in the past 6 months?

__	MONTHS
__	WEEKS
__	DAYS
8	Refuse to Answer (Months)

If HOU116A is not equal to 1, then skip to instruction before HOU182.

HOU181. About how many nights, weeks, or months did you stay at the Oakwood Hotel with someone else in the past 6 months?

__	MONTHS
__	WEEKS
__	DAYS
8	Refuse to Answer (Months)

If HOU116B is not equal to 1, then skip to instruction before HOU183.

HOU182. About how many nights, weeks, or months did you stay at the Orlando Hotel with someone else in the past 6 months?

__	MONTHS
__	WEEKS
__	DAYS
8	Refuse to Answer (Months)

If HOU116D is not equal to 1, then skip to instruction before HOU184.

HOU183. About how many nights, weeks, or months did you stay at the Pacific Bay Inn with someone else in the past 6 months?

__	MONTHS
__	WEEKS
__	DAYS
8	Refuse to Answer (Months)

If HOU116E is not equal to 1, then skip to instruction before HOU185.

HOU184. About how many nights, weeks, or months did you stay at the Pierre with someone else in the past 6 months?

__	MONTHS
__	WEEKS
__	DAYS
8	Refuse to Answer (Months)

If HOU116F is not equal to 1, then skip to instruction before HOU186.

HOU185. About how many nights, weeks, or months did you stay at the Plaza Hotel with someone else in the past 6 months?

__	MONTHS
__	WEEKS
__	DAYS
8	Refuse to Answer (Months)

If HOU116G is not equal to 1, then skip to instruction before HOU187.

HOU186. About how many nights, weeks, or months did you stay at the Prita with someone else in the past 6 months?

__	MONTHS
__	WEEKS
__	DAYS
8	Refuse to Answer (Months)

If HOU116H is not equal to 1, then skip to instruction before HOU188.

HOU187. About how many nights, weeks, or months did you stay at the Rahda with someone else in the past 6 months?

__	MONTHS
__	WEEKS
__	DAYS
8	Refuse to Answer (Months)

If HOU116I is not equal to 1, then skip to instruction before HOU189.

HOU188. About how many nights, weeks, or months did you stay at the Raymond Hotel with someone else in the past 6 months?

__	MONTHS
__	WEEKS
__	DAYS
8	Refuse to Answer (Months)

If HOU116J is not equal to 1, then skip to instruction before HOU190.

HOU189. About how many nights, weeks, or months did you stay at the Ritz with someone else in the past 6 months?

__	MONTHS
__	WEEKS
__	DAYS
8	Refuse to Answer (Months)

If HOU116K is not equal to 1, then skip to instruction before HOU191.

HOU190. About how many nights, weeks, or months did you stay at the Riviera Hotel with someone else in the past 6 months?

__	MONTHS
__	WEEKS
__	DAYS
8	Refuse to Answer (Months)

If HOU116L is not equal to 1, then skip to instruction before HOU192.

HOU191. About how many nights, weeks, or months did you stay at the Rose with someone else in the past 6 months?

__	MONTHS
__	WEEKS
__	DAYS
8	Refuse to Answer (Months)

If HOU116M is not equal to 1, then skip to instruction before HOU193.

HOU192. About how many nights, weeks, or months did you stay at the Royan with someone else in the past 6 months?

__	MONTHS
__	WEEKS
__	DAYS
8	Refuse to Answer (Months)

If HOU117A is not equal to 1, then skip to instruction before HOU194.

HOU193. About how many nights, weeks, or months did you stay at the Seneca with someone else in the past 6 months?

__	MONTHS
__	WEEKS
__	DAYS
8	Refuse to Answer (Months)

If HOU117B is not equal to 1, then skip to instruction before HOU195.

HOU194. About how many nights, weeks, or months did you stay at the Seneca Hotel with someone else in the past 6 months?

__	MONTHS
__	WEEKS
__	DAYS
8	Refuse to Answer (Months)

If HOU117C is not equal to 1, then skip to instruction before HOU196.

HOU195. About how many nights, weeks, or months did you stay at the Star Hotel with someone else in the past 6 months?

__	MONTHS
__	WEEKS
__	DAYS
8	Refuse to Answer (Months)

If HOU117D is not equal to 1, then skip to instruction before HOU197.

HOU196. About how many nights, weeks, or months did you stay at the Sunnyside Hotel with someone else in the past 6 months?

__	MONTHS
__	WEEKS
__	DAYS
8	Refuse to Answer (Months)

If HOU117E is not equal to 1, then skip to instruction before HOU198.

HOU197. About how many nights, weeks, or months did you stay at the Vincent with someone else in the past 6 months?

__	MONTHS
__	WEEKS
__	DAYS
8	Refuse to Answer (Months)

If HOU117F is not equal to 1, then skip to instruction before HOU199.

HOU198. About how many nights, weeks, or months did you stay at the Vincent Hotel with someone else in the past 6 months?

__	MONTHS
__	WEEKS
__	DAYS
8	Refuse to Answer (Months)

If HOU117G is not equal to 1, then skip to instruction before HOU200.

HOU199. About how many nights, weeks, or months did you stay at the Warfield Hotel with someone else in the past 6 months?

__	MONTHS
__	WEEKS
__	DAYS
8	Refuse to Answer (Months)

If HOU117H is not equal to 1, then skip to instruction before HOU201.

HOU200. About how many nights, weeks, or months did you stay at the West Hotel with someone else in the past 6 months?

__	MONTHS
__	WEEKS
__	DAYS
8	Refuse to Answer (Months)

If HOU117I is not equal to 1, then skip to instruction before HOU202.

HOU201. About how many nights, weeks, or months did you stay at the William Penn with someone else in the past 6 months?

__	MONTHS
__	WEEKS
__	DAYS
8	Refuse to Answer (Months)

If HOU117J is not equal to 1, then skip to instruction before HOU203.

HOU202. About how many nights, weeks, or months did you stay at the Windsor Hotel with someone else in the past 6 months?

__	MONTHS
__	WEEKS
__	DAYS
8	Refuse to Answer (Months)

If HOU117K is not equal to 1, then skip to HOU209.

HOU203. Please specify the name of the first "other" SRO where you stayed with someone else.

— —
— — — — — — — — — —

HOU204. About how many nights, weeks, or months did you stay at the [Response to HOU203] with someone else in the past 6 months?

__	MONTHS
__	WEEKS
__	DAYS
8	Refuse to Answer (Months)

If HOU117L is not equal to 1, then skip to HOU209.

HOU205. Please specify the name of the second "other" SRO where you stayed with someone else.

— —
— — — — — — — — — —

HOU206. About how many nights, weeks, or months did you stay at the [Response to HOU205] with someone else in the past 6 months?

__	MONTHS
__	WEEKS
__	DAYS
8	Refuse to Answer (Months)

If HOU117M is not equal to 1, then skip to HOU209.

HOU207. Please specify the name of the third "other" SRO where you stayed with someone else.

— —
— — — — — — — — — —

HOU208. About how many nights, weeks, or months did you stay at the [Response to HOU207] with someone else in the past 6 months?

__	MONTHS
__	WEEKS
__	DAYS
8	Refuse to Answer (Months)

HOU209. In a hotel or motel (like a Motel 6)?

1	Yes
0	No
8	Refuse to Answer

If HOU209 is not equal to 1, then skip to instruction before HOU211.

HOU210. About how many nights, weeks, or months have you stayed in a hotel or motel (like a Motel 6) in the past 6 months?

__	MONTHS
__	WEEKS
__	DAYS
8	Refuse to Answer (Months)

If HOU210 is greater than 6 then Number is too big. Please review. and skip to HOU210.

HOU211. In your own private apartment or house?

1	Yes
0	No
8	Refuse to Answer

If HOU211 is not equal to 1, then skip to instruction before HOU213.

HOU212. About how many nights, weeks, or months have you stayed in your own private apartment or house in the past 6 months?

__	MONTHS
__	WEEKS
__	DAYS
8	Refuse to Answer (Months)

If HOU212 is greater than 6 then Number is too big. Please review. and skip to HOU212.

HOU213. In the past 6 months, have you stayed in someone else's house or apartment (not a SRO)?

1	Yes
0	No
8	Refuse to Answer

If HOU213 is not equal to 1, then skip to instruction before HOU215.

HOU214. About how many nights, weeks, or months have you stayed in someone else's house or apartment (not a SRO) in the past 6 months?

__	MONTHS
__	WEEKS
__	DAYS
8	Refuse to Answer (Months)

If HOU214 is greater than 6 then Number is too big. Please review. and skip to HOU214.

HOU215. Transitional housing (such as the Ashbury House, Baker Street or Jo Ruffin Place)?

1	Yes
0	No
8	Refuse to Answer

If HOU215 is not equal to 1, then skip to instruction before HOU217.

HOU216. About how many nights, weeks, or months have you stayed Transitional housing (such as the Ashbury House, Baker Street or Jo Ruffin Place) in the past 6 months? __ MONTHS

__	WEEKS
__	DAYS
8	Refuse to Answer (Months)

If HOU216 is greater than 6 then Number is too big. Please review. and skip to HOU216.

HOU217. In jail, prison or other legal detention?

1	Yes
0	No
8	Refuse to Answer

If HOU217 is not equal to 1, then skip to instruction before HOU219.

HOU218. About how many nights, weeks, or months have you stayed in jail, prison or other legal detention in the past 6 months?

__	MONTHS
__	WEEKS
__	DAYS
8	Refuse to Answer (Months)

If HOU218 is greater than 6 then Number is too big. Please review. and skip to HOU218.

HOU219. In a hospital or psychiatric ward (such as such as SFGH, being 5150'd)?

1	Yes
0	No
8	Refuse to Answer

If HOU219 is not equal to 1, then skip to instruction before HOU221.

HOU220. About how many nights, weeks, or months have you stayed in a hospital or psychiatric ward (such as such as SFGH, being 5150'd) in the past 6 months?

__	MONTHS
__	WEEKS
__	DAYS
8	Refuse to Answer (Months)

If HOU220 is greater than 6 then Number is too big. Please review. and skip to HOU220.

HOU221. In the past 6 months, have you stayed in a long term drug or alcohol treatment or detox facility (where you stayed more than 90 days such as Jelani House, HAFCI, Walden House, Ozanam)?

1	Yes
0	No
8	Refuse to Answer

If HOU221 is not equal to 1, then skip to instruction before HOU223.

HOU222. About how many nights, weeks, or months have you stayed in a long term drug or alcohol treatment or detox facility in the past 6 months?

__	MONTHS
__	WEEKS
__	DAYS
8	Refuse to Answer (Months)

If HOU222 is greater than 6 then Number is too big. Please review. and skip to HOU222.

HOU223. A Half-way House such as corrections, drug, or psychiatric (such as Shrader House, Baker Places or La Posada)?

1	Yes
0	No
8	Refuse to Answer

If HOU223 is not equal to 1, then skip to instruction before HOU225.

HOU224. About how many nights, weeks, or months have you stayed in a Half-way House such as corrections, drug, or psychiatric in the past 6 months?

__	MONTHS
__	WEEKS
__	DAYS
8	Refuse to Answer (Months)

If HOU224 is greater than 6 then Number is too big. Please review. and skip to HOU224.

HOU225. Long term boarding house, board and care, or nursing home (such as Conard House, Peter Claver or Progress Foundation)?

1	Yes
0	No
8	Refuse to Answer

If HOU225 is not equal to 1, then skip to instruction before HOU227.

HOU226. About how many nights, weeks, or months have you stayed in long term boarding house, board and care, or nursing home in the past 6 months?

__ MONTHS
__ WEEKS
__ DAYS
8 Refuse to Answer (Months)

If HOU226 is greater than 6 then Number is too big. Please review. and skip to HOU226.

HOU227. An encampment or with a group of people in a temporary makeshift shelter?

1 Yes
0 No
8 Refuse to Answer

If HOU227 is not equal to 1, then skip to instruction before HOU229.

HOU228. About how many nights, weeks, or months have you stayed in an encampment or with a group of people in a temporary makeshift shelter in the past 6 months?

__ MONTHS
__ WEEKS
__ DAYS
8 Refuse to Answer (Months)

If HOU228 is greater than 6 then Number is too big. Please review. and skip to HOU228.

HOU229. In the past 6 months, have you stayed in some other place?

1 Yes
0 No
8 Refuse to Answer

If HOU229 is not equal to 1, then skip to HOU232.

HOU230. About how many nights, weeks, or months have you stayed in some other place in the past 6 months?

__ MONTHS
__ WEEKS
__ DAYS
8 Refuse to Answer (Months)

If HOU230 is greater than 6 then Number is too big. Please review. and skip to HOU230.

If HOU230 is equal to 0 or HOU230 is equal to 8, then skip to HOU232.

HOU231. Please specify what other place?

— —
— — — — — — — — — —

HOU232. How many months, weeks, days, have you received housing from Care not Cash in the past 6 months?

__	MONTHS
__	WEEKS
__	DAYS
8	Refuse to Answer (Months)

If SUMD is less than 6 or SUMD is not less than 7, then skip to instruction before HOU233.

If SUMD is less than 5 or SUMD is not less than 6, then skip to instruction before HOU233.

If SUMD is less than 4 or SUMD is not less than 5, then skip to instruction before HOU233.

If SUMD is less than 3 or SUMD is not less than 4, then skip to instruction before HOU233.

If SUMD is less than 2 or SUMD is not less than 3, then skip to instruction before HOU233.

If SUMD is less than 1 or SUMD is not less than 2, then skip to instruction before HOU233.

If SUMD is not less than 1 or SUMD is less than 0, then skip to instruction before HOU233.

If SUMW is less than 6 or SUMW is not less than 7, then skip to instruction before HOU233.

If SUMW is less than 5 or SUMW is not less than 6, then skip to instruction before HOU233.

If SUMW is less than 4 or SUMW is not less than 5, then skip to instruction before HOU233.

If SUMW is less than 3 or SUMW is not less than 4, then skip to instruction before HOU233.

If SUMW is less than 2 or SUMW is not less than 3, then skip to instruction before HOU233.

If SUMW is less than 1 or SUMW is not less than 2, then skip to instruction before HOU233.

If SUMW is less than 0 or SUMW is not less than 1, then skip to instruction before HOU233.

[RESI1M] [RESI1B] [RESI1W] [RESI1C] [RESI1D] [RESI1A]
[RESI2M] [RESI2B] [RESI2W] [RESI2C] [RESI2D] [RESI2A]
[RESI3M] [RESI3B] [RESI3W] [RESI3C] [RESI3D] [RESI3A]
[RESI4M] [RESI4B] [RESI4W] [RESI4C] [RESI4D] [RESI4A]
[RESI5M] [RESI5B] [RESI5W] [RESI5C] [RESI5D] [RESI5A]
[RESI6M] [RESI6B] [RESI6W] [RESI6C] [RESI6D] [RESI6A]
[RESI7M] [RESI7B] [RESI7W] [RESI7C] [RESI7D] [RESI7A]
[RESI8M] [RESI8B] [RESI8W] [RESI8C] [RESI8D] [RESI8A]
[RESI9M] [RESI9B] [RESI9W] [RESI9C] [RESI9D] [RESI9A]
[RESI10M] [RESI10B] [RESI10W] [RESI10C] [RESI10D] [RESI10A]
[RESI11M] [RESI11B] [RESI11W] [RESI11C] [RESI11D] [RESI11A]
[RESI12M] [RESI12B] [RESI12W] [RESI12C] [RESI12D] [RESI12A]
[RESI13M] [RESI13B] [RESI13W] [RESI13C] [RESI13D] [RESI13A]
[RESI14M] [RESI14B] [RESI14W] [RESI14C] [RESI14D] [RESI14A]
[RESI15M] [RESI15B] [RESI15W] [RESI15C] [RESI15D] [RESI15A]
[RESI16M] [RESI16B] [RESI16W] [RESI16C] [RESI16D] [RESI16A]
[RESI17M] [RESI17B] [RESI17W] [RESI17C] [RESI17D] [RESI17A] [HOU17SA]
[RESI18M] [RESI18B] [RESI18W] [RESI18C] [RESI18D] [RESI18A]
A total of [MONTHS] months, [WEEKW] weeks, and [DAYSD1] days.

If MONTHS is not equal to 6 then The total time in the calendar doesn't total 6 months. Please review your answer.

[Interviewer note: If the participant Refused any question, this will throw off the totals.].

HOU233. In the past 6 months, what neighborhood did you live in (or spend the most nights in)? (Choose one)

01	Bay View
02	Excelsior
03	Mission
04	Potrero
05	Tenderloin
06	Western Addition
07	South of Market (SOMA)
08	Other San Francisco Neighborhood
09	Outside of San Francisco
10	Outside of California (specify)
98	Refuse to Answer

If HOU233 is not equal to 10, then skip to HOU235.

HOU234. Speicify where R spent most of her time outside of California

— —

HOU235. Did any of the places you stayed at in the past 6 months offer supportive services on-site like health care, counseling, drug treatment, case management or social services?

1	Yes
0	No
8	Refuse to Answer

HOU236. In total, how many different places did you spend the night in the past 6 months?

__ __	places
98	Refuse to Answer

If HOU1 is equal to 0 and HOU3 is equal to 0 and HOU5 is equal to 0 and HOU7 is equal to 0 and HOU9 is equal to 0 and HOU229 is equal to 0, then skip to instruction before HOU239.

HOU237. In the past 6 months, what were some of the reasons for staying in a homeless shelter or for living on the streets? (Check all that apply) (Check all that apply)

__	Ran
__	Evic
__	Leas
__	Pres
__	Pres
__	Brok
__	Bein
__	Didn
__	Didn
__	Didn
__	Lost
__	Cops
__	It wa
__	Beca
__	Bad
__	Foun
__	Left
__	Drug
__	Men
__	Lost
__	I had
__	Othe
__	Refu

If HOU237 is less than 1 and HOU237 is not equal to 98 then Please choose at least one response from the list. and skip to HOU237.

If HOU237Z is not equal to 1, then skip to instruction before HOU239.

HOU238. Please specify other reason:

__ __
__ __ __ __ __ __ __ __ __ __

If HOU11 is not equal to 1 and HOU211 is not equal to 1, then skip to instruction before HOU243.

If HOU236 is less than 2, then skip to instruction before HOU241.

HOU239. In the past 6 months, what were some of your reasons for leaving your own [PLACES]? (Check all that apply) (Check all that apply)

__ Ran
__ Evic
__ Leas
__ Pres
__ Pres
__ Four
__ Four
__ Brok
__ Bein
__ Didn
__ Didn
__ Didn
__ Lost
__ Cops
__ Beca
__ Tem
__ Did
__ Leav
__ Drug
__ Men
__ Lost
__ I had
__ Othe
__ Refu

If HOU239 is less than 1 and HOU239 is not equal to 98 then Please choose at least one response from the list. and skip to HOU239.

If HOU239Z is not equal to 1, then skip to instruction before HOU241.

HOU240. Please specify other reason:

— —
— — — — — — — — — —

If HOU237B is not equal to 1 and HOU239B is not equal to 1, then skip to instruction before HOU243.

HOU241. What was the main reason for the eviction? (Choose one)

1 Didn't or coul
2 Broke rules
3 Got arrested
4 Roommate or
5 Left the prem
6 Weren't payin
7 Other
8 Refuse to Ans

If HOU241 is not equal to 7, then skip to instruction before HOU243.

HOU242. Please specify other reason:

— —

If HOU11 is not equal to 1 and HOU110 is not equal to 1, then skip to instruction before HOU246.

HOU243. In the past 6 months, did you stay at an SRO with a front desk or security system that does not allow entrance to people who don't live there?

1 Yes
0 No
8 Refuse to Answer

HOU244. During the past 6 months, were you ever 21-day'd from an SRO, or forced to move out of an SRO room then allowed to move back in to prevent you from getting tenant status? 1 Yes

0 No
8 Refuse to Answer

If HOU11 is not equal to 1, then skip to instruction before HOU246.

HOU245. Did you have a signed lease agreement in your own name at any of the hotels you stayed at in the past 6 months?

1 Yes
0 No
8 Refuse to Answer

If HOU211 is not equal to 1, then skip to instruction before HOU247.

HOU246. Did you have a signed lease agreement in your own name at your private apartment or house at all times in the past 6 months?

1 Yes
0 No
8 Refuse to Answer

If HOU11 is not equal to 1 and HOU110 is not equal to 1 and HOU211 is not equal to 1 and HOU213 is not equal to 1, then skip to HOU248.

HOU247. On average, how many people were sleeping in the same room/place with you during the past 6 months?

	__ __	people
	98	Refuse to Answer

HOU248. Thinking about the place where you're currently staying; during the past 6 months (or since you've been there if you've been there less than 6 months):

Has anyone threatened to harm you?	1	Yes
	0	No
	8	Refuse to Answer

HOU249. Thinking about the place where you're currently staying; during the past 6 months (or since you've been there if you've been there less than 6 months):

Is it common for people to be physically harmed or assaulted there?	1	Yes
	0	No
	8	Refuse to Answer

HOU250. Is it common for people to sell drugs there?	1	Yes
	0	No
	8	Refuse to Answer

If Q3 is equal to 1, then skip to instruction before CHL1.

If COUNT is not equal to 0, then skip to instruction before CHL1.

[BROKEN]

If COUNT is equal to 0, then skip to end of questionnaire.

If COUNTC is not equal to 0, then skip to instruction before CHL1.

[BROKENC]

If COUNTC is equal to 0, then skip to end of questionnaire.

CHILDREN

Now I have some questions about any children you may have.

CHL1. Do you have any living children?

1 Yes
0 No
8 Refuse to Answer

If not (CHILDALL is equal to 0 and CHL1 is equal to 1), then skip to instruction before CHL3.

CHL2. In a previous interview, it appears that we recorded that you **DID NOT** have any children. Were we incorrect, that is, do you currently have any living children? (Choose one)

0 We
1 We were correct last time, but now do have living children
2 Don't have children now
8 Refuse to Answer

If not (CHILDALL is equal to 1 and CHL1 is equal to 0), then skip to instruction before CHL4.

CHL3. In a previous interview, it appears that we recorded that you **DO** have children. Has that changed or do you now have living children? (Choose one)

0 Situation has changed, no longer have children
1 Situation hasn't changed, never had children
2 Still have children
8 Refuse to Answer

If HCD182 is not equal to 1 and Q3 is not equal to 1, then skip to instruction before CHL5.

CHL4. Have you given birth in the past 6-months?

1 Yes
0 No
8 Refuse to Answer

If CHILDNEW is equal to 0, then skip to CHL8.

CHL5. Are any of your children under age 18?

1 Yes
0 No
8 Refuse to Answer

If CHL5 is not equal to 1, then skip to CHL8.

CHL6. Do all of your children under the age of 18 currently live with you?

1 Yes
0 No
8 Refuse to Answer

If CHL6 is not equal to 0, then skip to CHL8.

CHL7. Do any of your children under the age of 18 currently live with you?

1 Yes
0 No
8 Refuse to Answer

CHL8.	Do any other children under age 18 currently live with you?	1	Yes
		0	No
		8	Refuse to Answer

Now I'd like to ask you about any military service involvment.

CHL9.	Have you ever been in the military?	1	Yes
		0	No
		8	Refuse to Answer

If CHL9 is not equal to 1, then skip to instruction before MNY1.

CHL10.	What year were you discharged?	— — — —	yyyy
		2097	Don't Know (Year)
		2098	Refuse to Answer (Year)

MONEY MATTERS

Now let's talk about the ways you may have made money in the last 6 months. Please remember that all of your answers are confidential and will not be reported to other people.

Please answer yes or no.

MNY1. In the past 6 months, have you received any money from:

Employment or a job?

1	Yes
0	No
8	Refuse to Answer

If MNY1 is not equal to 1, then skip to MNY2.

MNY1A. During the last 6 months, how many months did you receive money from **employment or a job**?

__	months
8	Refuse to Answer

MNY1B. During those [Response to MNY1A] months, on average, how much money did you get each month from **employment or a job?**

__ __ __ __ __	dollars
99998	Refuse to Answer

MNY2. Have you received any money from:

GA or CAAP (General Assistance)?

1	Yes
0	No
8	Refuse to Answer

If MNY2 is not equal to 1, then skip to MNY3.

MNY2A. During the last 6 months, how many months did you receive money from **GA or CAAP (General Assistance)**?

__	months
8	Refuse to Answer

MNY2B. During those [Response to MNY2A] months, on average, how much money did you get each month from **GA or CAAP (General Assistance)?**

__ __ __ __ __	dollars
99998	Refuse to Answer

MNY3. Have you received any money from:

TANF (Temporary Assistance for Needy Families)?

1	Yes
0	No
8	Refuse to Answer

If MNY3 is not equal to 1, then skip to MNY4.

MNY3A. During the last 6 months, how many months did you receive money from **TANF (Temporary Assistance for Needy Families)**?

__	months
8	Refuse to Answer

MNY3B. During those [Response to MNY3A] months, on average, how much money did you get each month from **TANF (Temporary Assistance for Needy Families)?** __ __ __ __ _

99998	Refuse to Answer

MNY4. Have you received any money from:

Disability Income, such as SSDI (Social Security Disability Insurance) or SSI (Supplemental Security Income)?

1	Yes
0	No
8	Refuse to Answer

If MNY4 is not equal to 1, then skip to MNY5.

MNY4A. During the last 6 months, how many months did you receive money from **Disability Income, such as SSDI (Social Security Disability Insurance) or SSI (Supplemental Security Income)**?

__	months
8	Refuse to Answer

MNY4B. During those [Response to MNY4A] months, on average, how much money did you get each month from **Disability Income, such as SSDI (Social Security Disability Insurance) or SSI (Supplemental Security Income)?**

__ __ __ __ __	dollars
99998	Refuse to Answer

MNY5. Have you received any money from:

Unemployment Insurance benefits (UIB)?

1	Yes
0	No
8	Refuse to Answer

If MNY5 is not equal to 1, then skip to MNY6.

MNY5A. During the last 6 months, how many months did you receive money from **Unemployment Insurance benefits (UIB)**?

__	months
8	Refuse to Answer

MNY5B. During those [Response to MNY5A] months, on average, how much money did you get each month from **Unemployment Insurance benefits (UIB)?**

__ __ __ __ __	dollars
99998	Refuse to Answer

MNY6. Have you received any money from:

Social Security (not including SSI or SSDI)?

1 Yes
0 No
8 Refuse to Answer

If MNY6 is not equal to 1, then skip to MNY7.

MNY6A. During the last 6 months, how many months did you receive money from **Social Security (not including SSI or SSDI)**?

__ months
8 Refuse to Answer

MNY6B. During those [Response to MNY6A] months, on average, how much money did you get each month from **Social Security (not including SSI or SSDI)?**

__ __ __ __ __ dollars
99998 Refuse to Answer

MNY7. Have you received any money from:

VA benefits?

1 Yes
0 No
8 Refuse to Answer

If MNY7 is not equal to 1, then skip to MNY8.

MNY7A. During the last 6 months, how many months did you receive money from **VA benefits**?

__ months
8 Refuse to Answer

MNY7B. During those [Response to MNY7A] months, on average, how much money did you get each month from **VA benefits?**

__ __ __ __ __ dollars
99998 Refuse to Answer

MNY8. Have you received any money from:

Selling food stamps?

1 Yes
0 No
8 Refuse to Answer

If MNY8 is not equal to 1, then skip to MNY9.

MNY8A. During the last 6 months, how many months did you receive money from **selling food stamps**?

__ months
8 Refuse to Answer

MNY8B. During those [Response to MNY8A] months, on average, how much money did you get each month from **selling food stamps?**

__ __ __ __ __ dollars
99998 Refuse to Answer

MNY9. Have you received any money from:

Selling used items or other things that can be recycled?
1 Yes
0 No
8 Refuse to Answer

If MNY9 is not equal to 1, then skip to MNY10.

MNY9A. During the last 6 months, how many months did you receive money from **selling used items or other things that can be recycled**?

__ months
8 Refuse to Answer

MNY9B. During those [Response to MNY9A] months, on average, how much money did you get each month from **selling used items or other things that can be recycled?** __ __ __ __ _

99998 Refuse to Answer

MNY10. Have you received any money from:

Letting people borrow your room or apartment?
1 Yes
0 No
8 Refuse to Answer

If MNY10 is not equal to 1, then skip to MNY11.

MNY10A. During the last 6 months, how many months did you receive money from **letting people borrow your room or apartment**?

__ months
8 Refuse to Answer

MNY10B. During those [Response to MNY10A] months, on average, how much money did you get each month from **letting people borrow your room or apartment?** __ __ __ __ _

99998 Refuse to Answer

MNY11. Have you received any money from:

Panhandling or asking people for money?
1 Yes
0 No
8 Refuse to Answer

If MNY11 is not equal to 1, then skip to MNY12.

MNY11A. During the last 6 months, how many months did you receive money from **panhandling or asking people for money**
?

__ months
8 Refuse to Answer

MNY11B. During those [Response to MNY11A] months, on average, how much money did you get each month from **panhandling or asking people for money?** __ __ __ __ __ dollars

99998 Refuse to Answer

MNY12. Have you received any money from:

Selling drugs?

1 Yes
0 No
8 Refuse to Answer

If MNY12 is not equal to 1, then skip to MNY13.

MNY12A. During the last 6 months, how many months did you receive money from **selling drugs**?

__ months
8 Refuse to Answer

MNY12B. During those [Response to MNY12A] months, on average, how much money did you get each month from **selling drugs?**

__ __ __ __ __ dollars
99998 Refuse to Answer

MNY13. Have you received any money from:

Having sex with someone for money?

1 Yes
0 No
8 Refuse to Answer

If MNY13 is not equal to 1, then skip to MNY14.

MNY13A. During the last 6 months, how many months did you receive money from **having sex with someone for money**?

__ months
8 Refuse to Answer

MNY13B. During those [Response to MNY13A] months, on average, how much money did you get each month from **having sex with someone for money?**

__ __ __ __ __ dollars
99998 Refuse to Answer

MNY14. Have you received any money from:

(Money given to you by) Friends?

1 Yes
0 No
8 Refuse to Answer

If MNY14 is not equal to 1, then skip to MNY15.

MNY14A. During the last 6 months, how many months did you receive money from **friends**?

__ months
8 Refuse to Answer

MNY14B. During those [Response to MNY14A] months, on average, how much money did you get each month from **friends?**

__ __ __ __ __	dollars
99998	Refuse to Answer

MNY15. Have you received any money from:

(Money given to you by) Family members?

1	Yes
0	No
8	Refuse to Answer

If MNY15 is not equal to 1, then skip to MNY16.

MNY15A. During the last 6 months, how many months did you receive money from **family members**?

__	months
8	Refuse to Answer

MNY15B. During those [Response to MNY15A] months, on average, how much money did you get each month from **family members?**

__ __ __ __ __	dollars
99998	Refuse to Answer

MNY16. During the last 6 months, **h**ave you received any money from:

Research study (other than SHADOW)?

1	Yes
0	No
8	Refuse to Answer

If MNY16 is not equal to 1, then skip to MNY17.

MNY16A. During the last 6 months, how many months did you receive money from **a research study (other than SHADOW)**?

__	months
8	Refuse to Answer

MNY16B. During those [Response to MNY16A] months, on average, how much money did you get each month from **a research study (other than SHADOW)?** __ __ __ __ __ dollars

99998	Refuse to Answer

MNY17. Have you received any money from:

Doing something else?

1	Yes
0	No
8	Refuse to Answer

If MNY17 is not equal to 1, then skip to MNY18.

MNY17A. During the last 6 months, how many months did you receive money from **doing something else**?

__	months
8	Refuse to Answer

MNY17B. During those [Response to MNY17A] months, on average, how much money did you get each month from **doing something else?**

__ __ __ __ __	dollars
99998	Refuse to Answer

MNY18. Not counting banks or credit cards, do you currently owe money to anyone?

1	Yes
0	No
8	Refuse to Answer

If MNY18 is not equal to 1, then skip to instruction before HTH1.

MNY19. How many people have you borrowed money from in the past 6 months?

__ __ __	people
998	Refuse to Answer

MNY20. Did you borrow money to pay for: (Check all that apply) (Check all that apply)

__ a hotel room
__ to pay back a friend
__ to pay back a landlord/hotel manager
__ to pay for drugs
__ to buy food
__ Rent and storage
__ Transportation
__ Living expenses (phone, clothes, personal items, laundry, children's needs)
__ Personal enjoyment (alcohol, cigarettes, magazines, entertainment)
__ other
__ Refuse to Answer

If MNY20 is less than 1 and MNY20 is not equal to 98 then Please choose at least one response from the list. and skip to MNY20.

If MNY20Z is not equal to 1, then skip to instruction before HTH1.

MNY21. Please specify "other" reason you borrowed money:

__ __
__ __ __ __ __ __ __ __ __ __

HEALTH

Now, I am going to ask a question about your general health. Please choose the response that best represents the way you feel. Remember, this is not a test, and there are no right or wrong answers.

HTH1. In general would you say your health is: (Choose one)

1 Excellent
2 Very good
3 Good
4 Fair
5 Or poor?
8 Refuse to Answer

The following questions are about activities that you might do during a typical day. Please tell me if your health limits you a lot, a little, or not at all in:

HTH2. Moderate activities, such as moving a table, pushing a shopping cart, or sweeping (Choose one)

1 Yes, limited a lot
2 Yes, limited a little
3 No, not limited at all
4 Does not do activity
8 Refuse to Answer

HTH3. . . . climbing several flights of stairs. Does your health now limit you a lot, limit you a little, or not limit you at all? (Choose one)

1 Yes, limited a lot
2 Yes, limited a little
3 No, not limited at all
4 Does not do activity
8 Refuse to Answer

HTH4. During the past 4 weeks, have you accomplished less than you would like as a result of your physical health?

1 Yes
0 No
8 Refuse to Answer

HTH5. During the past 4 weeks, were you limited in the kind of work or other regular daily activities you do as a result of your physical health?

1 Yes
0 No
8 Refuse to Answer

HTH6. During the past 4 weeks, have you accomplished less than you would like as a result of any emotional problems, such as feeling depressed or anxious?

1 Yes
0 No
8 Refuse to Answer

HTH7. <u>During the past 4 weeks,</u> did you not do work or other regular daily activities as carefully as usual as a result of any emotional problems, such as feeling depressed or anxious?

1 Yes
0 No
8 Refuse to Answer

HTH8. <u>During the past 4 weeks,</u> how much did pain interfere with your normal work or activities: did it interfere (Choose one)

1 Not at all
2 Slightly
3 Moderately
4 Quite a bit
5 Or extremely?
8 Refuse to Answer

HTH9. <u>During the past 4 weeks,</u> how much of the time has your physical health or emotional problems interfered with your social activities like visiting with friends or relatives: has it interfered (Choose one)

1 All of the time
2 Most of the time
3 Some of the time
4 A little of the time
5 Or none of the time?
8 Refuse to Answer

The next questions are about how you feel and how things have been with you during the past 4 weeks.

GIVE SF-12 CARD TO R.

As I read each statement, please give me the one answer that comes closest to the way you have been feeling; is it all of the time, most of the time, a good bit of the time, some of the time, a little of the time, or none of the time.

HTH10. How much of the time <u>during the past 4 weeks</u> have you felt calm and peaceful? (Choose one)

1 All of the time
2 Most of the time
3 A good bit of the time
4 Some of the time
5 A little bit of the time
6 None of the time
8 Refuse to Answer

HTH11. How much of the time <u>during the past 4 weeks</u> did you have a lot of energy?
(Choose one)

1 All of the time
2 Most of the time
3 A good bit of the time
4 Some of the time
5 A little bit of the time
6 None of the time
8 Refuse to Answer

HTH12. How much of the time <u>during the past 4 weeks</u> have you felt downhearted and depressed? (Choose one)

1 All of the time
2 Most of the time
3 A good bit of the time
4 Some of the time
5 A little bit of the time
6 None of the time
8 Refuse to Answer

These next questions are about the food eaten in your household in the last 6 months, since [DATE6MO], and whether you were able to afford the food you need.

HTH13. I'm going to read you two statements that people have made about their food situation. For these two statements, please tell me whether the statement was <u>often</u> true, <u>sometimes</u> true, or <u>never</u> true for (you/your household) in the last 6 months-that is, since last (name of current month).

The food that (I/we) bought just didn't last, and (I/we) didn't have money to get more. Was that <u>often</u>, <u>sometimes</u>, or <u>never</u> true for (you/your household) in the last 6months? (Choose one)

1 Ofte
2 Sometimes true
3 Never true
8 Refuse to Answer

HTH14. (I/we) couldn't afford to eat balanced meals. Was that <u>often</u>, <u>sometimes</u>, or <u>never</u> true for (you/your household) in the last 6 months? (Choose one)

1 Often true
2 Sometimes true
3 Never true
8 Refuse to Answer

HTH15. In the last 6 months, since last [DATE6MO], did (you/you or other adults in your household) ever cut the size of your meals or skip meals because there wasn't enough money for food?

1 Yes
0 No
8 Refuse to Answer

If HTH15 is not equal to 1, then skip to HTH17.

HTH16. How often did this happen-almost every month, some months but not every month, or in only 1 or 2 months? (Choose one)

0	Almost every month
1	Some months but not every month
2	Only 1 or 2 months
8	Refuse to Answer

HTH17. In the last 6 months, did you ever eat less than you felt you should because there wasn't enough money for food?

1	Yes
0	No
8	Refuse to Answer

HTH18. In the last 6 months, were you every hungry but didn't eat because there wasn't enough money for food?

1	Yes
0	No
8	Refuse to Answer

HIV

Now I have a few other questions about your HIV status.

If Q8 is equal to 1, then skip to instruction before HIV4.

HIV1. Have you been tested for HIV since your last test here?

1 Yes
0 No
7 Don't Know
8 Refuse to Answer

If HIV1 is not equal to 1, then skip to instruction before HIV4.

HIV2. What were the results of that test? (Choose one)

0 Negative
1 Positive
7 Don't Know
8 Refuse to Answer

HIV3. Where did you get that test?

— —

If HIV2 is not equal to 1, then skip to instruction before HCD1.

If Q8 is equal to 0 and HIV2 is not equal to 1, then skip to instruction before HCD1.

Now I will ask you about any support you may have received due to your HIV status.

HIV4. At any time in the past 6 months, did you have a case manager due to your HIV status?

1 Yes
0 No
7 Don't Know
8 Refuse to Answer

HIV5. At any time in the past 6 months, did you have insurance such as Medi-Cal or Medicare due to your HIV status?

1 Yes
0 No
8 Refuse to Answer

HIV6. At any time in the past 6 months, did you receive benefits such as SSI or SSDI due to your HIV status?

1 Yes
0 No
8 Refuse to Answer

HIV7. At any time in the past 6 months, did you receive a rental subsidy or housing assistance due to your HIV status?

1	Yes
0	No
8	Refuse to Answer

HEALTH CONDITIONS

In the past 6 months, have you experienced symptoms or seen a health care provider for any of the following health conditions:

HCD1. Heart disease?
1 Yes
0 No
8 Refuse to Answer

HCD2. High blood pressure?
1 Yes
0 No
8 Refuse to Answer

HCD3. Diabetes?
1 Yes
0 No
8 Refuse to Answer

HCD4. Emphysema?
1 Yes
0 No
8 Refuse to Answer

HCD5. Asthma?
1 Yes
0 No
8 Refuse to Answer

HCD6. Pelvic pain or sexually transmitted infections such as chlamydia or gonorrhea?
1 Yes
0 No
8 Refuse to Answer

HCD7. Hepatitis?
1 Yes
0 No
8 Refuse to Answer

If HCD7 is not equal to 1, then skip to HCD9.

HCD8. Which types of hepatitis have you tested positive for? (Check all that apply) (Check all that apply)

__ Hepatitis A
__ Hepatitis B
__ Hepatitis C
__ Another type of Hepatitis
__ Don't Know
__ Refuse to Answer

HCD9. In the past 6 months, have you experienced symptoms or seen a health care provider for HIV? Includes: HIV testing, PEP, ARVs, Routine Health Care.

1 Yes
0 No
8 Refuse to Answer

If HCD9 is not equal to 1, then skip to HCD11.

HCD10. Where did you receive HIV healthcare, in the past 6-months? (Check all that apply) (Check all that apply)

__ San Francisco General Hospital Ward 86
__ Other SFGH outpatient clinic
__ UCSF Parnassus
__ UCSF Mt. Zion
__ Tom Waddell Health Center
__ Kaiser
__ VA
__ California Pacific Medical Center (CPMC)
__ SOMA Health Center
__ Lyon-Martin Women's Services
__ St. Anthony's Medical Clinic
__ Mission Neighborhood Health Center
__ Other outpatient clinic
__ Refuse to Answer

If HCD10 is less than 1 and HCD10 is not equal to 98 then Please choose at least one response from the list. and skip to HCD10.

HCD11. In the past 6 months, have you experienced symptoms or seen a health care provider for the cold or flu?

1 Yes
0 No
8 Refuse to Answer

HCD12. Pain, including headaches, dental pain, painful feet, or other types of pain?

1 Yes
0 No
8 Refuse to Answer

HCD13. In the past 6 months, have you experienced symptoms or seen a health care provider for depression?

1 Yes
0 No
8 Refuse to Answer

HCD14. Other mental health issues like nerves, panic, or hallucinations?

1 Yes
0 No
8 Refuse to Answer

If HCD13 is not equal to 1 and HCD14 is not equal to 1, then skip to instruction before HCD16.

HCD15. Did you receive mental health counseling or treatment at SFGH?

1 Yes
0 No
8 Refuse to Answer

The next few questions will ask about medications you've taken in the past 6 months.

HCD16. In the past 6 months, have you taken any prescribed medication for:

Pain?

1 Yes
0 No
8 Refuse to Answer

HCD17. In the past 6 months, have you taken any prescribed medication for:

Physical health conditions such as asthma or diabetes?

1 Yes
0 No
8 Refuse to Answer

HCD18. **HIV?**

1 Yes
0 No
8 Refuse to Answer

HCD19. **Sexually transmitted infections?**

1 Yes
0 No
8 Refuse to Answer

If HCD19 is not equal to 1, then skip to HCD27.

HCD20. Were you prescribed medication for any of the following infections during the past 6 months? (Check all that apply) (Check all that apply)

__ Gonorrhea
__ Chlamydia
__ Herpes
__ Trichomonas
__ Hepatitis B
__ Syphilis
__ Refuse to Answer

If HCD20A is not equal to 1, then skip to instruction before HCD22.

HCD21. How much of the medication for **gonorrhea** did you take? (Choose one)

0	I did not fill or pick up the prescription
1	I never actually took any of the medication
2	I started taking the medication but stopped
3	I finished the whole prescription
4	I'm still taking the medication and plan to finish the whole thing
5	Other
7	Don't Know
8	Refuse to Answer
9	Not Applicable

If HCD20B is not equal to 1, then skip to instruction before HCD23.

HCD22. How much of the medication for **chlamydia** did you take? (Choose one)

0	I did not fill or pick up the prescription
1	I never actually took any of the medication
2	I started taking the medication but stopped
3	I finished the whole prescription
4	I'm still taking the medication and plan to finish the whole thing
5	Other
7	Don't Know
8	Refuse to Answer
9	Not Applicable

If HCD20C is not equal to 1, then skip to instruction before HCD24.

HCD23. How much of the medication for **herpes** did you take? (Choose one)

0	I did not fill or pick up the prescription
1	I never actually took any of the medication
2	I started taking the medication but stopped
3	I finished the whole prescription
4	I'm still taking the medication and plan to finish the whole thing
5	Other
7	Don't Know
8	Refuse to Answer
9	Not Applicable

If HCD20D is not equal to 1, then skip to instruction before HCD25.

HCD24. How much of the medication for **trichomonas** did you take? (Choose one)

0 I did not fill or pick up the prescription
1 I never actually took any of the medication
2 I started taking the medication but stopped
3 I finished the whole prescription
4 I'm still taking the medication and plan to finish the whole thing
5 Other
7 Don't Know
8 Refuse to Answer
9 Not Applicable

If HCD20E is not equal to 1, then skip to instruction before HCD26.

HCD25. How much of the medication for **hepatitis B** did you take? (Choose one)

0 I did not fill or pick up the prescription
1 I never actually took any of the medication
2 I started taking the medication but stopped
3 I finished the whole prescription
4 I'm still taking the medication and plan to finish the whole thing
5 Other
7 Don't Know
8 Refuse to Answer
9 Not Applicable

If HCD20F is not equal to 1, then skip to HCD27.

HCD26. How much of the medication for **syphilis** did you take? (Choose one)

0 I did not fill or pick up the prescription
1 I never actually took any of the medication
2 I started taking the medication but stopped
3 I finished the whole prescription
4 I'm still taking the medication and plan to finish the whole thing
5 Other
7 Don't Know
8 Refuse to Answer
9 Not Applicable

HCD27. In the <u>past 6 months</u>, have you taken any prescribed medication for:

Mental health conditions such as depression or anxiety?

1 Yes
0 No
8 Refuse to Answer

HCD28. Have you been pregnant at any time in the past 6 months?

1 Yes
0 No
8 Refuse to Answer

If HCD28 is not equal to 1, then skip to instruction before HCU1.

HCD29. What happened or is happening with this pregnancy? (Choose one)

1	Plan to carry and deliver the baby
2	Miscarried
3	Aborted the pregnancy
4	Unsure at this time (haven't decided)
8	Refuse to Answer

HEALTHCARE USE

Now I'm going to ask you some questions about healthcare you've received. First, I want to ask about any experiences you have had in a hospital emergency room.

HCU1. In past 6 months, did you go to or get taken to an emergency room for your own health?

[Interviewer note: Include ER visits for physical, psych, alcohol, drugs, or any other problem.]

1	Yes
0	No
8	Refuse to Answer

If HCU1 is not equal to 1, then skip to HCU10.

HCU2. Which ER did you go to? (Check all that apply) (Check all that apply)

__ San Francisco General Hospital
__ California Pacific Medical Center (Include Davies Medical Center)
__ St. Francis Memorial Hospital
__ St. Luke's Hospital
__ St. Mary's Hospital
__ Kaiser Permanente
__ VA/Fort Miley
__ UCSF Medical Center (Parnassus Campus)
__ UCSF/Mt. Zion Medical Center
__ Highland Hospital, Oakland
__ Seton Medical Center, Daly City
__ Other, in San Francisco
__ Other, outside SF
__ Refuse to Answer

If HCU2 is less than 1 and HCU2 is not equal to 98 then Please choose at least one response from the list. and skip to HCU2.

HCU3. Were any of these ER visits related to a mental or emotional problem?

[Interviewer note: Include suicide attempts.]

1	Yes
0	No
8	Refuse to Answer

HCU4. Were any of these ER visits related to an accidental drug overdose?

[Interviewer note: Do <u>not</u> include suicide attempts.]

1	Yes
0	No
8	Refuse to Answer

HCU5. Were any of these ER visits related to someone hurting you, such as being beaten, raped, or stabbed?

1 Yes
0 No
8 Refuse to Answer

HCU6. Was hunger a main reason for any of your ER visits?

1 Yes
0 No
8 Refuse to Answer

HCU7. Was lack of shelter a main reason for any of your ER visits?

1 Yes
0 No
8 Refuse to Answer

HCU8. Was fear for your safety a main reason for any of your ER visits?

1 Yes
0 No
8 Refuse to Answer

HCU9. Was getting off the street (because you had no place to stay) a main reason for any of your ER visits?

1 Yes
0 No
8 Refuse to Answer

HCU10. In the past 6 months, did you stay overnight in a hospital bed for any reason?

*[Interviewer note: Include inpatient stays **only**., including overnight stays.*
Include admissions resulting from ER visits.
*Do **not** include outpatient or ER visits that didn't result in hospitlalization. Do **not** count staying overnight in the ER waiting room.*
*Do **not** count overnight detox for drug or alcohol problems.]*

1 Yes
0 No
8 Refuse to Answer

If HCU10 is not equal to 1, then skip to HCU15.

HCU11. Where were you hospitalized? (Were you hospitalized at San Francisco General Hospital or some other hospital?) (Check all that apply) (Check all that apply) __ San

__ California Pacific Medical Center (Include Davies Medical Center)
__ St. Francis Memorial Hospital
__ St. Luke's Hospital
__ St. Mary's Hospital
__ Kaiser Permanente
__ VA/Fort Miley
__ UCSF Medical Center (Parnassus Campus)
__ UCSF/Mt. Zion Medical Center
__ Highland Hospital, Oakland
__ Seton Medical Center, Daly City
__ Other, in San Francisco
__ Other, outside SF
__ Refuse to Answer

If HCU11 is less than 1 and HCU11 is not equal to 98 then Please choose at least one response from the list. and skip to HCU11.

HCU12. Were any of these hospitalizations related to a mental or emotional problem?

[Interviewer note: Include suicide attempts.]

1 Yes
0 No
8 Refuse to Answer

HCU13. Were any of these hospitalizations related to an accidental drug overdose?

[Interviewer note: Do not include suicide attempts.]

1 Yes
0 No
8 Refuse to Answer

HCU14. Were any of these hospitalizations related to someone hurting you, such as being beaten, raped, or stabbed?

1 Yes
0 No
8 Refuse to Answer

HCU15. Did you have any visits with a doctor, nurse, or other healthcare provider that were not in an emergency room or overnight hospital stay?

1 Yes
0 No
8 Refuse to Answer

If HCU15 is not equal to 1, then skip to HCU21.

HCU16. Where have you received outpatient/ambulatory health care in the past 6-months? (Check all that apply) (Check all that apply)

__ A doctor's office or clinic
__ In a supportive housing clinic
__ Outreach or mobile services
__ A hospital in San Francisco
__ A hospital outside of San Francisco
__ Refuse to Answer

If HCU16D is not equal to 1, then skip to HCU18.

HCU17. Was this hospital SFGH?

1 Yes
0 No
8 Refuse to Answer

HCU18. Were any of these for emotional or mental health problems? For example, visits to a therapist, psychologist, or psychiatrist?

1 Yes
0 No
8 Refuse to Answer

If HCU18 is not equal to 0, then skip to HCU20.

HCU19. When it comes to your mental health, what are some reasons you do not get mental health services? (Check all that apply) (Check all that apply)

__ I don't need mental health care
__ I don't know where to go
__ I don't know what to ask for
__ I don't know if I would be accepted there
__ I don't trust the people working there
__ Services aren't offered in my neighborhood
__ Services aren't offered at a good time for me
__ I'm too busy working or taking care of business to go
__ I have to take care of a family member of friend and don't have time
__ My partner doesn't want me to go
__ I've tried the services in the past and they didn't do any good
__ I get started, but I never stick with it long enough to make a difference
__ I'm not in one place long enough for it to make a difference
__ I don't think I can afford it
__ I don't want people to think I'm crazy
__ I don't like how I feel when I'm on the medications they want me to take
__ Refuse to Answer

If HCU19 is less than 1 and HCU19 is not equal to 98 then Please choose at least one response from the list. and skip to HCU19.

HCU20. Were any of these visits related to someone hurting you, such as being beaten, raped, or stabbed?

1	Yes
0	No
8	Refuse to Answer

HCU21. Do you have a case manager or counselor who is supposed to help you get health care on a regular basis?

1	Yes
0	No
8	Refuse to Answer

HCU22. Do you have a case manager or social worker who is supposed to help you get social services for things like housing, SSI or legal assistance?

1	Yes
0	No
8	Refuse to Answer

INSURANCE

INS1. How much of the time during past 6 months have you had any health insurance, such as Medi-Cal, Medicare, or Blue Cross? (Choose one)

0	None of the time
1	A little of the time
2	Some of the time
3	Most of the time
4	All of the time
8	Refuse to Answer

If INS1 is equal to 0 or INS1 is equal to 8, then skip to INS4.

INS2. What type or types of insurance have you had during the past 6 months? (Check all that apply) (Check all that apply)

__	Medi-Cal
__	Medicare
__	Veterans Administration
__	Private (Blue Cross, Blue Shield, Kaiser)
__	San Francisco Health Plan
__	Other
__	Refuse to Answer

If INS2 is less than 1 and INS2 is not equal to 98 then Please choose at least one response from the list. and skip to INS2.

If INS2F is not equal to 1, then skip to INS4.

INS3. Please specify

— —

INS4. In the past 6-months, did you attempt to get any of the following services, but were told that the agency providing the service is now closed or that the service was no longer being offered? (Check all that apply)

[Note: We are asking about services that you used to get and stopped getting in the past 6 months.] (Check all that apply)

__ Homeless shelter
__ Housing
__ Supportive housing
__ Case management
__ Mental health treatment
__ Prescribed mental health medication
__ Prescribed pain medication
__ Substance abuse treatment
__ Health care center or clinic
__ Prescribed medication for physical health conditions like infections, heart disease or diabetes
__ ** GO TO NEXT SCREEN **
__ Refuse to Answer

If INS4 is less than 1 and INS4 is not equal to 98 then Please choose at least one response from the list. and skip to INS4.

If INS4X is not equal to 1 then Please hit ** GO TO NEXT SCREEN ** button for continued list. and skip to INS4.

INS5. *(Continued from previous screen)* In the past 6-months, did you attempt to get any of the following services, but were told that the agency providing the service is now closed or that the service was no longer being offered? (Check all that apply)

[Note: We are asking about services that you used to get and stopped getting in the past 6 months.] (Check all that apply)

__ Drop-in center such as Ladies Night
__ Job Search Center
__ Violence or trauma recovery services
__ Domestic Violence Shelter or support
__ Legal Assistance
__ Meal Program
__ Transportation assistance
__ Another service not listed
__ None of these
__ Refuse to Answer

If INS5 is less than 1 and INS4 is less than 2 and INS5 is not equal to 98 then Please choose at least one response from the list. and skip to INS5.

If INS5X is equal to 1 and (INS4 is greater than 1 or INS5 is greater than 1) then You have selected both "none of these" and another response. Please review. and skip to INS4.

If INS4C is not equal to 1 and INS4D is not equal to 1 and INS4E is not equal to 1 and INS4F is not equal to 1 and INS4G is not equal to 1 and INS4H is not equal to 1 and INS4I is not equal to 1 and INS4J is not equal to 1, then skip to instruction before INS7.

INS6. Have you been able to replace all of the health-related services or medication that you lost?

1 Yes
0 No
8 Refuse to Answer

If INS4G is not equal to 1 and INS4F is not equal to 1 and INS4J is not equal to 1, then skip to instruction before INS8.

INS7. After you lost your prescription medication(s), did you do any of the following? (Check all that apply) (Check all that apply)

__ Find another location for care
__ Make more visits to the emergency room
__ Use other drugs that were not prescribed for you
__ Use other drugs that were bought on the street (heroin, crack, methamphetamine)
__ Drink more alcohol
__ None of these
__ Refuse to Answer

If INS7 is greater than 1 and INS7F is equal to 1 then You have selected both "none of these" and another response. Please review. and skip to INS7.

If INS7 is less than 1 and INS7 is not equal to 98 then Please choose at least one response from the list. and skip to INS7.

If INS4E is not equal to 1 and INS4I is not equal to 1, then skip to instruction before SLF1.

INS8. After you lost health-related services, did you do any of the following? (Check all that apply) (Check all that apply)

__ Find another location for care
__ Make more visits to the emergency room
__ Use other drugs that were not prescribed for you
__ Use other drugs that were bought on the street (heroin, crack, methamphetamine
__ Drink more alcohol
__ None of these
__ Refuse to Answer

If INS8 is greater than 1 and INS8F is equal to 1 then You have selected both "none of these" and another response. Please review. and skip to INS8.

If INS8 is less than 1 and INS8 is not equal to 98 then Please choose at least one response from the list. and skip to INS8.

SELF-ESTEEM

Next is a list of statements dealing with your general feelings about yourself. Please tell me if you strongly agree, agree, have mixed feelings, disagree, or strongly disagree with each statement.

SLF1. On the whole, I am happy with myself. (Choose one)

1 Strongly disagree
2 Disagree
3 Mixed
4 Agree
5 Strongly agree
8 Refuse to Answer

SLF2. At times, I think I am no good at all. (Choose one)

1 Strongly disagree
2 Disagree
3 Mixed
4 Agree
5 Strongly agree
8 Refuse to Answer

SLF3. I feel that I have a number of good qualities. (Choose one)

1 Strongly disagree
2 Disagree
3 Mixed
4 Agree
5 Strongly agree
8 Refuse to Answer

SLF4. I am able to do things as well as most other people. (Choose one)

1 Strongly disagree
2 Disagree
3 Mixed
4 Agree
5 Strongly agree
8 Refuse to Answer

SLF5. I feel I do not have much to be proud of. (Choose one)

1 Strongly disagree
2 Disagree
3 Mixed
4 Agree
5 Strongly agree
8 Refuse to Answer

SLF6. I certainly feel useless at times. (Choose one)

1	Strongly disagree
2	Disagree
3	Mixed
4	Agree
5	Strongly agree
8	Refuse to Answer

SLF7. I feel that I'm a person of worth, at least as worthy as others. (Choose one)

1	Strongly disagree
2	Disagree
3	Mixed
4	Agree
5	Strongly agree
8	Refuse to Answer

SLF8. I wish I could have more respect for myself. (Choose one)

1	Strongly disagree
2	Disagree
3	Mixed
4	Agree
5	Strongly agree
8	Refuse to Answer

SLF9. All in all, I feel that I am a failure. (Choose one)

1	Strongly disagree
2	Disagree
3	Mixed
4	Agree
5	Strongly agree
8	Refuse to Answer

SLF10. I take a positive attitude toward myself. (Choose one)

1	Strongly disagree
2	Disagree
3	Mixed
4	Agree
5	Strongly agree
8	Refuse to Answer

RESILIENCY

Next, I am going to ask you questions about how you bounce back after difficult times. Please tell me if you strongly agree, agree, have mixed feelings, disagree, or strongly disagree with each statement.

RSL1. I usually manage to get by one way or another. (Choose one)

1 Strongly disagree
2 Disagree
3 Mixed
4 Agree
5 Strongly agree
8 Refuse to Answer

RSL2. I feel proud that I have accomplished things in my life. (Choose one)

1 Strongly disagree
2 Disagree
3 Mixed
4 Agree
5 Strongly agree
8 Refuse to Answer

RSL3. I usually handle things that come my way. (Choose one)

1 Strongly disagree
2 Disagree
3 Mixed
4 Agree
5 Strongly agree
8 Refuse to Answer

RSL4. I am friends with myself. (Choose one)

1 Strongly disagree
2 Disagree
3 Mixed
4 Agree
5 Strongly agree
8 Refuse to Answer

RSL5. I am determined or strong-minded. (Choose one)

1 Strongly disagree
2 Disagree
3 Mixed
4 Agree
5 Strongly agree
8 Refuse to Answer

RSL6. I stay interested in things. (Choose one)

1 Strongly disagree
2 Disagree
3 Mixed
4 Agree
5 Strongly agree
8 Refuse to Answer

RSL7. My belief in myself gets me through hard times. (Choose one)

1 Strongly disagree
2 Disagree
3 Mixed
4 Agree
5 Strongly agree
8 Refuse to Answer

RSL8. My life has meaning. (Choose one)

1 Strongly disagree
2 Disagree
3 Mixed
4 Agree
5 Strongly agree
8 Refuse to Answer

RSL9. When I am in a difficult situation, I can usually find my way out of it. (Choose one)

1 Strongly disagree
2 Disagree
3 Mixed
4 Agree
5 Strongly agree
8 Refuse to Answer

RSL10. I have enough motivation to do what I have to do. (Choose one)

1 Strongly disagree
2 Disagree
3 Mixed
4 Agree
5 Strongly agree
8 Refuse to Answer

SOCIAL ISOLATION

Now I'm going to read you a set of statements about the past six months. Please tell me if you felt that way all of the time, most of the time, some of the time, a little of the time, or not at all.

[Interviewer note: Show Response Card.]

SOC1. In the past six months:

It has been easy to relate to others. (Choose one)

0 Not at all
1 A little of the time
2 Some of the time
3 Most of the time
4 All of the time
8 Refuse to Answer

SOC2. In the past six months:

I felt isolated from other people. (Choose one)

0 Not at all
1 A little of the time
2 Some of the time
3 Most of the time
4 All of the time
8 Refuse to Answer

SOC3. **I had someone to share my feelings with.** (Choose one)

0 Not at all
1 A little of the time
2 Some of the time
3 Most of the time
4 All of the time
8 Refuse to Answer

SOC4. **I found it easy to get in touch with others when I needed to.** (Choose one)

0 Not at all
1 A little of the time
2 Some of the time
3 Most of the time
4 All of the time
8 Refuse to Answer

SOC5. **Even when I'm with other people, I feel separate from them.** (Choose one)

0	Not at all
1	A little of the time
2	Some of the time
3	Most of the time
4	All of the time
8	Refuse to Answer

SOC6. In the past six months:

I felt alone and friendless. (Choose one)

0	Not at all
1	A little of the time
2	Some of the time
3	Most of the time
4	All of the time
8	Refuse to Answer

SOC7. **I spent as much time with other people as I wanted to.** (Choose one)

0	Not at all
1	A little of the time
2	Some of the time
3	Most of the time
4	All of the time
8	Refuse to Answer

SOC8. **Most of the people I spent time with were acquaintances and not true friends.** (Choose one)

0	Not at all
1	A little of the time
2	Some of the time
3	Most of the time
4	All of the time
8	Refuse to Answer

SOC9. **Being with other people meant being in places that I didn't want to be.** (Choose one)

0	Not at all
1	A little of the time
2	Some of the time
3	Most of the time
4	All of the time
8	Refuse to Answer

SOC10. In the past six months:

I felt hustled or used by other people around me. (Choose one)

0	Not at all
1	A little of the time
2	Some of the time
3	Most of the time
4	All of the time
8	Refuse to Answer

SOC11. In the past 6 months, what is the longest period of in which you did not spend time with or visit other people? (Choose one)

0	A few hours
1	One day
2	Three days
3	One week
4	Two or three weeks
5	A month or more
8	Refuse to Answer

If SOC11 is not equal to 2 and SOC11 is not equal to 3 and SOC11 is not equal to 4 and SOC11 is not equal to 5, then skip to SSP1.

Now, I'm going to read a list of reasons you may have spent time alone during the past 6 months. Please answer "yes" or "no".

SOC12. Did you spend time alone in the past 6 months because you ...

Don't have many friends

1	Yes
0	No
8	Refuse to Answer

SOC13. Did you spend time alone in the past 6 months because you ...

Like being by yourself

1	Yes
0	No
8	Refuse to Answer

SOC14. **Were in pain or felt sick**

1	Yes
0	No
8	Refuse to Answer

SOC15. **Were depressed**

1	Yes
0	No
8	Refuse to Answer

SOC16. **Wanted to stay away from dangerous people or situations**

1	Yes
0	No
8	Refuse to Answer

SOC17. Did you spend time alone in the past 6 months because you ...

Wanted to avoid people who use drugs or alcohol

1 Yes
0 No
8 Refuse to Answer

SOC18. **Were too busy working, hustling, or taking care of business**

1 Yes
0 No
8 Refuse to Answer

SOC19. **Don't like other people**

1 Yes
0 No
8 Refuse to Answer

SOC20. **Don't want to talk to other people**

1 Yes
0 No
8 Refuse to Answer

SOC21. Did you spend time alone in the past 6 months because you ...

Have a boyfriend or partner who doesn't like it when you spend time with other people

1 Yes
0 No
7 Don't Know
8 Refuse to Answer
9 Not Applicable

SOC22. **Have another friend or associate who doesn't like it when you spend time with other people**

1 Yes
0 No
8 Refuse to Answer

SOC23. **Have no money to do activities**

1 Yes
0 No
8 Refuse to Answer

SOC24. **Didn't want to be alone, it's just hard to get together with friends**

1 Yes
0 No
8 Refuse to Answer

SOCIAL SUPPORT

SSP1. How many close friends do you have who you can trust to tell personal things?

— — —	friends
998	Refuse to Answer

If SSP1 is equal to 0 or SSP1 is equal to 998, then skip to instruction before SSP8.

SSP2. Of these [Response to SSP1] close friends, how many are female?

— — —	friends
998	Refuse to Answer

If SSP2 is greater than SSP1 then Your response is too high. Please review and skip to instruction before SSP2.

SSP3. Of these [Response to SSP1] close friends, how many use drugs right now?

— — —	friends
998	Refuse to Answer

If SSP3 is greater than SSP1 then Your response is too high. Please review and skip to instruction before SSP3.

If SSP3 is equal to 0 or SSP3 is equal to 998, then skip to instruction before SSP6.

SSP4. Among your friends who use drugs, is there someone who will lend you money or give you money when they have it and you need it?

1	Yes
0	No
8	Refuse to Answer

SSP5. Among the friends who use drugs, is there someone who will take you in when you need a place to stay and they have one to offer?

1	Yes
0	No
8	Refuse to Answer

If SSP3 is equal to SSP1, then skip to instruction before SSP8.

SSP6. Among your friends who do NOT use drugs, is there someone who will lend you money or give you money when they have it and you need it?

1	Yes
0	No
8	Refuse to Answer

SSP7. Among the friends who do NOT use drugs, is there someone who will take you in when you need a place to stay and they have one to offer?

1	Yes
0	No
8	Refuse to Answer

Now I will ask you about other possible sources of social support.

SSP8. Have you talked to, received letters from, or seen any family members during the past 6 months?

1 Yes
0 No
8 Refuse to Answer

If SSP8 is not equal to 1, then skip to SSP11.

SSP9. How often did you talk to a member of your family on the telephone during the past 6 months? (Choose one)

1 **At least once a day**
2 **At least once a week**
3 **At least once a month**
4 **Less than once a month**
5 **Not at all**
8 Refuse to Answer

SSP10. How often did you get together with a member of your family during the past 6 months? (Choose one)

1 **At least once a day**
2 **At least once a week**
3 **At least once a month**
4 **Less than once a month**
5 **Not at all**
8 Refuse to Answer

SSP11. How often did you attend religious services during the past 6 months? (Choose one)

1 Never
2 Once or twice
3 **M**onthly
4 **Weekly**
5 **More than once a week**
8 Refuse to Answer

SSP12. During the past 6 months, how often have you received help or support from a church or religious organization such as food from a soup kitchen, shelter, or clothing? (Choose one) 1 Neve[illegible]

2 Once or twice
3 **M**onthly
4 **Weekly**
5 **More than once a week**
8 Refuse to Answer

INTIMATE/EMOTIONAL PARTNERSHIPS

If Q3 is equal to 1, then skip to instruction before PAR1.

If COUNT8 is not equal to 0, then skip to instruction before PAR1.

[BROKEN8]

If COUNT8 is equal to 0, then skip to end of questionnaire.

If Q4 is equal to 1 or COUNT4 is equal to 1, then skip to instruction before PAR1.

If COUNT6 is not equal to 0, then skip to instruction before PAR1.

[BROKEN6]

If COUNT6 is equal to 0, then skip to end of questionnaire.

If Q5 is equal to 1 or COUNT4 is equal to 1 or COUNT3 is equal to 1, then skip to instruction before PAR1.

If COUNT5 is not equal to 0, then skip to instruction before PAR1.

[BROKEN5]

If COUNT5 is equal to 0, then skip to end of questionnaire.

If COUNT4 is equal to 1 or COUNT3 is equal to 1 or COUNT2 is equal to 1, then skip to instruction before PAR1.

If COUNT7 is not equal to 0, then skip to instruction before PAR1.

[BROKEN7]

If COUNT7 is equal to 0, then skip to end of questionnaire.

Now, I'd like to ask you some questions about people you may feel emotionally close to. I'll call these people intimate partners and I mean someone that you love or feel a special sense of commitment toward, such as a spouse or a serious boyfriend or girlfriend.

If PAR2BL is not equal to 1, then skip to PAR3.

PAR1. Last time we spoke, you mentioned [PINIT1] as your primary intimate partner. Is [PINIT1] still the person you feel closest or most committed to?

1	Yes
0	No
8	Refuse to Answer

If PAR1 is equal to 1, then skip to PAR3.

PAR2. Is that because: (Check all that apply) (Check all that apply)

__	You're no longer with that person
__	Your partner moved away
__	Your partner is in jail/prison
__	Other
__	Refuse to Answer

If PAR2 is less than 1 and PAR2 is not equal to 98 then Please choose at least one response from the list. and skip to PAR2.

PAR3. Have you had any [PARBL]intimate partners within the past 6 months?

1	Yes
0	No
8	Refuse to Answer

If PAR3 is not equal to 1 and (PAR2BL is equal to 0 or PAR1 is not equal to 1), then skip to instruction before PRA1.

If PAR3 is not equal to 1 and PAR2BL is equal to 1, then skip to PAR9.

PAR4. How many intimate partners have you had in the past 6 months?

__ __ __	partners
998	Refuse to Answer

If PAR1 is equal to 1, then skip to PAR9.

PAR5. Is there one primary person who you feel closest or **most committed** to?

1	Yes
0	No
8	Refuse to Answer

If PAR5 is not equal to 1, then skip to instruction before PAR25.

PAR6. What are the initials of the person who you're most committed to? _______ __ __ __ __

PAR7. Is this person a man?

1	Yes
0	No
8	Refuse to Answer

PAR8. About how long have you considered [Response to PAR6] to be your primary intimate partner?

__ __	YEARS
__ __	MONTHS
__ __	DAYS
98	Refuse to Answer (Years)

PAR9. About how often do you spend time with [PINIT4]? (Choose one)

0	Not at all
1	A few times during the past 6 months
2	At least once a month
3	At least once a week
4	At least once a day
8	Refuse to Answer

PAR10. At any time in the past 6 months, did you and [PINIT4] live together?

1	Yes
0	No
8	Refuse to Answer

PAR11. In the last 6 months, has [PINIT4] been in jail or prison?

1	Yes
0	No
7	Don't Know
8	Refuse to Answer

If PAR11 is not equal to 1, then skip to PAR15.

PAR12. Because [Response to PAR6] was incarcerated, did you need to find another place to stay?

1	Yes
0	No
8	Refuse to Answer

PAR13. Because [PINIT4] was incarcerated, did you need to sell sex in order to support yourself?

1	Yes
0	No
8	Refuse to Answer

PAR14. Because [PINIT4] was incarcerated, did you need to sell drugs or steal anything in order to support yourself?

1	Yes
0	No
8	Refuse to Answer

PAR15. In the past 6 months, how often did [PINIT4] give you:

A place to stay?

[Note to interviewer: If R lives with partner and partner pays for the place they stay, answer Every day.] (Choose one)

0 Never in the past 6 months
1 A few times during the past 6 months
2 At least once a month
3 At least once a week
4 Every day
8 Refuse to Answer

PAR16. In the past 6 months, how often did [PINIT4] give you:

Money? (Choose one)

0 Never in the past 6 months
1 A few times during the past 6 months
2 At least once a month
3 At least once a week
4 Every day
8 Refuse to Answer

PAR17. **Drugs?** (Choose one)

0 Never in the past 6 months
1 A few times during the past 6 months
2 At least once a month
3 At least once a week
4 Every day
8 Refuse to Answer

PAR18. **Alcohol?** (Choose one)

0 Never in the past 6 months
1 A few times during the past 6 months
2 At least once a month
3 At least once a week
4 Every day
8 Refuse to Answer

PAR19. **Food?** (Choose one)

0 Never in the past 6 months
1 A few times during the past 6 months
2 At least once a month
3 At least once a week
4 Every day
8 Refuse to Answer

PAR20. In the past 6 months, how often have you given [PINIT4]:

A place to stay? (Choose one)

0	Never in the past 6 months
1	A few times during the past 6 months
2	At least once a month
3	At least once a week
4	Every day
8	Refuse to Answer

PAR21. In the past 6 months, how often have you given [PINIT4]:

Money? (Choose one)

0	Never in the past 6 months
1	A few times during the past 6 months
2	At least once a month
3	At least once a week
4	Every day
8	Refuse to Answer

PAR22. **Drugs?** (Choose one)

0	Never in the past 6 months
1	A few times during the past 6 months
2	At least once a month
3	At least once a week
4	Every day
8	Refuse to Answer

PAR23. **Alcohol?** (Choose one)

0	Never in the past 6 months
1	A few times during the past 6 months
2	At least once a month
3	At least once a week
4	Every day
8	Refuse to Answer

PAR24. **Food?** (Choose one)

0	Never in the past 6 months
1	A few times during the past 6 months
2	At least once a month
3	At least once a week
4	Every day
8	Refuse to Answer

Skip to instruction before PAR25.

Skip to instruction before PRA1.

I'm going to read you a list of statements about how your relationship is with your primary intimate partner now. Choose how much you agree or disagree with each statement. Your answer can be strongly disagree, disagree, have mixed feelings, agree, or strongly agree.

PAR25. I have major interests of my own outside of the relationship. (Choose one)

1 Strongly disagree
2 Disagree
3 Mixed
4 Agree
5 Strongly agree
8 Refuse to Answer

PAR26. I have a supportive group of friends separate from my partner. (Choose one)

1 Strongly disagree
2 Disagree
3 Mixed
4 Agree
5 Strongly agree
8 Refuse to Answer

PAR27. I have a close friend other than my partner. (Choose one)

1 Strongly disagree
2 Disagree
3 Mixed
4 Agree
5 Strongly agree
8 Refuse to Answer

PAR28. My sense of being an individual is separate from my sense of being part of a couple. (Choose one)

1 Strongly disagree
2 Disagree
3 Mixed
4 Agree
5 Strongly agree
8 Refuse to Answer

PAR29. I make most decisions on my own without checking with my partner. (Choose one)

1 Strongly disagree
2 Disagree
3 Mixed
4 Agree
5 Strongly agree
8 Refuse to Answer

PAR30. I maintain the position that if I had to, I could really make it on my own. (Choose one)

1 Strongly disagree
2 Disagree
3 Mixed
4 Agree
5 Strongly agree
8 Refuse to Answer

PAR31. Although I can depend on my partner, the only one I can **really** count on is myself. (Choose one)

1 Strongly disagree
2 Disagree
3 Mixed
4 Agree
5 Strongly agree
8 Refuse to Answer

PAR32. I manage my own money. (Choose one)

1 Strongly disagree
2 Disagree
3 Mixed
4 Agree
5 Strongly agree
8 Refuse to Answer

Now, I'm going to read you a list of statements about how equal you think you are with your partner. Choose how much this is true or not true for each statement. Your answer can be: Not at all true, rarely true, somewhat true, true, or extremely true.

PAR33. My partner and I have equal power in the relationship. (Choose one)

1 Not at all true
2 Rarely true
3 Somewhat true
4 True
5 Extremely true
8 Refuse to Answer

PAR34. My partner shows as much affection to me as I think I show to my partner. (Choose one)

1 Not at all true
2 Rarely true
3 Somewhat true
4 True
5 Extremely true
8 Refuse to Answer

PAR35. My partner and I invest equal amounts of time and energy in the relationship. (Choose one)

1 Not at all true
2 Rarely true
3 Somewhat true
4 True
5 Extremely true
8 Refuse to Answer

PAR36. My partner and I are equally committed to working out problems that occur in our relationship. (Choose one)

1 Not at all true
2 Rarely true
3 Somewhat true
4 True
5 Extremely true
8 Refuse to Answer

PAR37. All things considered, my partner and I contribute an equal amount to the relationship. (Choose one)

1 Not at all true
2 Rarely true
3 Somewhat true
4 True
5 Extremely true
8 Refuse to Answer

PAR38. My partner and I deal with each other as equals. (Choose one)

1 Not at all true
2 Rarely true
3 Somewhat true
4 True
5 Extremely true
8 Refuse to Answer

PAR39. My partner treats me and respects me as an equal. (Choose one)

1 Not at all true
2 Rarely true
3 Somewhat true
4 True
5 Extremely true
8 Refuse to Answer

PAR40. My partner depends on me as much as I depend on him. (Choose one)

1	Not at all true
2	Rarely true
3	Somewhat true
4	True
5	Extremely true
8	Refuse to Answer

PAR41. When we have to make an important decision, we make it as equals. (Choose one)

1	Not at all true
2	Rarely true
3	Somewhat true
4	True
5	Extremely true
8	Refuse to Answer

[Interviewer note: ACASI section starts. Please read the following instructions and give computer and headphones to R.]

We're making good progress. I really appreciate how much effort you're putting into answering the questions. For the next part of the interview, I'm going to turn the computer over to you so that you can answer the questions yourself. We have these headphones so that you can listen to the questions in privacy, and you can also read the questions on the computer screen.

You will use the pen to enter the answers yourself. The first few questions are for practice, just to get you familiar with how this will work. If you are unsure about how to answer any of the questions, I'll be right here so just let me know.

PRACTICE SECTION

Let's begin the practice questions. Sometimes you will hear a natural human voice,

and sometimes you will hear a computerized voice.

The following examples will give you an idea of the types of questions you will see during the interview, and will give you a chance to ask your interviewer any questions before you get started.

PRA1. Some of the questions will ask you to answer either YES or NO. For example, "Are you wearing a green shirt today?"

1 Yes
0 No
9 Refuse to Answer

PRA2. Some of the questions will ask you to pick one answer from a list, such as, How much do you agree with this statement? Blue is my favorite color. (Choose one) (Choose one)

__ Stron
__ Disagree
__ Neither Agree Nor Disagree
__ Agree
__ Strongly Agree
__ Refuse to Answer

PRA3. Some of the questions will ask you to pick one or more answers from a list of choices, such as, what colors do you like? (Check all that apply) (Check all that apply)

__ Red
__ Blue
__ Green
__ Yellow
__ Purple
__ Refuse to Answer

If PRA3 is less than 1 and PRA3 is not equal to 98 then Please choose at least one response from the list. and skip to PRA3.

PRA4. Has this been enough practice using the computer? If it has, click YES and you can get started. If not, click NO and you will get a few more practice questions.

1 Yes
0 No
9 Refuse to Answer

If PRA4 is equal to 1, then skip to instruction before SEX1.

Try a few more questions to practice getting used to the computer...

PRA5. Do you like watching TV?

1 Yes
0 No
9 Refuse to Answer

PRA6. How much do you agree with the following statement, My favorite thing about TV is watching sports. (Choose one) (Choose one)

- __ Strongly Disagree
- __ Disagree
- __ Neither Agree Nor Disagree
- __ Agree
- __ Strongly Agree
- __ Refuse to Answer

PRA7. The things I don't like about living in a big city are ... (Check all that apply) (Check all that apply)

- __ Crowds
- __ Weather
- __ Cars
- __ Cost
- __ Other things not listed here
- __ Refuse to Answer

If PRA7 is less than 1 and PRA7 is not equal to 98 then Please choose at least one response from the list. and skip to PRA7.

Let's change gears a bit and ask some more interview questions.

SEXUAL ACTIVITIES

Now I'd like to ask you some questions about people you have sex with including boyfriends or girlfriends, casual partners, one-night stands, and people who paid to have sex with you. By sex, I mean a penis, mouth, hand or object coming into contact with a vagina or butt. I also mean a mouth coming into contact with a penis.

SEX1. How many sex partners have you had in the past 6 months?

__ __ __ sex partners
998 Refuse to Answer

If SEX1 is equal to 0, then skip to instruction before SEX23.

If SEX1 is equal to 1, then skip to SEX6.

SEX2. How many of those [Response to SEX1] sex partners were men?

__ __ __ men
998 Refuse to Answer

If SEX2 is greater than SEX1 then You have entered a number that is greater than the number of sex partners you had in the past 6 months. Please review your answer and skip to SEX2.

If SEX2 is equal to 0, then skip to instruction before SEX23.

If SEX2 is equal to 1, then skip to SEX7.

SEX3. First I want to know about vaginal sex. When I say vaginal sex, I mean when a man puts his penis into your vagina. How many of those [Response to SEX2] partners did you have vaginal sex with? __ __ __men
998 Refuse to Answer

If SEX3 is greater than SEX2 then You have entered a number that is greater than the number of male sex partners you had in the past 6 months. Please review your answer. and skip to SEX3.

SEX4. Now I want to know about anal sex. When I say anal sex, I mean when a man puts his penis into your butt. How many of those [Response to SEX2] partners did you have anal sex with? __ __ __men
998 Refuse to Answer

If SEX4 is greater than SEX2 then You have entered a number that is greater than the number of male sex partners you had in the past 6 months. Please review your answer. and skip to SEX4.

SEX5. Now I want to know about oral sex. When I say oral sex, I mean when a man puts his tongue or mouth in or around your vagina; OR when you put your mouth or tongue on or around his penis. How many of those [Response to SEX2] partners did you have oral sex with?

__ __ __ men
998 Refuse to Answer

If SEX5 is greater than SEX2 then You have entered a number that is greater than the number of male sex partners you had in the past 6 months. Please review your answer. and skip to SEX5.

If (SEX3 is equal to 0 or SEX3 is equal to 998) and (SEX4 is equal to 0 or SEX4 is equal to 998), then skip to instruction before SEX20.

Skip to SEX10.

SEX6. Was this sex partner a man?

1 Yes
0 No
8 Refuse to Answer

If SEX6 is not equal to 1, then skip to instruction before SEX23.

SEX7. First I want to know about vaginal sex. When I say vaginal sex, I mean when a man puts his penis into your vagina. Did you have vaginal sex with this partner?

1 Yes
0 No
8 Refuse to Answer

SEX8. Now I want to know about anal sex. When I say anal sex, I mean when a man puts his penis into your butt. Did you have anal sex with this partner?

1 Yes
0 No
8 Refuse to Answer

SEX9. Now I want to know about oral sex. When I say oral sex, I mean when a man puts his tongue or mouth in or around your vagina; OR when you put your mouth or tongue on or around his penis. Did you have oral sex with this partner?

1 Yes
0 No
8 Refuse to Answer

If SEX7 is not equal to 1 and SEX8 is not equal to 1, then skip to instruction before SEX20.

SEX10. Thinking about vaginal and anal sex only, were there times when you did not use a condom during the past 6 months?

1 Yes
0 No
8 Refuse to Answer

If SEX10 is not equal to 1, then skip to SEX18.

If SEX1 is equal to 1 or SEX2 is equal to 1, then skip to SEX12.

SEX11. During the past 6 months, how many partners did you have vaginal or anal sex with and not use a condom?

__ __ __ men
998 Refuse to Answer

If SEX11 is greater than SEX2 then You have entered a number that is greater than the number of male sex partners you had in the past 6 months. Please review your answer. and skip to SEX11.

SEX12. Thinking about men who you had vaginal or anal sex with in the past 6 months, were any of these people...? (Check all that apply) (Check all that apply) __ Prim

- __ Casual partners or someone you have sex with from time to time, but are not committed to
- __ One night stands
- __ Paying clients
- __ Other
- __ Refuse to Answer

If SEX12 is less than 1 and SEX12 is not equal to 98 then Please choose at least one response from the list. and skip to SEX12.

If SEX12A is equal to 1 and PAR5 is not equal to 1 and PAR1 is not equal to 1 then Earlier, you said that you did not have a primary intimate partner within the past 6 months. Please review your response. and skip to SEX12.

If SEX12 is greater than 1 and SEX2 is equal to 1 then Earlier, you said that you only had one partner in the past 6 months. Please review your response. and skip to SEX12.

If SEX12A is not equal to 1, then skip to instruction before SEX14.

SEX13. What are some of the reasons why you had sex without a condom with your primary intimate partner? (Check all that apply) (Check all that apply)

- __ It was a long-term partner
- __ I love and trust the person to keep me safe
- __ We are monogamous
- __ The person does not have any infections
- __ We had anal sex, so my risks were low
- __ I was not worried about getting pregnant
- __ It was too much of a bother to use a condom
- __ We did not have a condom
- __ I was embarrassed to suggest a condom
- __ The person asked me not to use a condom
- __ The person refused to wear a condom
- __ The person scared me and I did not suggest a condom
- __ This was a job and I could get more money without a condom
- __ The condom slipped off accidentally or broke
- __ I was drunk or high
- __ I didn't care
- __ I wanted to get infected
- __ The type of sex we had did not require a condom
- __ I clean myself afterwards and did not need a condom
- __ I take certain medications and don't need to use condoms
- __ We used a different type of barrier method
- __ We both have the same infections
- __ I did not want to use a condom
- __ Refuse to Answer

If SEX13 is less than 1 and SEX13 is not equal to 98 then Please choose at least one response from the list. and skip to SEX13.

If SEX12B is not equal to 1, then skip to instruction before SEX15.

SEX14. When you had sex with a casual partner, what are some of the reasons why you had sex without a condom? (Check all that apply) (Check all that apply) __ It wa

__ I love and trust the person to keep me safe
__ We are monogamous
__ The person does not have any infections
__ We had anal sex, so my risks were low
__ I was not worried about getting pregnant
__ It was too much of a bother to use a condom
__ We did not have a condom
__ I was embarrassed to suggest a condom
__ The person asked me not to use a condom
__ The person refused to wear a condom
__ The person scared me and I did not suggest a condom
__ This was a job and I could get more money without a condom
__ The condom slipped off accidentally or broke
__ I was drunk or high
__ I didn't care
__ I wanted to get infected
__ The type of sex we had did not require a condom
__ I clean myself afterwards and did not need a condom
__ I take certain medications and don't need to use condoms
__ We used a different type of barrier method
__ We both have the same infections
__ I did not want to use a condom
__ Refuse to Answer

If SEX14 is less than 1 and SEX14 is not equal to 98 then Please choose at least one response from the list. and skip to SEX14.

If SEX12C is not equal to 1, then skip to instruction before SEX16.

SEX15. When you had a one-night stand, what are some of the reasons why you had sex without a condom? (Check all that apply) (Check all that apply)

__ I love and trust the person to keep me safe
__ The person does not have any infections
__ We had anal sex, so my risks were low
__ I was not worried about getting pregnant
__ It was too much of a bother to use a condom
__ We did not have a condom
__ I was embarrassed to suggest a condom
__ The person asked me not to use a condom
__ The person refused to wear a condom
__ The person scared me and I did not suggest a condom
__ This was a job and I could get more money without a condom
__ The condom slipped off accidentally or broke
__ I was drunk or high
__ I didn't care
__ I wanted to get infected
__ The type of sex we had did not require a condom
__ I clean myself afterwards and did not need a condom
__ I take certain medications and don't need to use condoms
__ We used a different type of barrier method
__ We both have the same infections
__ I did not want to use a condom
__ Refuse to Answer

If SEX15 is less than 1 and SEX15 is not equal to 98 then Please choose at least one response from the list. and skip to SEX15.

If SEX12D is not equal to 1, then skip to instruction before SEX17.

SEX16. When you had sex with a paying client, what are some of the reasons why you had sex without a condom? (Check all that apply) (Check all that apply) __ I lov

- __ The person does not have any infections
- __ We had anal sex, so my risks were low
- __ I was not worried about getting pregnant
- __ It was too much of a bother to use a condom
- __ We did not have a condom
- __ I was embarrassed to suggest a condom
- __ The person asked me not to use a condom
- __ The person refused to wear a condom
- __ The person scared me and I did not suggest a condom
- __ This was a job and I could get more money without a condom
- __ The condom slipped off accidentally or broke
- __ I was drunk or high
- __ I didn't care
- __ I wanted to get infected
- __ The type of sex we had did not require a condom
- __ I clean myself afterwards and did not need a condom
- __ I take certain medications and don't need to use condoms
- __ We used a different type of barrier method
- __ We both have the same infections
- __ I did not want to use a condom
- __ Refuse to Answer

If SEX16 is less than 1 and SEX16 is not equal to 98 then Please choose at least one response from the list. and skip to SEX16.

If SEX12E is not equal to 1, then skip to SEX18.

SEX17. When you had sex with an "other" type of sex partner, what are some of the reasons why you had sex without a condom? (Check all that apply) (Check all that apply) __ It wa

- __ I love and trust the person to keep me safe
- __ We are monogamous
- __ The person does not have any infections
- __ We had anal sex, so my risks were low
- __ I was not worried about getting pregnant
- __ It was too much of a bother to use a condom
- __ We did not have a condom
- __ I was embarrassed to suggest a condom
- __ The person asked me not to use a condom
- __ The person refused to wear a condom
- __ The person scared me and I did not suggest a condom
- __ This was a job and I could get more money without a condom
- __ The condom slipped off accidentally or broke
- __ I was drunk or high
- __ I didn't care
- __ I wanted to get infected
- __ The type of sex we had did not require a condom
- __ I clean myself afterwards and did not need a condom
- __ I take certain medications and don't need to use condoms
- __ We used a different type of barrier method
- __ We both have the same infections
- __ I did not want to use a condom
- __ Refuse to Answer

If SEX17 is less than 1 and SEX17 is not equal to 98 then Please choose at least one response from the list. and skip to SEX17.

SEX18. Have you used any other type of birth control or contraception other than condoms in the past 6 months?

1	Yes
0	No
8	Refuse to Answer

Now, I have some other questions about your sex partners.

SEX19. In the past six months, have you had group sex or sex with more than one person at a time?

1	Yes
0	No
8	Refuse to Answer

If SEX3 is not greater than 0 and SEX4 is not greater than 0, then skip to instruction before SEX22.

SEX20. As far as you know, have any of the people that you had vaginal or anal sex with in the past 6 months been **HIV-positive**?

1 Yes
0 No
8 Refuse to Answer

SEX21. As far as you know, have any of the people that you had vaginal or anal sex with in the past 6 months been **HIV-negative**?

1 Yes
0 No
8 Refuse to Answer

If SEX7 is not equal to 1 and SEX8 is not equal to 1, then skip to instruction before SEX23.

SEX22. As far as you know, is the person that you had vaginal or anal sex with in the past 6 months HIV-positive?

1 Yes
0 No
8 Refuse to Answer

If COUNT12 is not equal to 0, then skip to instruction before SEX23.

[BROKEN12]

If COUNT12 is equal to 0, then skip to end of questionnaire.

SEX EXCHANGE

Next, I'm going to ask some questions about having sex in exchange for money, drugs, or something else.

If LSEXT is equal to 1, then skip to SEX24.

SEX23. Thinking about your whole life, have you ever had sex in exchange for money, drugs, housing, food, or protection?

1 Yes
0 No
8 Refuse to Answer

If SEX23 is not equal to 1, then skip to instruction before VIC1.

SEX24. In the past 6 months, have you had sex in exchange for money, drugs, housing, food, or protection?

1 Yes
0 No
8 Refuse to Answer

If SEX24 is not equal to 1, then skip to instruction before VIC1.

SEX25. In the past 6 months, did you have sex in exchange for a **place** to sleep?

1 Yes
0 No
8 Refuse to Answer

SEX26. (In the past 6 months) did you have sex in exchange for **money**?

1 Yes
0 No
8 Refuse to Answer

SEX27. (In the past 6 months) did you have sex in exchange for **drugs**?

1 Yes
0 No
8 Refuse to Answer

SEX28. (In the past 6 months) did you have sex in exchange for **protection from violence**?

1 Yes
0 No
8 Refuse to Answer

SEX29. (In the past 6 months) did you have sex in exchange for **food**?

1 Yes
0 No
8 Refuse to Answer

SEX30. (In the past 6 months) did you have sex in exchange for **anything else**?

1 Yes
0 No
8 Refuse to Answer

SEX31. For those times in the past 6 months that someone gave you something in exchange for sex, how often did this happen? (Choose one)

1 once or twice in the past 6 months
2 at least once a month
3 more than once a month
4 at least once a week
8 Refuse to Answer

SEX32. Is there anyone like a boyfriend or pimp you who decides how much you earn when you are exchanging sex?

1 Yes
0 No
8 Refuse to Answer

SEX33. Is there anyone who decides where or when you can exchange sex?

1 Yes
0 No
8 Refuse to Answer

SEX34. Is there anyone who keeps some of your money when exchanging sex?

1 Yes
0 No
8 Refuse to Answer

SEX35. Is there anyone else like a hotel manager who makes money when you exchange sex ?

1 Yes
0 No
8 Refuse to Answer

SEX36. In the past 6 months, have you ever exchanged sex on the streets?

1 Yes
0 No
8 Refuse to Answer

If SEX36 is not equal to 1, then skip to SEX38.

SEX37. Have you been attacked when working on the streets? By attacked, I mean someone hit, punched, kicked, tackled, raped, or used a weapon on you. (Choose one) 0 Neve[illegible]

1 A few times during the past 6 months
2 At least once a month
3 At least once a week
4 Every day
8 Refuse to Answer

SEX38. In the past 6 months, have you ever exchanged sex in a car?

1 Yes
0 No
8 Refuse to Answer

If SEX38 is not equal to 1, then skip to SEX40.

SEX39. In the past 6 months, have you been attacked when exchanging sex in a car? By attacked, I mean someone hit, punched, kicked, tackled, raped, or used a weapon on you (Choose one) 0 Neve[illegible]

1 A few times during the past 6 months
2 At least once a month
3 At least once a week
4 Every day
8 Refuse to Answer

SEX40. In the past 6 months, have you ever exchanged sex out of an SRO?

1 Yes
0 No
8 Refuse to Answer

If SEX40 is not equal to 1, then skip to instruction before VIC1.

SEX41. In the past 6 months, have you been attacked when exchanging sex in an SRO? By attacked, I mean someone hit, punched, kicked, tackled, raped, or used a weapon on you. (Choose one) 0 Neve[illegible]

1 A few times during the past 6 months
2 At least once a month
3 At least once a week
4 Every day
8 Refuse to Answer

VIOLENCE AND VICTIMIZATION

Now, I'm going to ask you some questions about violence you may have experienced. Click on each box that applies to you.

If PAR5 is not equal to 1 and PAR1 is not equal to 1, then skip to VIC2.

VIC1. In the past 6 months, did your primary intimate partner [PINIT4] ever (Check all that apply) (Check all that apply)

__ Yell at you or call you names?
__ Say mean or cruel things?
__ Make unwanted sexual advances or sexually harass you?
__ Push, slap, hit, or beat you?
__ Kick, stomp on, or bite you?
__ Choke or attempt to drown you?
__ Threaten to damage things or someone you care about?
__ Damage or destroy things?
__ Hurt someone you care about?
__ Threaten to hurt or kill you?
__ Use a gun, knife, other weapon or object on you?
__ Threaten to kill themselves because of you?
__ Force you to have any kind of sex you didn't want to have or sexually abuse you?
__ None of these
__ Refuse to Answer

If VIC1 is greater than 1 and VIC1N is equal to 1 then You have selected both "none of these" and another response. Please review. and skip to VIC1.

If VIC1 is less than 1 and VIC1 is not equal to 98 then Please choose at least one response from the list. and skip to VIC1.

VIC2. In the past 6 months, did any other adult, male or female, ever . . . (Check all that apply) (Check all that apply)

- __ Yell at you or call you names?
- __ Say mean or cruel things?
- __ Make unwanted sexual advances or sexually harass you?
- __ Push, slap, hit, or beat you?
- __ Kick, stomp on, or bite you?
- __ Choke or attempt to drown you?
- __ Threaten to damage things or someone you care about?
- __ Damage or destroy things?
- __ Hurt someone you care about?
- __ Threaten to hurt or kill you?
- __ Use a gun, knife, other weapon or object on you?
- __ Threaten to kill themselves because of you?
- __ Force you to have any kind of sex you didn't want to have or sexually abuse you?
- __ None of these
- __ Refuse to Answer

If VIC2 is greater than 1 and VIC2N is equal to 1 then You have selected both "none of these" and another response. Please review. and skip to VIC2.

If VIC2 is less than 1 and VIC2 is not equal to 98 then Please choose at least one response from the list. and skip to VIC2.

If (PAR5 is equal to 1 or PAR1 is equal to 1) and VIC2 is equal to 1 and VIC2N is equal to 1 and VIC1 is equal to 1 and VIC1N is equal to 1 or PAR5 is not equal to 1 and PAR1 is not equal to 1 and VIC2 is equal to 1 and VIC2N is equal to 1, then skip to VIC20.

VIC3. Thinking about all the violent or threatening things that happened to you in the past 6 months, did any of them happen to you . . . (Check all that apply) (Check all that apply)

- __ On t
- __ In your room or apartment?
- __ In someone else's room or apartment?
- __ In a bar, restaurant, or other public place?
- __ In a car, truck, or van?
- __ In jail or prison?
- __ In a homeless shelter
- __ Somewhere else
- __ Refuse to Answer

If VIC3 is less than 1 and VIC3 is not equal to 98 then Please choose at least one response from the list. and skip to VIC3.

VIC4. In the past 6 months, were any of the violent or threatening things done by . . . (Check all that apply) (Check all that apply)

__ Your intimate partner &[PINIT4]
__ Someone who used to be your spouse, boyfriend, girlfriend, or intimate partner, but is not anymore
__ Someone you're having sex with, who you don't consider to be an intimate partner
__ A person who makes money from your work, like a pimp or a middleman
__ A person who was doing business with you, like a john or someone else buying something from you
__ A hotel manager or other hotel employee
__ A police officer
__ A neighbor or other resident
__ Someone you use drugs with that you haven't already listed
__ A roommate
__ A friend that you haven't already listed
__ A relative
__ Someone else you knew
__ A stranger
__ **Someone who was getting back at you for something they thought you had done**
__ **Someone involved in a drug-related situation like running or dealing drugs**
__ Refuse to Answer

If VIC4 is less than 1 and VIC4 is not equal to 98 then Please choose at least one response from the list. and skip to VIC4.

If VIC4A is equal to 1 and PAR5 is not equal to 1 and PAR1 is not equal to 1 then You reported not having a primary partner in the past 6 months. Please review your response. and skip to VIC4.

If VIC4B is not equal to 1, then skip to instruction before VIC6.

VIC5. Is the person who used to be your spouse, boyfriend, girlfriend, or intimate partner a man or a woman? **(Please choose "both" if you experienced violence from both a man and a woman who fit into this category)** (Choose one)

1 Man
2 Woman
3 Both
8 Refuse to Answer

If VIC4C is not equal to 1, then skip to instruction before VIC7.

VIC6. Is the person who you're having sex with a man or a woman? **(Please choose "both" if you experienced violence from both a man and a woman who fit into this category)** (Choose one) 1 Man

2 Woman
3 Both
8 Refuse to Answer

If VIC4D is not equal to 1, then skip to instruction before VIC8.

VIC7. Is the person who makes money from your work a man or a woman? **(Please choose "both" if you experienced violence from both a man and a woman who fit into this category)** (Choose one)

1 Man
2 Woman
3 Both
8 Refuse to Answer

If VIC4E is not equal to 1, then skip to instruction before VIC9.

VIC8. Was the person who was doing business with you a man or a woman? **(Please choose "both" if you experienced violence from both a man and a woman who fit into this category)** (Choose one)

1 Man
2 Woman
3 Both
8 Refuse to Answer

If VIC4F is not equal to 1, then skip to instruction before VIC10.

VIC9. Was the hotel manager or other hotel employee a man or a woman? **(Please choose "both" if you experienced violence from both a man and a woman who fit into this category)** (Choose one)

1 Man
2 Woman
3 Both
8 Refuse to Answer

If VIC4G is not equal to 1, then skip to instruction before VIC11.

VIC10. Was the police officer a man or a woman? **(Please choose "both" if you experienced violence from both a man and a woman who fit into this category)** (Choose one)

1 Man
2 Woman
3 Both
8 Refuse to Answer

If VIC4H is not equal to 1, then skip to instruction before VIC12.

VIC11. Was the neighbor or other resident a man or a woman? **(Please choose "both" if you experienced violence from both a man and a woman who fit into this category)** (Choose one)

1 Man
2 Woman
3 Both
8 Refuse to Answer

If VIC4I is not equal to 1, then skip to instruction before VIC13.

VIC12. Was the person who you used to do drugs with a man or a woman? **(Please choose "both" if you experienced violence from both a man and a woman who fit into this category)** (Choose one)

1 Man
2 Woman
3 Both
8 Refuse to Answer

If VIC4J is not equal to 1, then skip to instruction before VIC14.

VIC13. Was your roommate a man or a woman? **(Please choose "both" if you experienced violence from both a man and a woman who fit into this category)** (Choose one)
1 Man
2 Woman
3 Both
8 Refuse to Answer

If VIC4K is not equal to 1, then skip to instruction before VIC15.

VIC14. Was the friend a man or a woman? **(Please choose "both" if you experienced violence from both a man and a woman who fit into this category)** (Choose one)
1 Man
2 Woman
3 Both
8 Refuse to Answer

If VIC4L is not equal to 1, then skip to instruction before VIC16.

VIC15. Is the relative a man or a woman? **(Please choose "both" if you experienced violence from both a man and a woman who fit into this category)** (Choose one)
1 Man
2 Woman
3 Both
8 Refuse to Answer

If VIC4M is not equal to 1, then skip to instruction before VIC17.

VIC16. Was the someone else a man or a woman? **(Please choose "both" if you experienced violence from both a man and a woman who fit into this category)** (Choose one)
1 Man
2 Woman
3 Both
8 Refuse to Answer

If VIC4N is not equal to 1, then skip to instruction before VIC18.

VIC17. Was the stranger a man or a woman? **(Please choose "both" if you experienced violence from both a man and a woman who fit into this category)** (Choose one)
1 Man
2 Woman
3 Both
8 Refuse to Answer

If VIC4O is not equal to 1, then skip to instruction before VIC19.

VIC18. Was the person who was getting back at you a man or a woman? **(Please choose "both" if you experienced violence from both a man and a woman who fit into this category)** (Choose one)
1 Man
2 Woman
3 Both
8 Refuse to Answer

If VIC4P is not equal to 1, then skip to VIC20.

VIC19. Was the person who was involved in a drug-related situation a man or a woman? **(Please choose "both" if you experienced violence from both a man and a woman who fit into this category)** (Choose one)

1	Man
2	Woman
3	Both
8	Refuse to Answer

VIC20. Now, thinking about things that **<u>you</u>** may have done to others:

During the past 6 months, did you ever: (Check all that apply) (Check all that apply)

__ Push, slap, hit, or beat someone?
__ Kick, stomp on, or bite someone?
__ Choke or attempt to drown someone?
__ Threaten to damage things someone cared about?
__ Hit someone with an object?
__ Damage or destroy things someone else cared about?
__ Hurt someone that someone else cared about?
__ Threaten to hurt or kill someone?
__ Use a gun, knife, or other weapon on someone?
__ Threaten to kill yourself because of them?
__ Force someone to have any kind of sex they didn't want to have or sexually abuse someone?
__ None of these
__ Refuse to Answer

If VIC20 is greater than 1 and VIC20X is equal to 1 then You have selected both "none of these" and another response. Please review. and skip to VIC20.

If VIC20 is less than 1 and VIC20 is not equal to 98 then Please choose at least one response from the list. and skip to VIC20.

If VIC20A is not equal to 1 and VIC20B is not equal to 1 and VIC20C is not equal to 1 and VIC20D is not equal to 1 and VIC20E is not equal to 1 and VIC20F is not equal to 1 and VIC20H is not equal to 1 and VIC20I is not equal to 1 and VIC20J is not equal to 1 and VIC20K is not equal to 1 and VIC20L is not equal to 1, then skip to VIC37.

VIC21. In the past 6 months, were any of these aggressive things done to any of the following: (Check all that apply) (Check all that apply)

- __ Your intimate partner &[PINIT4]
- __ Someone who used to be your spouse, boyfriend, girlfriend, or intimate partner, but is not anymore
- __ Someone you're having sex with, who you don't consider to be an intimate partner
- __ A person who makes money from your work, like a pimp or a middleman
- __ A person who was doing business with you, like a john or someone else buying something from you
- __ A hotel manager or other hotel employee
- __ A police officer
- __ A neighbor or other resident
- __ Someone you use drugs with that you haven't already listed
- __ A roommate
- __ A friend that you haven't already listed
- __ A relative
- __ Someone else you knew
- __ A stranger
- __ **Someone who you were getting back at for something you thought they had done**
- __ **Someone involved in a drug-related situation like running or dealing drugs**
- __ Refuse to Answer

If VIC21 is less than 1 and VIC21 is not equal to 98 then Please choose at least one response from the list. and skip to VIC21.

If VIC21A is equal to 1 and PAR5 is not equal to 1 and PAR1 is not equal to 1 then You reported not having a primary partner in the past 6 months. Please review your response. and skip to VIC21.

If VIC21B is not equal to 1, then skip to instruction before VIC23.

VIC22. Is the person who used to be your spouse, boyfriend, girlfriend, or intimate partner a man or a woman? **(Please choose "both" if you experienced violence from both a man and a woman who fit into this category)** (Choose one)

1 Man
2 Woman
3 Both
8 Refuse to Answer

If VIC21C is not equal to 1, then skip to instruction before VIC24.

VIC23. Is the person who you're having sex with a man or a woman? **(Please choose "both" if you experienced violence from both a man and a woman who fit into this category)** (Choose one) 1 Man

2 Woman
3 Both
8 Refuse to Answer

If VIC21D is not equal to 1, then skip to instruction before VIC25.

VIC24. Is the person who makes money from your work a man or a woman? **(Please choose "both" if you experienced violence from both a man and a woman who fit into this category)** (Choose one)

1 Man
2 Woman
3 Both
8 Refuse to Answer

If VIC21E is not equal to 1, then skip to instruction before VIC26.

VIC25. Was the person who was doing business with you a man or a woman? **(Please choose "both" if you experienced violence from both a man and a woman who fit into this category)** (Choose one)

1 Man
2 Woman
3 Both
8 Refuse to Answer

If VIC21F is not equal to 1, then skip to instruction before VIC27.

VIC26. Was the hotel manager or other hotel employee a man or a woman? **(Please choose "both" if you experienced violence from both a man and a woman who fit into this category)** (Choose one)

1 Man
2 Woman
3 Both
8 Refuse to Answer

If VIC21G is not equal to 1, then skip to instruction before VIC28.

VIC27. Was the police officer a man or a woman? **(Please choose "both" if you experienced violence from both a man and a woman who fit into this category)** (Choose one)

1 Man
2 Woman
3 Both
8 Refuse to Answer

If VIC21H is not equal to 1, then skip to instruction before VIC29.

VIC28. Was the neighbor or other resident a man or a woman? **(Please choose "both" if you experienced violence from both a man and a woman who fit into this category)** (Choose one)

1 Man
2 Woman
3 Both
8 Refuse to Answer

If VIC21I is not equal to 1, then skip to instruction before VIC30.

VIC29. Was the person who you used to do drugs with a man or a woman? **(Please choose "both" if you experienced violence from both a man and a woman who fit into this category)** (Choose one)

1 Man
2 Woman
3 Both
8 Refuse to Answer

If VIC21J is not equal to 1, then skip to instruction before VIC31.

VIC30. Was your roommate a man or a woman? **(Please choose "both" if you experienced violence from both a man and a woman who fit into this category)** (Choose one)

1 Man
2 Woman
3 Both
8 Refuse to Answer

If VIC21K is not equal to 1, then skip to instruction before VIC32.

VIC31. Was the friend a man or a woman? **(Please choose "both" if you experienced violence from both a man and a woman who fit into this category)** (Choose one)

1 Man
2 Woman
3 Both
8 Refuse to Answer

If VIC21L is not equal to 1, then skip to instruction before VIC33.

VIC32. Is the relative a man or a woman? **(Please choose "both" if you experienced violence from both a man and a woman who fit into this category)** (Choose one)

1 Man
2 Woman
3 Both
8 Refuse to Answer

If VIC21M is not equal to 1, then skip to instruction before VIC34.

VIC33. Was the someone else a man or a woman? **(Please choose "both" if you experienced violence from both a man and a woman who fit into this category)** (Choose one)

1 Man
2 Woman
3 Both
8 Refuse to Answer

If VIC21N is not equal to 1, then skip to instruction before VIC35.

VIC34. Was the stranger a man or a woman? **(Please choose "both" if you experienced violence from both a man and a woman who fit into this category)** (Choose one)

1 Man
2 Woman
3 Both
8 Refuse to Answer

If VIC21O is not equal to 1, then skip to instruction before VIC36.

VIC35. Was the person who was getting back at you a man or a woman? **(Please choose "both" if you experienced violence from both a man and a woman who fit into this category)** (Choose one)

1 Man
2 Woman
3 Both
8 Refuse to Answer

If VIC21P is not equal to 1, then skip to VIC37.

VIC36. Was the person who was involved in a drug-related situation a man or a woman? **(Please choose "both" if you experienced violence from both a man and a woman who fit into this category)** (Choose one)

1	Man
2	Woman
3	Both
8	Refuse to Answer

VIC37. Has anyone stalked you in the past 6 months?

1	Yes
0	No
8	Refuse to Answer

LEGAL AND SAFETY

Now, I'm going to ask you some questions about crime against you.

LEG1. In the past 6 months, how many times were you a victim of any violent crimes such as assault, rape, mugging, or robbery?

__ __ __	times
998	Refuse to Answer

If LEG1 is equal to 0 or LEG1 is equal to 998, then skip to instruction before LEG3.

LEG2. How many of these did you report to the police?

__ __ __	crimes
998	Refuse to Answer

If LEG2 is greater than LEG1 then Due to your response to an earlier question, your response is too high. Please review. and skip to LEG2.

LEG3. In the past 6 months, how many times were you a victim of any nonviolent crimes such as burglary, theft of your property or money, or being cheated?

__ __ __	times
998	Refuse to Answer

If LEG3 is equal to 0 or LEG3 is equal to 998, then skip to instruction before LEG5.

LEG4. How many of these did you report to the police?

__ __ __	crimes
998	Refuse to Answer

If LEG4 is greater than LEG3 then Due to your response to an earlier question, your response is too high. Please review. and skip to LEG4.

If PAR3 is not equal to 1 and (PAR2BL is equal to 0 or PAR1 is equal to 0), then skip to instruction before LEG8.

LEG5. In the past 6 months, have any of your intimate partners been arrested for harming you?

1	Yes
0	No
8	Refuse to Answer

If LEG5 is not equal to 1, then skip to instruction before LEG8.

LEG6. Were you the one who alerted the police that you needed help?

1	Yes
0	No
8	Refuse to Answer

If LEG6 is not equal to 0, then skip to instruction before LEG8.

LEG7. Are any of the following reasons why you didn't call the police? (Check all that apply) (Check all that apply)

- __ I was afraid of what my partner might do to me if I called the police
- __ I was afraid of what my partner might do to whoever showed up
- __ I was afraid of what my partner might do to him/herself
- __ I didn't want my partner to be arrested
- __ I didn't think that the police needed to be called
- __ I would have, but someone else called the police before I had a chance
- __ My partner physically prevented me from calling the police
- __ It did not occur to me to call the police / there was no reason to call them
- __ Some other reason
- __ Refuse to Answer

If LEG7 is less than 1 and LEG7 is not equal to 98 then Please choose at least one response from the list. and skip to LEG7.

Next, I am going to ask you about your involvement in the criminal justice system.

LEG8. Have you received a police citation in the past 6 months for any reason?

1 Yes
0 No
8 Refuse to Answer

LEG9. Have you been in city or county jail in the past 6 months? (For any reason)

1 Yes
0 No
8 Refuse to Answer

LEG10. Have you attempted to get arrested to get off the street because you had no place to stay in the past 6-months?

1 Yes
0 No
8 Refuse to Answer

LEG11. Have you been in prison during the past 6 months? (For any reason)

1 Yes
0 No
8 Refuse to Answer

If LEG8 is not equal to 1 and LEG9 is not equal to 1 and LEG11 is not equal to 1, then skip to instruction before SU1.

LEG12. Were any of your citations or incarcerations during the past 6 months for any of the following? (Click on each box that applies) (Check all that apply)

__ Drug-related crimes such as possession or distribution
__ Being drunk or high in public
__ Trying to receive money for sex (solicitation)
__ Assault or domestic violence
__ Disturbing the peace
__ Going to the bathroom in public
__ Sleeping outside
__ Loitering
__ Panhandling
__ Some other reason
__ Refuse to Answer

If LEG12 is less than 1 and LEG12 is not equal to 98 then Please choose at least one response from the list. and skip to LEG12.

SUBSTANCE USE

Now, I have some questions about your use of cigarettes, alcohol, and drugs.

SU1. On average, how many cigarettes did you smoke in the past 6 months? (Choose one)

0 Don't smoke or didn't smoke in past 6 months
1 Less than one cigarette per month
2 At least one cigarette per month
3 At least one cigarette per week
4 At least one cigarette per day
5 At least a pack a day
8 Refuse to Answer

SU2. Now, I have some questions about your recent use of alcohol. In the past 6 months, how often did you drink alcohol such as beer, wine, or hard liquor? (Choose one) 1 At least once

2 At least once a week
3 At least once a month
4 Once or twice in the past 6 months
5 Never during the past 6 months
8 Refuse to Answer

If SU2 is equal to 4 or SU2 is equal to 1, then skip to SU5.

If SU2 is equal to 3, then skip to SU4.

If SU2 is equal to 5, then skip to instruction before DRG1.

SU3. On average, how many days in a week did you drink beer, wine, or hard liquor?

__ days
8 Refuse to Answer

Skip to SU5.

SU4. On average, how many days in a month did you drink beer, wine, or hard liquor?

__ __ days
98 Refuse to Answer

SU5. On those [DRKNUM] days when you drank wine, beer, or hard liquor, how many drinks did you usually have each day? One drink is equal to a can of beer, a glass of wine, or a shot of hard alcohol. __ __ __drinl

998 Refuse to Answer

SU6. In the past 6 months, how often did you drink 5 or more drinks in one sitting? Five drinks is equal to 5 cans of beer, one bottle of wine, or a 40oz can of malt liquor. (Choose one) 0 Neve

1 Once or twice in the past 6 months
2 At least once a month
3 At least once a week
4 At least once a day
8 Refuse to Answer

SU7. Now I have a question about alcohol withdrawal. By that, I mean when you tremble, have DTs, have seizures, or when you see, hear, or feel things that aren't there because you haven't drank alcohol or didn't drink enough. On average, how often did you experience these symptoms or others that were related to alcohol withdrawal during the past 6 months? (Choose one)

1 At least once a day
2 At least once a week
3 At least once a month
4 Once or twice in the past 6 months
5 Never during the past 6 months
8 Refuse to Answer

ILLEGAL DRUG USE

Now I have some questions about specific drugs you may have used in the past 6 months.

DRG1. In the past 6 months, how often did you use **marijuana** or pot? (Choose one)

0	Never during the past 6 months
1	Once or twice in the past 6 months
2	At least once a month
3	At least once a week
4	At least once a day
8	Refuse to Answer

If DRG1 is equal to 0 or DRG1 is equal to 8, then skip to DRG3.

DRG2. Do you currently have a prescription for medical marijuana?

1	Yes
0	No
8	Refuse to Answer

DRG3. In the past 6 months, how often did you use **crack**? (Choose one)

0	Never during the past 6 months
1	Once or twice in the past 6 months
2	At least once a month
3	At least once a week
4	At least once a day
8	Refuse to Answer

If DRG3 is equal to 0 or DRG3 is equal to 8, then skip to DRG5.

DRG4. In the past 6 months, did you (Check all that apply) (Check all that apply)

__	Smoke crack?
__	Inject or shoot crack?
__	Snort crack?
__	Take it in some other way?
__	Refuse to Answer

If DRG4 is less than 1 and DRG4 is not equal to 98 then Please choose at least one response from the list. and skip to DRG4.

DRG5. In the past 6 months, how often did you use **powder cocaine**? (Choose one)

0	Never during the past 6 months
1	Once or twice in the past 6 months
2	At least once a month
3	At least once a week
4	At least once a day
8	Refuse to Answer

If DRG5 is equal to 0 or DRG5 is equal to 8, then skip to DRG7.

DRG6. In the past 6 months, did you (Check all that apply) (Check all that apply)

__ Smoke cocaine?
__ Inject or shoot cocaine?
__ Snort cocaine?
__ Take it in some other way?
__ Refuse to Answer

If DRG6 is less than 1 and DRG6 is not equal to 98 then Please choose at least one response from the list. and skip to DRG6.

DRG7. In the past 6 months, how often did you use **methamphetamines** (also known as crystal, speed, crank, glass, and ice)? (Choose one)

0 Never during the past 6 months
1 Once or twice in the past 6 months
2 At least once a month
3 At least once a week
4 At least once a day
8 Refuse to Answer

If DRG7 is equal to 0 or DRG7 is equal to 8, then skip to DRG9.

DRG8. In the past 6 months, did you (Check all that apply) (Check all that apply)

__ Smoke methamphetamines?
__ Inject or shoot methamphetamines?
__ Snort methamphetamines?
__ Take them in some other way?
__ Refuse to Answer

If DRG8 is less than 1 and DRG8 is not equal to 98 then Please choose at least one response from the list. and skip to DRG8.

DRG9. In the past 6 months, how often did you use **heroin**? (Choose one)

0 Never during the past 6 months
1 Once or twice in the past 6 months
2 At least once a month
3 At least once a week
4 At least once a day
8 Refuse to Answer

If DRG9 is equal to 0 or DRG9 is equal to 8, then skip to DRG11.

DRG10. In the past 6 months, did you (Check all that apply) (Check all that apply)

__ Smoke heroin?
__ Inject or shoot heroin?
__ Snort heroin?
__ Take it in some other way?
__ Refuse to Answer

If DRG10 is less than 1 and DRG10 is not equal to 98 then Please choose at least one response from the list. and skip to DRG10.

DRG11. In the past 6 months, how often did you <u>inject</u> any other (illegal or non-prescribed) substance, such as hormones or vitamins? (Choose one)

0 Never during the past 6 months
1 Once or twice in the past 6 months
2 At least once a month
3 At least once a week
4 At least once a day
8 Refuse to Answer

DRG12. In the past 6-months, how often did you use nitrous? (Choose one)

0 Never during the past 6 months
1 Once or twice in the past 6 months
2 At least once a month
3 At least once a week
4 At least once a day
8 Refuse to Answer

DRG13. In the past 6-months, how often did you use valium, xanex or other benzodiazepines? (Choose one)

0 Never during the past 6 months
1 Once or twice in the past 6 months
2 At least once a month
3 At least once a week
4 At least once a day
8 Refuse to Answer

DRG14. In the past 6 months, how often did you <u>use</u> painkillers that weren't prescribed for you, such as Oxycontin, Vicodin, morphine or other opioid painkillers?? (Choose one) 0 Neve

1 Once or twice in the past 6 months
2 At least once a month
3 At least once a week
4 At least once a day
8 Refuse to Answer

DRG15. In the past 6 months, how often did you <u>use</u> other drugs that weren't prescribed for you? (Choose one)

0 Never during the past 6 months
1 Once or twice in the past 6 months
2 At least once a month
3 At least once a week
4 At least once a day
8 Refuse to Answer

If DRUGUSE is equal to 0, then skip to DRG28.

DRG16. Now I have a question about drug withdrawal. By that, I mean feeling sick or anxious, not being able to sleep, or having serious cravings for a drug. On average, how often did you experience drug withdrawal during the past 6 months? (Choose one)

0	Never during the past 6 months
1	A few times in the past 6 months
2	A few times a month
3	A few times a week
4	Every day
8	Refuse to Answer

DRG17. Now I have a question about overdosing on drugs. When I say overdose, I mean taking too much of the drug and your breathing and heart speed up more than usual or you pass out, stop breathing, or feel that your life may be in danger. How often did you overdose on any drug during the past 6 months? (Choose one)

0	Never durring the past 6 months
1	A few times in the past 6 months
2	A few times a month
3	A few times a week
8	Refuse to Answer

DRG18. Were you given Narcan or Naloxone during the past 6 months?

1	Yes
0	No
8	Refuse to Answer

DRG19. Which of the following have been sources of drugs for you in the past 6 months? (Choose all that apply) (Check all that apply)

__	Your intimate partner, &[PINIT4]
__	Another sex partner
__	A dealer
__	A friend
__	A neighbor
__	A relative
__	A Cannabis Club
__	Someone else
__	Refuse to Answer

If DRG19 is less than 1 and DRG19 is not equal to 98 then Please choose at least one response from the list. and skip to DRG19.

DRG20. In the past 6 months, where did you usually get your drugs? (Check all that apply) (Check all that apply)

__ You bought them in the SRO or apartment where you live
__ You bought them in another SRO or apartment
__ You bought them on the street or other public place
__ ¨Someone brought them to you
__ At a Cannabis Club
__ Refuse to Answer

If DRG20 is less than 1 and DRG20 is not equal to 98 then Please choose at least one response from the list. and skip to DRG20.

DRG21. In the past 6 months, where did you usually use drugs? (Check all that apply) (Check all that apply)

__ In your room/place/apartment/house
__ In someone else's room/place/apartment/house
__ On the streets or in alleyways
__ In a park
__ In a shooting gallery or specific place where people go to use drugs
__ In a car
__ Other
__ Refuse to Answer

If DRG21 is less than 1 and DRG21 is not equal to 8 then Please choose at least one response from the list. and skip to DRG21.

DRG22. In the past 6 months, how many people did you usually use drugs with?

__ __ __ people
998 Refuse to Answer

If DRG22 is equal to 0 or DRG22 is equal to 998, then skip to instruction before DRG30.

DRG23. Have you had a main drug use partner within the past 6 months?

1 Yes
0 No
8 Refuse to Answer

If DRG23 is not equal to 1, then skip to instruction before INJ1.

DRG24. What are the initials of that person? __ __ __ __

If PAR5 is not equal to 1 and PAR1 is not equal to 1, then skip to DRG26.

DRG25. Is this the same person you listed as your primary intimate partner?

1 Yes
0 No
8 Refuse to Answer

If DRG25 is equal to 1, then skip to instruction before INJ1.

DRG26. Is this person a man?

1 Yes
0 No
8 Refuse to Answer

DRG27. Have you ever had sex with [Response to DRG24]

1 Yes
0 No
8 Refuse to Answer

If DRUGUSE is equal to 1, then skip to instruction before INJ1.

DRG28. As someone who does not currently use drugs, how often do you see others using drugs? (Choose one)

1 Every day
2 A few times a week
3 A few times a month
4 A few times a year
5 Less than a few times a year
7 Don't Know
8 Refuse to Answer
9 Not Applicable

DRG29. Do you find it difficult to stay away from drugs in your neighborhood?

1 Yes
0 No
8 Refuse to Answer

If PAR5 is not equal to 1 and PAR1 is not equal to 1, then skip to instruction before INJ1.

DRG30. Does your primary intimate partner, [PINIT4] use drugs?

1 Yes
0 No
8 Refuse to Answer

INJECTION DRUG USE

If DRG4B is not equal to 1 and DRG6B is not equal to 1 and DRG8B is not equal to 1 and DRG10B is not equal to 1 and DRG11 is equal to 0 or DRG11 is equal to 8, then skip to instruction before BAD1.

INJ1. In the past 6 months, did you usually reuse the same needle that you had used before?

1 Yes
0 No
8 Refuse to Answer

INJ2. In the past 6 months, did you ever inject with a needle that someone else had already used?

1 Yes
0 No
8 Refuse to Answer

INJ3. In the past 6 months, did you ever give your used needle to someone else?

1 Yes
0 No
8 Refuse to Answer

If INJ2 is not equal to 1, then skip to INJ5.

INJ4. Of those times you reused a needle that had been used by someone else, how often did you clean it with bleach? (Choose one)

0 Never
1 Not very often
2 Sometimes
3 Most of the time
4 Always
8 Refuse to Answer

INJ5. In the past 6 months, did you give away or take from someone else used cotton, used cookers, or dirty water (even if he or she was a close friend or sexual partner)?

1 Yes
0 No
8 Refuse to Answer

If DRGALC is equal to 0, then skip to instruction before STX1.

BELIEFS ABOUT ALCOHOL AND DRUG USE

BAD1. In the past 6 months, did you ever use alcohol or other drugs to:

Relieve long-term physical pain and discomfort?

1 Yes
0 No
8 Refuse to Answer

BAD2. In the past 6 months, did you ever use alcohol or other drugs to:

Relieve depression or anxiety?

1 Yes
0 No
8 Refuse to Answer

BAD3. In the past 6 months, did you ever use alcohol or other drugs to:

Relieve hallucinations or delusions?

1 Yes
0 No
8 Refuse to Answer

BAD4. In the past 6 months, did you ever use alcohol or other drugs to:

Relieve stress and nerves?

1 Yes
0 No
8 Refuse to Answer

BAD5. In the past 6 months, did you ever use alcohol or other drugs to:

Get high or feel euphoric?

1 Yes
0 No
8 Refuse to Answer

BAD6. In the past 6 months, did you ever use alcohol or other drugs to:

Avoid withdrawal?

1 Yes
0 No
8 Refuse to Answer

BAD7. In the past 6 months, did you ever use alcohol or other drugs to:

Make your life tolerable?

1 Yes
0 No
8 Refuse to Answer

BAD8. In the past 6 months, did you ever use alcohol or other drugs to:

Stay awake?

1 Yes
0 No
8 Refuse to Answer

If BAD8 is not equal to 1, then skip to instruction before STX1.

BAD9. Why do you need to stay awake? (Check all that apply)

__ To protect yourself from people you know
__ To protect yourself from people you don't know
__ To protect yourself on the street
__ To work, hustle, or take care of business
__ Some other reason
__ Refuse to Answer

If BAD9 is less than 1 and BAD9 is not equal to 98 then Please choose at least one response from the list. and skip to BAD9.

SUBSTANCE ABUSE TREATMENT

If DRUGUSE is not equal to 1, then skip to instruction before RES1.

STX1. In the past 6 months, have you stayed at least one night in a detox program, hospital, or other type of residential treatment facility where you received treatment for an alcohol or drug problem? Please only count places where you stayed overnight.

1 Yes
0 No
8 Refuse to Answer

STX2. In the past 6 months, have you ever been in a methadone program?

1 Yes
0 No
8 Refuse to Answer

If STX2 is not equal to 1, then skip to STX5.

STX3. Did you receive methadone at SFGH/Ward 93 in the past 6-months?

1 Yes
0 No
8 Refuse to Answer

STX4. During the past 6 months, how long did you participate in the methadone program?

__ MONTHS
__ WEEKS
__ DAYS
8 Refuse to Answer (Months)

STX5. In the past 6 months, have you received any <u>other</u> kind of outpatient treatment, help or support for problems with alcohol or drugs at an agency where you **did not** stay overnight? 1 Yes

0 No
8 Refuse to Answer

If STX5 is not equal to 1, then skip to STX7.

STX6. Did you receive drug treatment at SFGH in the past 6-months? 1 Yes

0 No
8 Refuse to Answer

STX7. In the past 6 months, have you gone to a self-help group such as Alcoholics Anonymous, Narcotics Anonymous or some other type of place?

1 Yes
0 No
8 Refuse to Answer

RESOURCES

Now, I am going to ask you about meeting daily needs.

RES1. In the past 6 months, did you have difficulty finding any of the following? (Check all that apply) (Check all that apply)

- __ Enough to eat
- __ Clothing
- __ A place to wash
- __ A place to use the bathroom
- __ Transportation to someplace you needed to go
- __ Finding shelter
- __ I have not had difficulties
- __ Refuse to Answer

If RES1 is less than 1 and RES1 is not equal to 8 then Please choose at least one response from the list. and skip to RES1.

If RES1Z is equal to 1 and (RES1A is equal to 1 or RES1B is equal to 1 or RES1C is equal to 1 or RES1D is equal to 1 or RES1E is equal to 1) then You have reported both that you've had no difficulties as well as some difficulties. Please review your answer. and skip to RES1.

RES2A. In the past 6 months, did you telephone or go to a battered women's shelter?

1 Yes
0 No
8 Refuse to Answer

If RES2A is not equal to 1, then skip to instruction before RES4.

RES3. Were you able to get the help you needed?

1 Yes
0 No
8 Refuse to Answer

If RES2A is equal to 1, then skip to RES5B.

RES4. What are the reasons you don't use this social service? (Check all that apply) (Check all that apply)

__ I don't know where to go
__ I don't know what to ask for
__ I don't know if I would be accepted there
__ I don't trust the people working there
__ Services aren't offered in my neighborhood
__ Services aren't offered at a good time for me
__ I'm too busy working or taking care of business to go
__ I have to take care of a family member of friend and don't have time
__ My partner doesn't want me to go
__ I've tried the services in the past and they didn't do any good
__ I'm not in one place long enough for it to make a difference
__ I don't think I can afford it
__ I don't need them
__ I use drugs and they have rules against drug use
__ Refuse to Answer

If RES4 is less than 1 and RES4 is not equal to 98 then Please choose at least one response from the list. and skip to RES4.

RES5B. In the past 6 months, did you telephone or use domestic violence services?

1 Yes
0 No
8 Refuse to Answer

If RES5B is not equal to 1, then skip to instruction before RES7.

RES6. Were you able to get the help you needed?

1 Yes
0 No
8 Refuse to Answer

If RES5B is equal to 1, then skip to RES8C.

RES7. What are the reasons you don't use this social service? (Check all that apply) (Check all that apply)

__ I don't know where to go
__ I don't know what to ask for
__ I don't know if I would be accepted there
__ I don't trust the people working there
__ Services aren't offered in my neighborhood
__ Services aren't offered at a good time for me
__ I'm too busy working or taking care of business to go
__ I have to take care of a family member of friend and don't have time
__ My partner doesn't want me to go
__ I've tried the services in the past and they didn't do any good
__ I'm not in one place long enough for it to make a difference
__ I don't think I can afford it
__ I don't need them
__ I use drugs and they have rules against drug use
__ Refuse to Answer

If RES7 is less than 1 and RES7 is not equal to 98 then Please choose at least one response from the list. and skip to RES7.

RES8C. In the past 6 months, did you telephone or go to a food program/soup kitchen?

1 Yes
0 No
8 Refuse to Answer

If RES8C is not equal to 1, then skip to instruction before RES10.

RES9. Were you able to get the help you needed?

1 Yes
0 No
8 Refuse to Answer

If RES8C is equal to 1, then skip to RES11D.

RES10. What are the reasons you don't use this social service? (Check all that apply) (Check all that apply)

__ I don't know where to go
__ I don't know what to ask for
__ I don't know if I would be accepted there
__ I don't trust the people working there
__ Services aren't offered in my neighborhood
__ Services aren't offered at a good time for me
__ I'm too busy working or taking care of business to go
__ I have to take care of a family member of friend and don't have time
__ My partner doesn't want me to go
__ I've tried the services in the past and they didn't do any good
__ I'm not in one place long enough for it to make a difference
__ I don't think I can afford it
__ I don't need them
__ Refuse to Answer

If RES10 is less than 1 and RES10 is not equal to 98 then Please choose at least one response from the list. and skip to RES10.

RES11D. In the past 6 months, did you telephone or receive employment assistance/job training?

1 Yes
0 No
8 Refuse to Answer

If RES11D is not equal to 1, then skip to instruction before RES13.

RES12. Were you able to get the help you needed?

1 Yes
0 No
8 Refuse to Answer

If RES11D is equal to 1, then skip to RES14E.

RES13. What are the reasons you don't use this social service? (Check all that apply) (Check all that apply)

__ I don't know where to go
__ I don't know what to ask for
__ I don't know if I would be accepted there
__ I don't trust the people working there
__ Services aren't offered in my neighborhood
__ Services aren't offered at a good time for me
__ I'm too busy working or taking care of business to go
__ I have to take care of a family member of friend and don't have time
__ My partner doesn't want me to go
__ I've tried the services in the past and they didn't do any good
__ I'm not in one place long enough for it to make a difference
__ I don't think I can afford it
__ I don't need them
__ Refuse to Answer

If RES13 is less than 1 and RES13 is not equal to 98 then Please choose at least one response from the list. and skip to RES13.

RES14E. In the past 6 months, did you telephone or receive legal assistance?

1 Yes
0 No
8 Refuse to Answer

If RES14E is not equal to 1, then skip to instruction before RES16.

RES15. Were you able to get the help you needed?

1 Yes
0 No
8 Refuse to Answer

If RES14E is equal to 1, then skip to instruction before RES17.

RES16. What are the reasons you don't use this social service? (Check all that apply) (Check all that apply)

__ I don't know where to go
__ I don't know what to ask for
__ I don't know if I would be accepted there
__ I don't trust the people working there
__ Services aren't offered in my neighborhood
__ Services aren't offered at a good time for me
__ I'm too busy working or taking care of business to go
__ I have to take care of a family member of friend and don't have time
__ My partner doesn't want me to go
__ I've tried the services in the past and they didn't do any good
__ I'm not in one place long enough for it to make a difference
__ I don't think I can afford it
__ I don't need them
__ Refuse to Answer

If RES16 is less than 1 and RES16 is not equal to 98 then Please choose at least one response from the list. and skip to RES16.

Now I have some questions about your use of the internet.

RES17. In the past 6-months, how often did you use the internet? (Choose one) (Choose one)

0 Never
1 Once or twice
2 A few times
3 At least once a month
4 At least once a week
5 Every day or almost everyday
8 Refuse to Answer

If RES17 is equal to 0 or RES17 is equal to 8, then skip to RES20.

RES18. In the past 6-months, where did you access the internet? (Check all that apply) (Check all that apply)

__ Your home
__ A friend or relative's home
__ Homeless Shelter
__ Public Library
__ A service organization like St. Anthony's
__ A café or other public place that has internet service
__ Your cell phone
__ Your job/place of employment
__ Refuse to Answer

RES19. In the past 6-months, what types of information did you search for on the internet? (Check all that apply) (Check all that apply)

__	Entertainment
__	News
__	E-mail
__	Dating or social networking
__	Health-related information
__	Job search
__	Housing search
__	Legal information
__	Information on current events, interests or blogs
__	Other
__	Refuse to Answer

RES20. In the past 6 months, have you owned a cell phone?

1	Yes
0	No
8	Refuse to Answer

If RES20 is not equal to 1, then skip to RES22.

RES21. During the past 6-months, how often was your cell phone in service? (Choose one)

0	The entire 6 months; it was never off
1	About 3 or 4 weeks of each month
2	About 2 weeks of each month
3	Less than 2 weeks of each month
4	One week of each month
8	Refuse to Answer

RES22. How many other research projects do you currently participate in?

__ __	projects
98	Refuse to Answer

RES23. Congratulations! Thanks for taking the time to answer all of those questions. Please let your interviewer know that you are finished.

__ __ __ __

If RES23 is not equal to Q6 then Please call your interviewer. and skip to RES23.
